# Hybridization patterns in two contact zones of grass snakes reveal a new Central European snake species

**DOI:** 10.1038/s41598-017-07847-9

**Published:** 2017-08-07

**Authors:** Carolin Kindler, Maxime Chèvre, Sylvain Ursenbacher, Wolfgang Böhme, Axel Hille, Daniel Jablonski, Melita Vamberger, Uwe Fritz

**Affiliations:** 1 Museum of Zoology (Museum für Tierkunde), Senckenberg Dresden, A. B. Meyer Building, 01109 Dresden, Germany; 2 Department of Environmental Sciences, Section of Conservation Biology, University of Basel, 4056 Basel, Switzerland; 3 Karch, Passage Maximilien-de-Meuron 6, 2000 Neuchâtel, Switzerland; 4 Zoologisches Forschungsmuseum Alexander Koenig, Adenauerallee 160, 53113 Bonn, Germany; 5 Rosengarten 21, 33605 Bielefeld, Germany; 6 Department of Zoology, Comenius University in Bratislava, Mlynská dolina, Ilkovičova 6, 842 15 Bratislava, Slovakia

## Abstract

Recent studies found major conflicts between traditional taxonomy and genetic differentiation of grass snakes and identified previously unknown secondary contact zones. Until now, little is known about gene flow across these contact zones. Using two mitochondrial markers and 13 microsatellite loci, we examined two contact zones. One, largely corresponding to the Rhine region, involves the western subspecies *Natrix natrix helvetica* and the eastern subspecies *N. n. natrix*, whereas in the other, more easterly, contact zone two lineages meet that are currently identified with *N. n. natrix* and *N. n. persa*. This second contact zone runs across Central Europe to the southern Balkans. Our analyses reveal that the western contact zone is narrow, with parapatrically distributed mitochondrial lineages and limited, largely unidirectional nuclear gene flow. In contrast, the eastern contact zone is very wide, with massive nuclear admixture and broadly overlapping mitochondrial lineages. In combination with additional lines of evidence (morphology, phylogeny, divergence times), we conclude that these differences reflect different stages in the speciation process and that *Natrix helvetica* should be regarded as a distinct species. We suggest a nomenclatural framework for presently recognized grass snake taxa and highlight the need for reconciling the conflicts between genetics and taxonomy.

## Introduction

Even though species delimitation became a Renaissance issue in zoology, with new approaches being developed for assessing species boundaries^1–5^, the validity of many approaches largely depends on the underlying species concept. Currently, there are more than 30 species concepts distinguished, with an ever increasing number^6^. While the application of different concepts lead to a taxonomic inflation for some regions and some groups, the number of Central European vertebrate species remained generally stable, suggesting their diversity is well understood. An exception to that rule might be bats^7, 8^. With respect to Central European snakes, the number of recognized species did not change for more than a century^9–15^, even though some southern European taxa, like *Elaphe sauromates* ^16^ *, Macroprotodon brevis* and *M. mauritanicus* ^17, 18^, *Malpolon insignitus* ^19^, *Natrix astreptophora* ^20^, *Vipera graeca* ^21^, and *Zamenis lineatus* ^22^, have been elevated to species status within the last two decades and a new species of viper (*Vipera walser*) has been recently discovered in the Alps^23^.

Grass snakes (*Natrix natrix* sensu lato) are the most abundant and one of the most widely distributed snake species of the Palaearctic region^24, 25^. For a long time, little was known about their genetic and phylogeographic structuring. Based on external morphology, many subspecies have traditionally been recognized^25, 26^, suggestive of pronounced phylogeographic structuring. Indeed, in a pioneering nearly range-wide study using mitochondrial DNA (mtDNA), not less than 16 distinct genetic lineages were identified^26^. However, the majority of these lineages conflicts with previously recognized subspecies and only two lineages match with morphologically defined taxa. In phylogenetic analyses, these lineages correspond to 16 terminal clades that cluster in three major clades (Supplementary Fig. S1). One of these major clades matches with the Ibero-Maghrebian taxon *astreptophora*, which had traditionally been recognized as a subspecies of *N. natrix*. In the face of virtually lacking gene flow with the geographically neighbouring taxon (*N. n. helvetica*) and concordant morphological and genetic evidence, Ibero-Maghrebian grass snakes have recently been split off as the distinct species *N. astreptophora* ^20^.

In agreement with earlier morphological investigations^27^, the recent phylogeographic assessment of grass snakes^26^ revealed a contact zone of two deeply divergent mitochondrial lineages for the Rhine region, with an unexpectedly clear-cut parapatric distribution pattern. The involved lineages match with what is currently identified with *N. n. helvetica* and *N. n. natrix*. Another unexpected discovery was that the distribution ranges of *N. n. natrix* in Central Europe and *N. n. persa* in the Balkan Peninsula comprise a previously unknown, more easterly located, contact zone of two distinct lineages, which conflict with morphological taxon delimitation and occur across the distribution ranges of *N. n. natrix* and *N. n. persa* ^26^. In contrast to the situation in the Rhine region, the haplotypes of these two eastern lineages occur in wide sympatry and represent lineages that are placed in phylogenetic analyses in the same major clade, while the mitochondrial lineage of *N. n. helvetica* belongs to another one of the three major clades (Supplementary Fig. S1). According to molecular clock calculations^28^, the clade containing *N. n. helvetica* diverged from the eastern lineages 7.3–8.2 million years ago, while the two eastern lineages are with 5.1–5.9 million years significantly younger.

The present study aims at examining and comparing differentiation and gene flow across the two contact zones of genetic lineages of different age and phylogenetic hierarchy. For doing so, the previous sampling^26^ was increased fourfold and mitochondrial DNA data (1,983 bp) were combined with evidence from 13 highly polymorphic nuclear microsatellite loci.

## Materials and Methods

### Sampling and laboratory procedures

**Figure 1:**
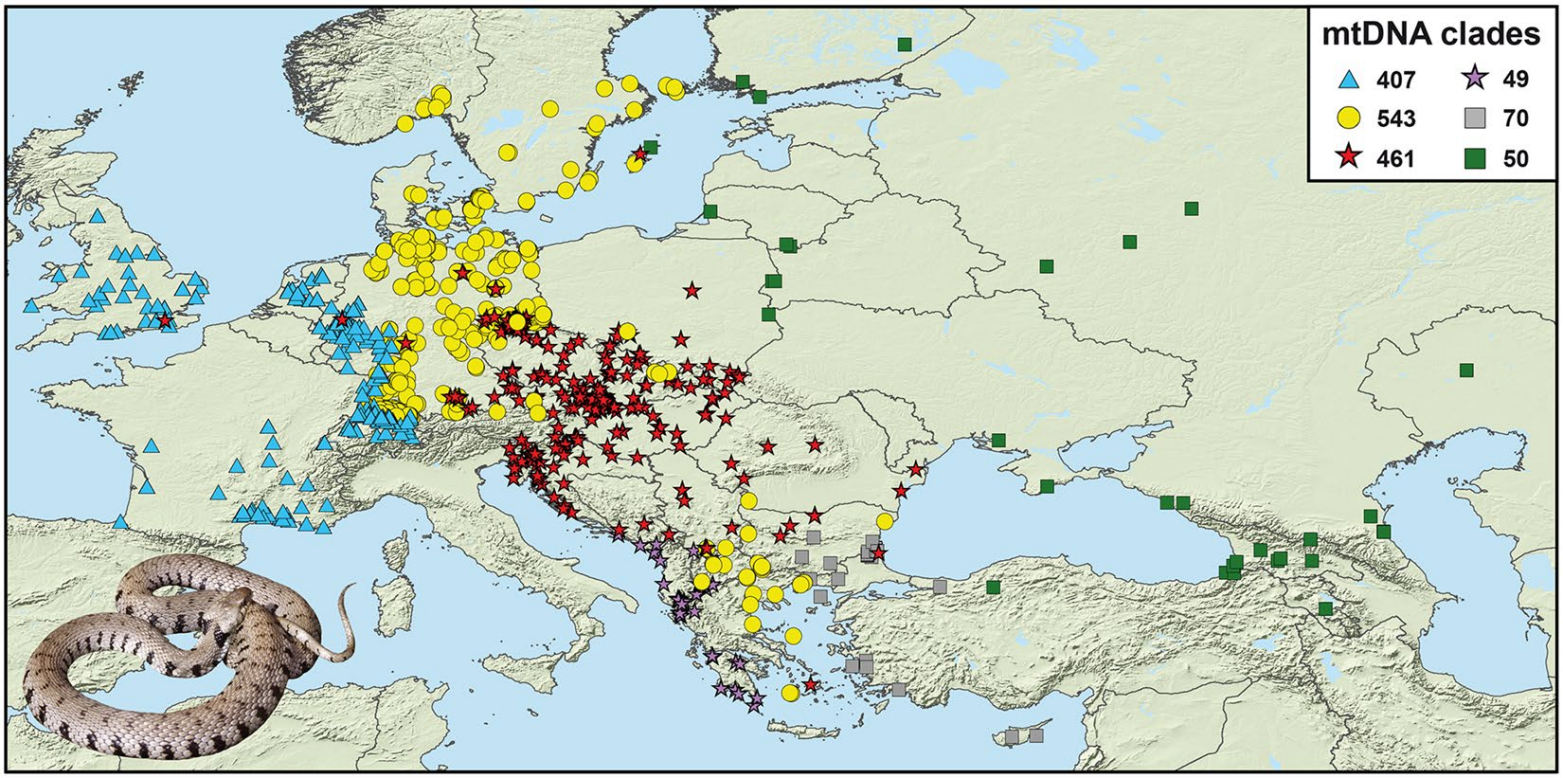
Distribution of mitochondrial lineages of 1,580 grass snakes used in this study. Total sample size of each clade shown in the legend. Eight allochthonous grass snakes with haplotypes of Italian lineages caught in southern Great Britain and Hesse, Germany, not shown. Map was created using arcgis 10.2 (http://www.esri.com/arcgis) and adobe illustrator CS6 (http://www.adobe.com/products/illustrator.html). Inset: *Natrix natrix helvetica* (Linz am Rhein, Germany); photo: Wolfgang Böhme.

The focus of the present study lies on two contact zones of distinct genetic lineages of grass snakes (Fig. 1), one in the Rhine region, involving *Natrix natrix helvetica* (‘blue lineage’) and the nominotypical subspecies (‘yellow lineage’) and another contact zone further in the east, which was identified for the first time in a previous study^26^. This second contact zone runs across Central Europe to the southern Balkans and concerns two genetic lineages (‘red’ and ‘yellow lineages’), which occur within the distribution ranges of *N. n. natrix* and *N. n. persa*. However, neither of these subspecies is congruent with the two genetic lineages, and both lineages occur within the range of either taxon and beyond, suggesting that a taxonomic revision is required^26^. Only one of these lineages, the ‘yellow lineage’, occurs naturally in the Rhine region, i.e. in the contact zone with *N. n. helvetica*.

In total, 1,603 samples (shed skins, saliva samples, tissues from roadkills and museum specimens) were used in the present study. No snakes were sacrificed for the present study. All sampling and methods were carried out in accordance with relevant guidelines, regulations and best ethical and experimental practice of the Senckenberg Nature Research Society. Two mitochondrial DNA fragments (partial ND4 gene plus adjacent DNA coding for tRNAs = ND4 + tRNAs, below termed for simplicity ND4, and cytochrome *b* gene = cyt *b*) were sequenced (866 bp and 1,117 bp, respectively). Mitochondrial sequences of 391 specimens were available from previous studies^20, 26, 29^ and merged with new data for 1,197 grass snakes. For 15 samples, mtDNA could not be sequenced. In addition, samples were genotyped at 13 polymorphic nuclear microsatellite loci. Laboratory procedures for mtDNA and microsatellites followed previous studies^20, 26^. The microsatellite data of 1,484 samples included those for 31 grass snakes from a previous study^20^. For 119 samples, for which mtDNA sequences were available, no microsatellite data could be generated. Approximately 200 samples from Switzerland were studied in the laboratory of the University of Basel, whereas the majority of samples was processed in the laboratory of Senckenberg Dresden. Fragment lengths of the two data sets were calibrated using 31 samples processed in both laboratories. For detailed sample information, see Supplementary Table S1.

### Mitochondrial sequence analyses and networks

Mitochondrial sequences were aligned using bioedit 7.0.9.0^30^, resulting in an 866-bp-long alignment of 1,550 ND4 sequences and an 1,117-bp-long alignment of 1,313 cyt *b* sequences. The mitochondrial lineage of each new sample was identified by running exploratory Maximum Likelihood (ML) analyses using raxml 7.2.8^31^ including previously published data^20, 26, 29^, the GTR + G model and a fast ML search with 100 bootstrap values. Then, for the lineages involved in the studied contact zones, alignments of each mtDNA block were examined using popart (http://popart.otago.ac.nz) and the implemented parsimony network algorithm of tcs ^32^. Based on haplotypes, uncorrected *p* distances (means) were calculated using MEGA 7.0.21^33^ and the pairwise deletion option.

### Genetic cluster analysis, inferring hybrid status and PCA

All 13 microsatellite loci were tested for Hardy-Weinberg equilibrium (HWE) and linkage equilibrium using arlequin 3.5.1.2^34^. The presence of null alleles was examined using micro-checker 2.2.3^35^. There was no evidence for linkage disequilibrium, null alleles or a deviation from HWE. Microsatellite data were then analyzed with the unsupervised Bayesian clustering approach of structure 2.3.4^36, 37^ using the admixture model and correlated allele frequencies. structure searches in the data set for partitions that are, as far as possible, in linkage equilibrium and HWE. The Monte Carlo Markov chains ran for 1 million generations, including a burn-in of 250,000 generations. Calculations were repeated ten times for *K*s ranging from 1 to 10. The optimal number of clusters was determined using the Δ*K* method^38^ with the software structure harvester ^39^. structure results were visualized using distruct 1.1^40^.

In the southern Balkans and the Baltic Sea region, additional genetic lineages occur^26, 29^. These lineages are expected to contribute to nuclear genomic admixture, which is why a stepwise approach was applied to assess their genetic impact and to single out the extent of admixture between the red and yellow lineages. This approach takes into account that structure is known to identify only the uppermost hierarchical level of genetic partitioning^38^, i.e. the clusters reflect the most differentiated genetic units. Accordingly, a first calculation comprising all available samples resulted in two clusters, one corresponding to *helvetica* (blue lineage) and the other to all other lineages.

To explore which individuals represent *helvetica* hybrids, hybrid genotypes were modelled using hybridlab 1.0^41^. For doing so, 20 non-admixed representatives of each lineage occurring in the Rhine contact zone were selected (blue and yellow lineages) as pure parental genotypes. Using these data, 20 genotypes of each hybrid class (F_1_, F_2_ and two backcrosses) were inferred and the simulated hybrid data were then subjected to structure analyses, together with the data of the 20 pure grass snakes of each cluster, to obtain a threshold for *Q* values for distinguishing pure animals, hybrids and backcrosses. Based on this threshold, all genotypes with genetic impact of *helvetica* were removed and a second structure run included then only the remaining samples. With this run, admixture of the red and yellow lineages with the other geographically neighbouring lineages (lilac, grey, and green lineages; Fig. 1) was explored. Then, all samples with an impact from other lineages > 5% were eliminated and the remaining data (only red and yellow genotypes and their hybrids) were subjected to another structure run. This data set was also examined using hybridlab to distinguish pure and hybrid samples. For this hybridlab run, samples from geographically distant populations were selected that originated in regions where either only the red or the yellow lineage occurs (Scandinavia vs. Austria and Slovakia).

In addition, for the microsatellite data of the structure clusters, population genetic diversity values, pairwise *F*_ST_ values and Analyses of Molecular Variance (AMOVAs) were calculated using convert 1.31^42^, arlequin ^34^ and fstat 2.9.3.2^43^. Admixed individuals were excluded.

Microsatellite data were also examined using Principal Component Analyses (PCA) as implemented in the R package adegenet ^44^ to assess the distinctiveness of the genetic lineages without underlying population genetic presumptions. Two different PCAs were run, one including genotypes of all samples of the blue, red and yellow lineages and their hybrids and another one with samples of the yellow and red lineage without influence of *helvetica* and the adjacent eastern lineages. Obviously introduced individuals were excluded.

### Cline analyses

To examine gene flow across contact zones, cline analyses were calculated for microsatellite and mtDNA data using the R package hzar ^45^. This software fits molecular genetic data to classical equilibrium cline models using the Metropolis-Hastings Markov chain Monte Carlo algorithm. Cline fitting was performed by adding geographical information of sampling sites to genetic information. To acknowledge for mountain ranges, two transects were selected. The transect for the contact zone in the Rhine region ran from southern France to northeastern Germany (1,200 km) and the transect for the eastern contact zone, from northern Germany to northern Hungary (1,000 km). The eastern transect was not extended to the southern Balkans because the grass snakes are there genetically impacted by other genetic lineages. Using arcgis 10.2 (http://www.esri.com/arcgis), samples were arbitrarily pooled by dividing each transect into segments of 10 km length. Samples within 50 km left and right of each segment were assigned to one sampling unit.

For microsatellite data, the mean proportion *Q* of cluster membership (as inferred by structure) for each pooled collection site was then calculated. For mitochondrial data, the frequency of haplotypes of the blue lineage (western contact zone) or the yellow lineage (eastern contact zone) was used. These four data sets (mtDNA and microsatellites for each contact zone) were processed independently. Using a burn-in of 10,000 iterations, followed by additional 90,000 iterations, fifteen implemented models were fitted to the mean proportions of cluster membership or haplotype frequencies. The best cline model was then selected based on the lowest AIC score (Supplementary Table S2) and the corresponding Maximum Likelihood clines. Observed frequency data were plotted over the associated fuzzy cline regions (95% credible cline regions).

## Results

### Mitochondrial phylogeography and haplotypes

Our fourfold sampling (Fig. 1 and Supplementary Fig. S2) confirmed and refined previous findings^26^, in particular that the blue lineage (*Natrix natrix helvetica*) meets with the yellow lineage (currently identified with *N. n. natrix*) in a secondary contact zone in the Rhine region, and that another, more easterly and much wider, contact zone of the yellow and red lineages runs across Central Europe to the Balkans. The latter two lineages are distributed within the ranges of what is currently identified with the grass snake subspecies *N. n. natrix* and *N. n. persa*, but these lineages conflict with morphology-based taxonomy so that neither the distribution ranges of the lineages and subspecies nor morphology and genetics are congruent^26^. Rather, each subspecies corresponds to several distinct mitochondrial lineages and some of these lineages are shared between the two subspecies. Thus, there is obviously a pronounced conflict between morphological variation and genetics. Whereas the geographical distribution of haplotypes of the blue and yellow lineages abuts, with virtually no sympatric occurrences (Supplementary Fig. S2), haplotypes of the yellow and red lineages widely overlap in Central Europe (Fig. 1), in a region corresponding to central, eastern and southern Germany, southern Poland, Austria, the Czech Republic, and Slovakia. However, further southeastwards, only haplotypes of the red lineage occur and in the southern Balkans, only haplotypes of the yellow lineage are recorded (Fig. 1).

While the vast majority of our 1,588 samples represents native grass snakes, there are some obvious cases of translocated individuals, like a few isolated records of the red lineage within the range of *N. n. helvetica* (Great Britain, Rhine region). One population in the Neander valley close to Düsseldorf, Germany, seems to consist exclusively of such allochthonous grass snakes and has been suspected to be introduced for a long time^46, 47^. In addition to these non-native grass snakes, eight individuals of other mitochondrial lineages from the Mediterranean (Italy) were found in southern Great Britain and Hesse, Germany (not shown in Fig. 1). We cannot completely exclude that also our new record of a third mitochondrial lineage on the island of Gotland (green lineage; Fig. 1) refers to an introduced grass snake. However, when it is considered that grass snakes colonized Gotland via transoceanic dispersal^29^ and that now all three lineages occurring all around the Baltic Sea have been recorded from Gotland, their natural occurrence seems possible there.

**Figure 2:**
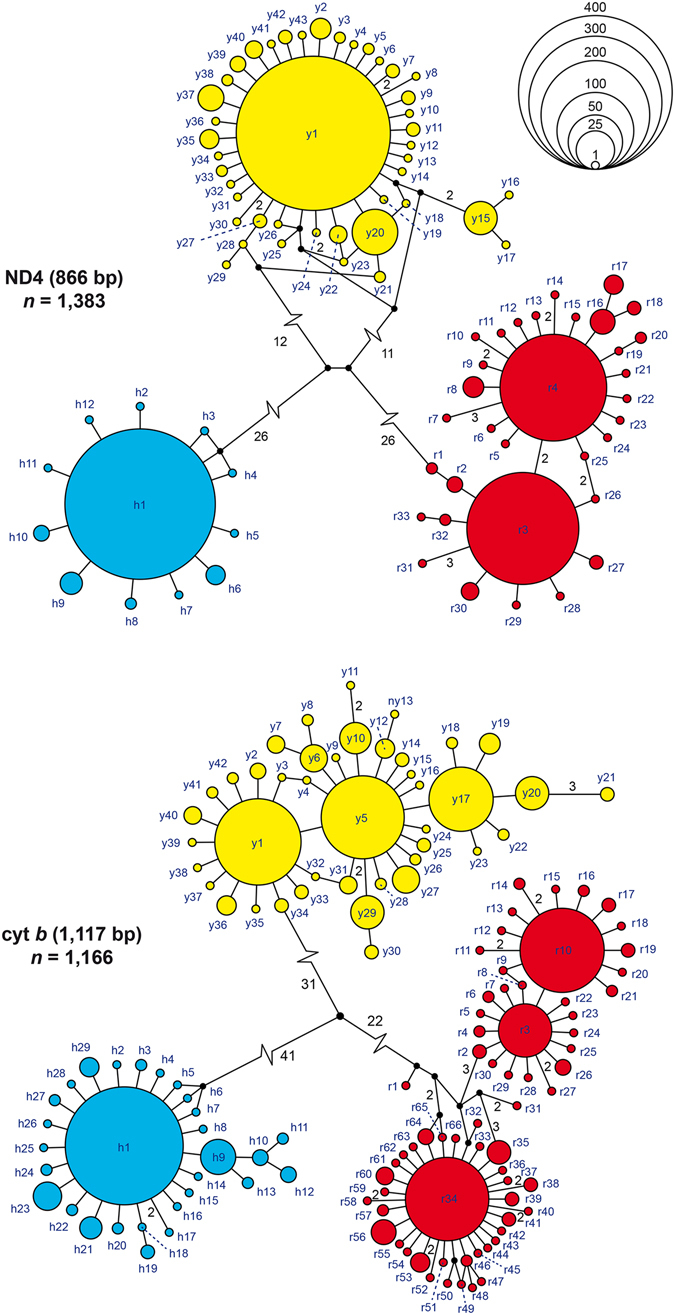
Parsimony networks of mtDNA sequences. Symbol sizes reflect haplotype frequencies. Small black circles are missing node haplotypes; each line connecting two haplotypes corresponds to one mutation step, if not otherwise indicated by numbers. Haplotype colours correspond to lineages, i.e. *Natrix natrix helvetica* (h) in blue; eastern lineages in yellow (y) and in red (r).

In parsimony network analyses, each lineage corresponded to a highly distinct haplotype cluster. For ND4 (Fig. 2, top), there are 12 haplotypes in the blue lineage, 43 haplotypes in the yellow lineage, and 33 haplotypes in the red lineage. Haplotypes of the yellow and red lineages differed by a minimum of 40 mutational steps. Haplotypes of the blue lineage (*N. n. helvetica*) were separated from the yellow haplotype cluster by at least 40 mutational steps and from the red cluster by a minimum of 54 steps. With only 12 haplotypes differing by maximally two mutation steps, there was distinctly less variation in the blue lineage compared to the two eastern lineages. In the yellow lineage, 43 haplotypes with maximally 15 mutations occurred; in the red lineage, 33 haplotypes differing by maximally 10 mutations.

For cyt *b* (Fig. 2, bottom) a similar pattern emerged. The blue lineage differed by a minimum of 73 mutations from the yellow lineage and by 64 mutations from the red lineage. The yellow and red haplotype clusters were connected by a minimum of 53 mutational steps. Compared to ND4, there was distinctly more variation observed, with 29 haplotypes in the blue lineage and 66 haplotypes in the red lineage. The yellow lineage comprised 42 haplotypes. Haplotypes of the blue lineage differed by a maximum of five mutations, haplotypes of the yellow lineage by a maximum of nine mutations and haplotypes of the red cluster by a maximum of 16 mutations. European Nucleotide Archive (ENA) accession numbers for haplotypes are listed in Supplementary Table S3.

For ND4, the blue lineage differed on average from the yellow lineage by an uncorrected *p* distance of 5.03% and from the red lineage, by 5.88%; the divergence between the yellow and red lineages was 5.16%. For cyt *b*, mean *p* distances between the blue, yellow and red lineages were 6.90% and 6.39%, respectively; the yellow and red lineages differed by 5.48%.

### Genotyping and admixture

**Figure 3:**
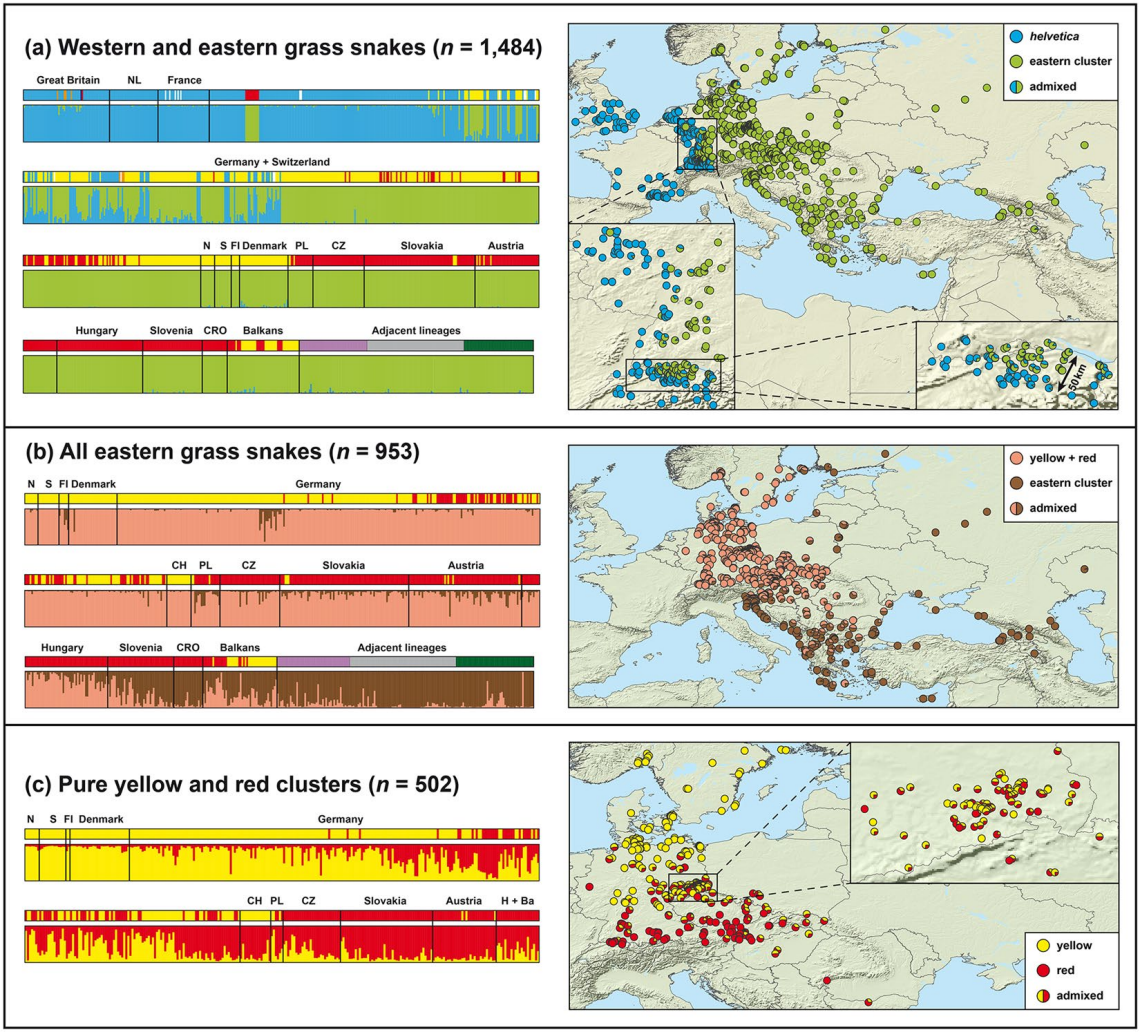
Genotypic structuring of grass snakes. On the left, the mitochondrial lineage of each sample is shown above the structure diagrams, with haplotypes of *Natrix natrix helvetica* indicated in blue and haplotypes of the eastern lineages in colours corresponding to Fig. 1 (yellow, red, lilac, grey, green; white = missing data). In (**a**), orange and dark blue corresponds to non-native snakes (Italian lineages). Samples in structure diagrams are arranged within each country from west to east (**a**) or from north to south (**b**,**c**). In structure diagrams, an individual sample is represented by a vertical bar reflecting its inferred ancestry. In (**a**), the blue cluster corresponds to *N. n. helvetica* and the light green cluster to all other lineages. The isolated red/light green block (first row) represents the allochthonous population from the Neander valley, Germany. In (**b**), samples with genetic impact of *helvetica* are excluded. The pink cluster corresponds to samples from the yellow and red lineages. Brown percentages indicate genetic impact of adjacent lineages (lilac, grey, green). In (**c**) only samples from the yellow and red lineages and their hybrids, without genetic signatures of other lineages, were processed. Country abbreviations: Ba – Balkans (Albania, Bosnia and Herzegovina, Montenegro, Serbia, Kosovo, Former Yugoslav Republic of Macedonia, Romania, Bulgaria, and Greece), CH – Switzerland, CRO – Croatia, CZ – Czech Republic, FI – Finland, H – Hungary, N – Norway, NL – Netherlands, PL – Poland, S – Sweden. Maps were created using arcgis 10.2 (http://www.esri.com/arcgis) and adobe illustrator CS6 (http://www.adobe.com/products/ illustrator.html).

The 13 studied microsatellite loci were highly polymorphic, with allele numbers ranging from 12 to 39 per locus (Supplementary Table S4) and a total allele number of 285. For the complete data set including *Natrix natrix helvetica* and all five eastern lineages, the Δ*K* method suggested two as the optimal number of clusters (Supplementary Fig. S3a). One of these clusters represented *helvetica*, and the other contained all eastern lineages (Fig. 3a). The assignment of the eastern lineages to only one cluster matched with the close phylogenetic relationship of their mtDNA lineages^26^. According to an AMOVA using microsatellite data, 59.84% of the molecular variance occurred within and 40.16% between the two clusters, corresponding to an *F*_ST_ value of 0.40. The high distinctiveness of both clusters was also supported by a large number of private alleles, especially for the eastern cluster (Supplementary Table S5).

In general, the *helvetica* cluster matched well with the mitochondrial clade (Fig. 3). Genotypic introgression occurs largely unidirectional from *helvetica* into the eastern cluster. Most hybrids between *helvetica* and the eastern cluster originate from northern Switzerland, where the sampling is very comprehensive and dense, with approximately 200 samples from the contact zone. However, this dense sampling also revealed that the contact zone is narrow, with hybrid signatures occurring only in a maximally 50-km-wide strip (Fig. 3a, right). The allochthonous population of grass snakes of the red lineage in the Neander valley is clearly assigned to the eastern cluster, in agreement with mitochondrial haplotypes (Fig. 3a, left), and without admixture with neighbouring *helvetica* populations.

The second structure run (Fig. 3b) included the eastern lineages without impact of *helvetica*. Based on the hybridlab results (Supplementary Table S6), only samples with an eastern cluster membership of at least 95% were considered in that analysis, for which *K* = 2 was again the best solution (Supplementary Fig. S3b). One cluster (pink) embraced the yellow and red lineages and another one (brown) the grey, lilac and green lineages. Many southern samples of the yellow and red lineages, especially from Slovenia, Croatia and the southern Balkans, showed a high degree of admixture with the brown cluster. This admixture area largely corresponds to those regions where only haplotypes of the yellow and red lineages are present.

Samples with admixed ancestry with the brown cluster >5% were excluded from the third structure analysis (Fig. 3c), for which the optimal number of clusters was again *K* = 2 (Supplementary Fig. S3c). Now, one cluster corresponded to the yellow and the other to the red lineage. According to the hybridlab results (Supplementary Table S6), grass snakes with at least 80% cluster membership were treated as ‘pure yellow’ and with at least 83% as ‘pure red’. Introgression was common in both directions, indicating massive gene flow across hundreds of kilometres (Fig. 3c, right), including regions where exclusively or predominantly mitochondrial haplotypes of one lineage are present. For the yellow and red clusters, 89.18% of the molecular variance occurred within, and only 10.82% between the two clusters, equalling an *F*_ST_ value of 0.11 (Supplementary Table S5).

**Figure 4:**
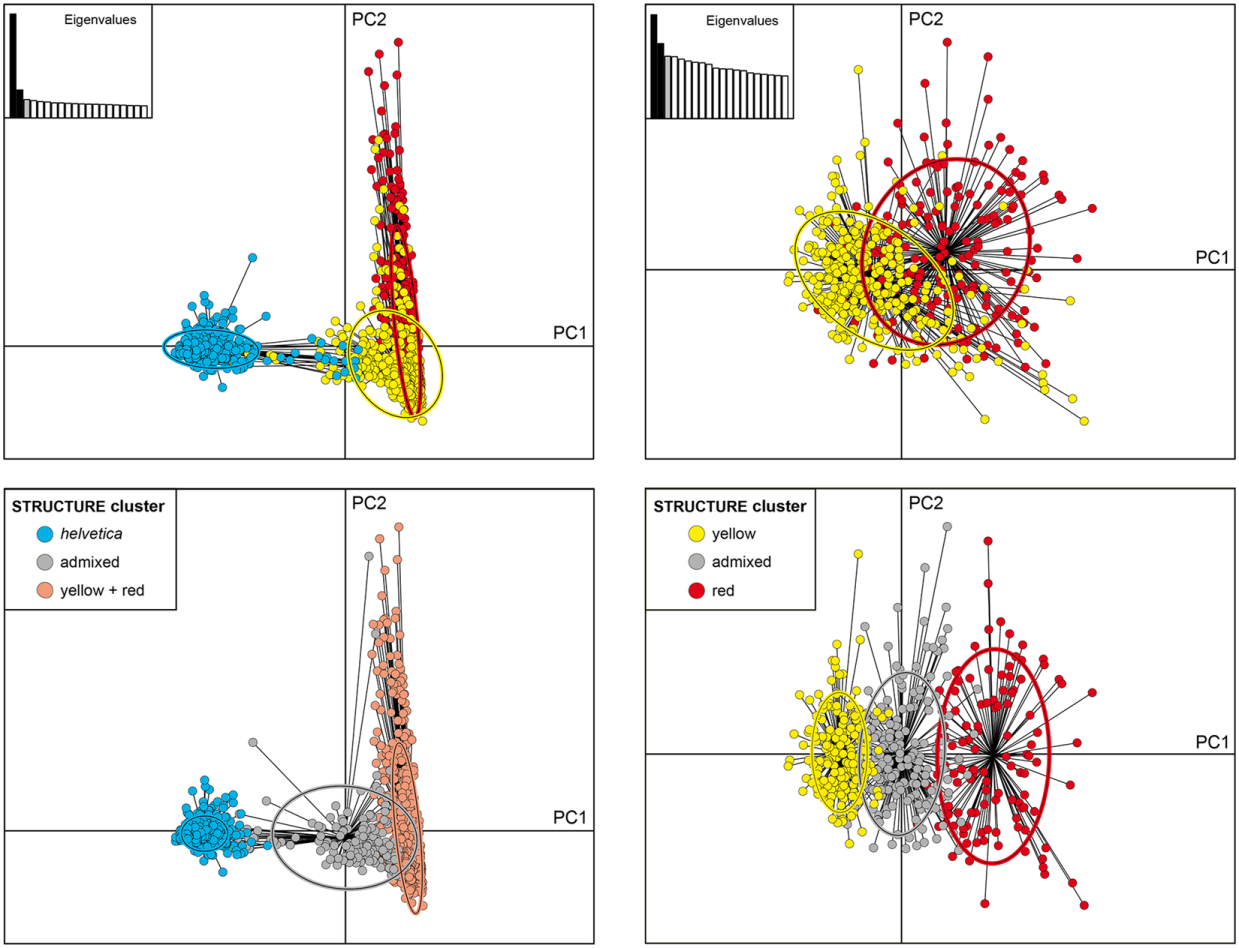
PCA axes 1–2 for microsatellite data. Samples are coloured according to mitochondrial lineages (top) or structure clusters (bottom). Admixed individuals were identified according to hybridlab results. PCAs for the yellow and red lineages correspond to the samples from Fig. 3c. Non-native samples were excluded. The oval outlines represent 95% confidential intervals. For *helvetica* and the eastern lineages (left) the x axis explains 16.6% and the y axis 4.5% of variation. For the eastern lineages (right) the x axis explains 3.8% and the y axis 2.9% of variation. Analyses along axes 1–3 produced nearly identical results (see Supplementary Fig. S4).

The PCAs using microsatellite data (Fig. 4) are in line with the structure analyses in that the blue lineage (*N. n. helvetica*) was highly distinct from the yellow and red lineages, with some mismatches reflecting mitochondrial introgression mainly from *helvetica* into the eastern group. In contrast, the eastern lineages showed weak differentiation and massive overlap. The PCAs corroborate furthermore that our definition of admixed individuals is appropriate because hybrids were intermediate also in the PCA, independently from population affiliation, HWE or linkage equilibrium.

### Cline analyses

**Figure 5:**
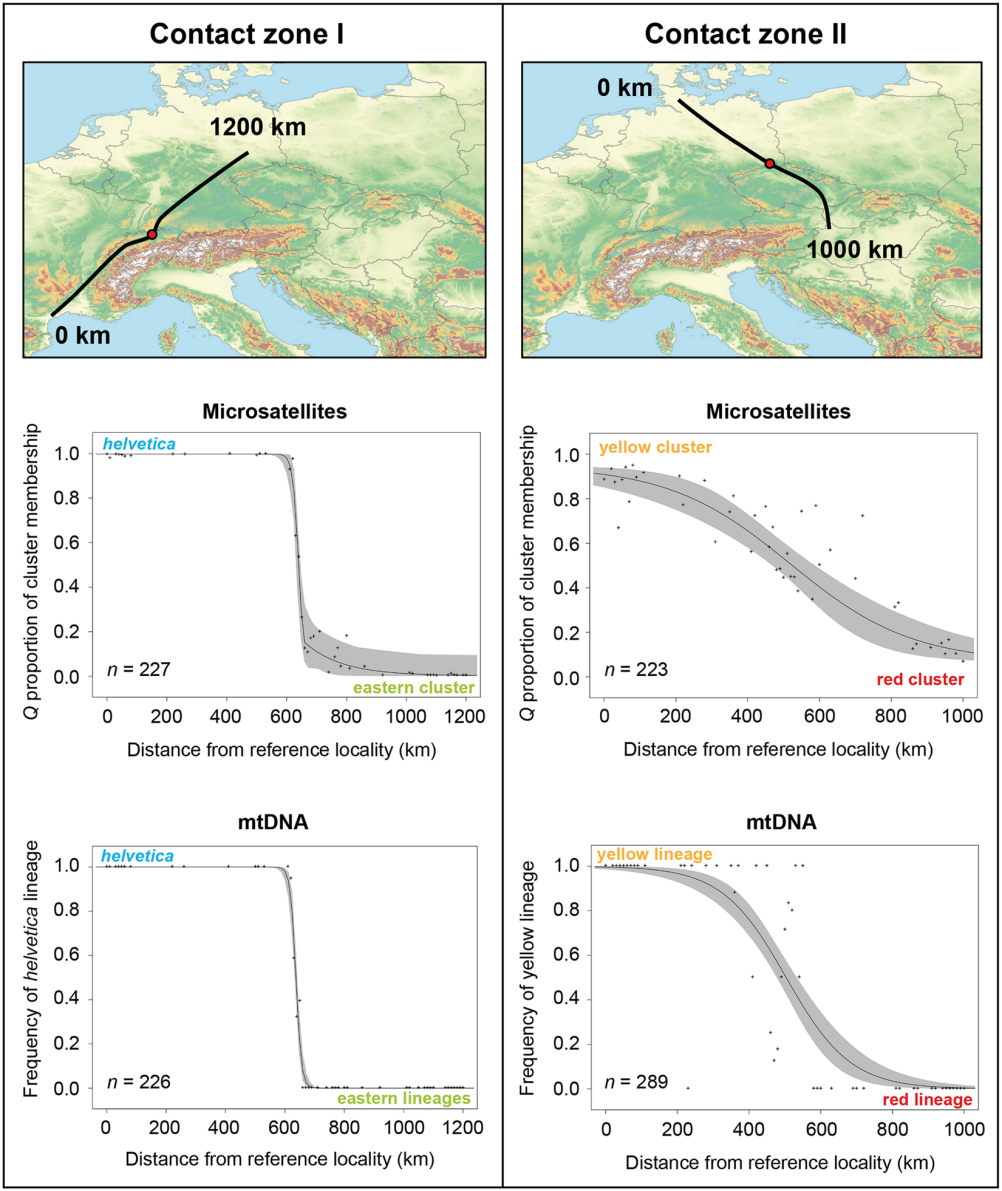
Cline analyses of mitochondrial DNA and microsatellite data. Transects (top) through the two different contact zones of grass snake lineages (*helvetica*/eastern lineages – left; yellow/red lineages – right) and associated Maximum Likelihood clines for microsatellites (centre) and mtDNA (bottom). Grey: fuzzy 95% credible cline region. Red points (top) indicate cline centres. Maps were created using arcgis 10.2 (http://www.esri.com/arcgis) and adobe illustrator CS6 (http://www.adobe.com/products/illustrator.html).

The cline analyses revealed completely different patterns in the two contact zones (Fig. 5). For the western contact zone (contact zone I), the cline is concordant and very steep for both marker systems. For microsatellites, the cline centre was estimated to be located 639.9 km (95% confidence interval: 635.1–644.2 km) north-east from the starting point in southern France with a cline width of 39.4 km (24.4–59.6 km). For mitochondrial data, the cline centre was revealed almost at the same point, at 637.2 km (632.9–641.2 km), with a similar cline width of 37.5 km (27.3–53.6 km). A much smoother cline was found for the contact zone of the yellow and red lineages (contact zone II). The cline centre for microsatellites was located 518.7 km (454.5–590.4 km) distant from the reference site in northern Germany with a considerable cline width of 677.1 km (404.5–1,009.6 km). The width for the mtDNA cline was with 358.3 km (261.5–489.7 km) approximately half as wide as the microsatellite cline. The location of the centre was nearly identical at 503.8 km (474.6–537.0 km) from the reference site.

## Discussion

Hybrid zones are regions in which genetically distinct populations meet and produce hybrid offspring^48^. They can be interpreted as ‘windows on the evolutionary process’^49^ and ‘natural laboratories for evolutionary studies’^50^. Hybridization can provide important insights into divergence and speciation processes^50–56^ and, thus, contributes to a better understanding of evolution. Based on extensive sampling of approximately 1,600 grass snakes and using a powerful data set of 13 microsatellite loci and two mitochondrial markers, this study presents a fine-scale analysis of gene flow across two secondary contact zones of grass snake lineages.

There are significantly different patterns of gene flow. While gene flow in the contact zone of *N. n. helvetica* and the ‘yellow lineage’ is limited and largely unidirectional from *helvetica* into the ‘yellow lineage’, with a cline width of less than 50 km in the contact zone, gene flow in the contact zone of the ‘yellow’ and ‘red lineages’ is extensive and in both directions. This contact zone is wide (cline widths for microsatellites and mtDNA approx. 680 km and 360 km), and the ‘yellow’ and ‘red lineages’ seem to be panmictic there (Fig. 3).

Our data provide evidence for hybridization in the contact zone of *N. n. helvetica* and the yellow lineage. The extent of admixture there clearly exceeds the negligible gene flow between *helvetica* and *N. astreptophora* in southwestern France. The latter taxon is now regarded as a distinct species^20^. Nevertheless, there also seems to be a barrier against gene flow between *helvetica* and eastern grass snakes. Their contact zone is characterized by steep clines for both marker systems (Fig. 5, left). In addition, the two clusters are separated by a high *F*_ST_ value of 0.40 for microsatellites. Due to the parapatric distribution of mitochondrial haplotypes along the contact zone (Supplementary Fig. S2) combined with mainly unidirectional genotypic introgression (Fig. 3), it can be concluded that gene flow is mainly mediated by *helvetica* males. Asymmetrical introgression is not unusual in hybridizing taxa^57^. According to our structure and hybridlab analyses, F_1_ hybrids between *helvetica* and eastern grass snakes are rare. Parental genotypes in the contact zone, together with backcrosses, correspond to a bimodal hybrid zone and an advanced speciation process^58^. Other examples for such bimodal hybrid zones in Europe include, for instance, fire-bellied toads (*Bombina bombina*, *B. variegata* ^59, 60^), crested and marbled newts (*Triturus cristatus*, *T. marmoratus* ^61, 62^; *T. carnifex*, *T. cristatus*, *T. dobrogicus* ^63^), pond turtles (*Emys orbicularis*, *E. trinacris* ^55^), and wall lizards (*Podarcis bocagei*, *P. carbonelli* ^64^), taxa which are all regarded as distinct species. Generally, a steep cline across a narrow hybrid zone suggests lower hybrid fitness and selection against hybrids^48, 65–67^, corresponding to intrinsic isolating mechanisms, which may also include assortative mating.

A completely different pattern is represented by the contact zone of the two eastern grass snake lineages, the ‘red’ and the ‘yellow lineage’. Virtually all individuals are admixed there, indicating a unimodal hybrid zone. Cytonuclear discordance is frequent, in particular, ‘red’ or mainly ‘red’ genotypes are often combined with ‘yellow’ haplotypes (Fig. 3c). The contact zone covers a broad geographical area and is characterized by smooth wide clines (Fig. 5, right); the *F*_ST_ value of 0.11 (microsatellites) of the involved lineages is low. Similar, also geographically wide-ranging, admixture among distinct taxa is observed for instance in European pond turtles (*Emys orbicularis galloitalica*, *E. o. hellenica* ^55^) and rabbits (*Oryctolagus cuniculus cuniculus*, *O. c. algirus* ^68^). Enigmatic is the more or less exclusive presence of mitochondrial haplotypes of the ‘red lineage’ in the central part of the contact zone and the more or less exclusive presence of ‘yellow haplotypes’ in the southernmost part (Fig. 1), despite massive nuclear admixture (Fig. 3). Unlike in the very north, where the exclusive presence of yellow haplotypes can be easily explained by early Holocene colonization and subsequent high-density blocking^69^, the absence of one mitochondrial lineage in the hybrid zone could be related to selective pressure against one mitochondrial lineage; a finding requiring further research. The fact that additional genetic lineages are involved in these parts of the contact zone further complicates the matter.

In summary, we found good agreement between the studied mitochondrial lineages and nuclear genotypes of grass snakes. Using 13 highly polymorphic microsatellite loci, distinct clusters were revealed that correspond to previously identified mitochondrial lineages^26^. However, we discovered very different gene flow patterns, with steep clines and a narrow contact zone for *N. n. helvetica* and the ‘yellow lineage’ and a wide contact zone with smooth clines for the ‘yellow’ and ‘red lineages’. According to mtDNA, the involved lineages are of different phylogenetic hierarchy and age (Supplementary Fig. S1): *Natrix natrix helvetica* belongs to another major clade than the ‘yellow’ and ‘red lineages’, which are placed in phylogenetic analyses into the same major clade^26^. *Natrix natrix helvetica* diverged from the two eastern lineages 7.3–8.2 million years ago, whereas the yellow and red lineages split only 5.1–5.9 million years ago^28^. With respect to nuclear genotypes, *N. n. helvetica* and the ‘yellow lineage’ differ by an *F*_ST_ value of 0.40, while the ‘yellow’ and the ‘red lineage’ differ by 0.11. When these values are compared to the *F*_ST_ value of 0.27 for *N. n. helvetica* and *N. astreptophora* (based on the same loci^20^), this together with the limited gene flow in the narrow contact zone raises the question whether *N. n. helvetica* represents a distinct species and not only a subspecies.

Species conceptualization and delimitation is a complicated issue. In particular, it is difficult to distinguish whether observed differences are on the species or population level^70^. Moreover, different species concepts can lead to different conclusions about species status, and there are currently more than 30 species concepts used^6^. However, to bypass conflicts between different species concepts, it has been suggested to unite their common elements and to characterize species primarily as independent evolutionary lineages^71, 72^. Morphology, reproductive isolation, ecological niches or reciprocal monophyly are understood as different lines of evidence for species status^71^.

It is important that these lines of evidence emerge at different times in the speciation process^71^. Regarding reproductive isolation, species boundaries are known to be ‘semipermeable’^49, 73, 74^ and approximately 10% of animal species are known to hybridize^75^. Speciation may occur despite continuous gene flow^76–81^ and high abundances of hybrids within a hybrid zone are not uncommon^48^. Obviously, reproductive isolation in nature is a matter of degree^82^, with the complete lack of interbreeding and hybridization representing only the most extreme condition^6^. Hybridization between sister species in narrow contact zones is also known from other European snake species, like *Vipera aspis* and *V. latastei* in Spain^83^. Therefore, the limited gene flow between *N. n. helvetica* and the ‘yellow lineage’ fits in that pattern and is not contradicting species status.

Considering the largely unidirectional gene flow from *N. n. helvetica* into the ‘yellow lineage’ in a narrow contact zone, the morphological distinctness of *N. n. helvetica* ^25^, its placement in another deeply divergent clade than eastern grass snake lineages^26^, and the considerable age of its mitochondrial lineage^28^ (Supplementary Fig. S1), we propose to elevate this taxon to full species level and to recognize *Natrix helvetica* (Lacepède, 1789) as a distinct species.

In contrast, we regard the ‘yellow’ and ‘red lineages’ as conspecific, representing a less advanced stage in the speciation process. This assessment is supported by their lacking morphological differentiation, their wide hybrid zone with panmictic large-scale gene flow, the placement of their younger mitochondrial lineages in the same more inclusive clade^26, 28^ (Supplementary Fig. S1) and a low *F*_ST_ value compared to the differentiation of *N. helvetica*. Our structure analyses (Fig. 3b) also provide evidence that the ‘yellow’ and ‘red lineages’ admix on broad scale with other lineages from the same clade in the southern Balkans^26^, supporting their conspecificity. These lineages are currently identified in the Balkans with *N. n. persa*, and some of them with *N. n. natrix* in more northerly regions^14, 25, 26^.

Nomenclaturally, the recognition of *N. helvetica* as a full species necessitates that all nominal subspecies assigned to the same major clade in phylogenetic analyses^26^ have to be transferred to *N. helvetica*, resulting in a revised taxonomy (Table 1). Fortunately, the many previously described mismatches between morphologically defined taxa and genetic lineages^26^ refer only to taxa within, but not across, the newly delimited species, so that the suggested taxonomy does not contribute to further nomenclatural confusion, but reflects deep genetic divergences and discontinuities much better than before. However, we wish to underline that further research is needed for reconciling the conflicts between genetics and morphology of the individual subspecies within each of the three grass snake species.

**Table 1:** Current and proposed taxonomy for grass snakes.

Current taxonomy	Proposed taxonomy
*Natrix astreptophora*	*Natrix astreptophora*
*Natrix natrix natrix*	*Natrix natrix natrix*
*Natrix natrix cypriaca*	*Natrix natrix cypriaca*
*Natrix natrix fusca*	*Natrix natrix fusca*
*Natrix natrix gotlandica*	*Natrix natrix gotlandica*
*Natrix natrix persa*	*Natrix natrix persa*
*Natrix natrix schweizeri*	*Natrix natrix schweizeri*
*Natrix natrix scutata*	*Natrix natrix scutata*
*Natrix natrix syriaca*	*Natrix natrix syriaca*
*Natrix natrix helvetica*	*Natrix helvetica helvetica*
*Natrix natrix cetti*	*Natrix helvetica cetti*
*Natrix natrix corsa*	*Natrix helvetica corsa*
*Natrix natrix lanzai*	*Natrix helvetica lanzai*
*Natrix natrix sicula*	*Natrix helvetica sicula*

## Electronic supplementary material

Supplementary Information
