# Supplementary material for: Hybridization patterns in two contact zones of grass snakes reveal a new Central European snake species
Source: Sci Rep. 2017 Aug 7;7:7378. doi: 10.1038/s41598-017-07847-9 (PMC5547120; doi:10.1038/s41598-017-07847-9)
Supplement: Supplementary file 1 — Supplementary Information [file 41598_2017_7847_MOESM1_ESM.pdf]

Hybridization patterns in two contact zones of grass snakes reveal a new Central European snake species

Carolin Kindler, Maxime Chèvre, Sylvain Ursenbacher, Wolfgang Böhme,

Axel Hille, Daniel Jablonski, Melita Vamberger & Uwe Fritz

Scientific Reports

Supplementary Information

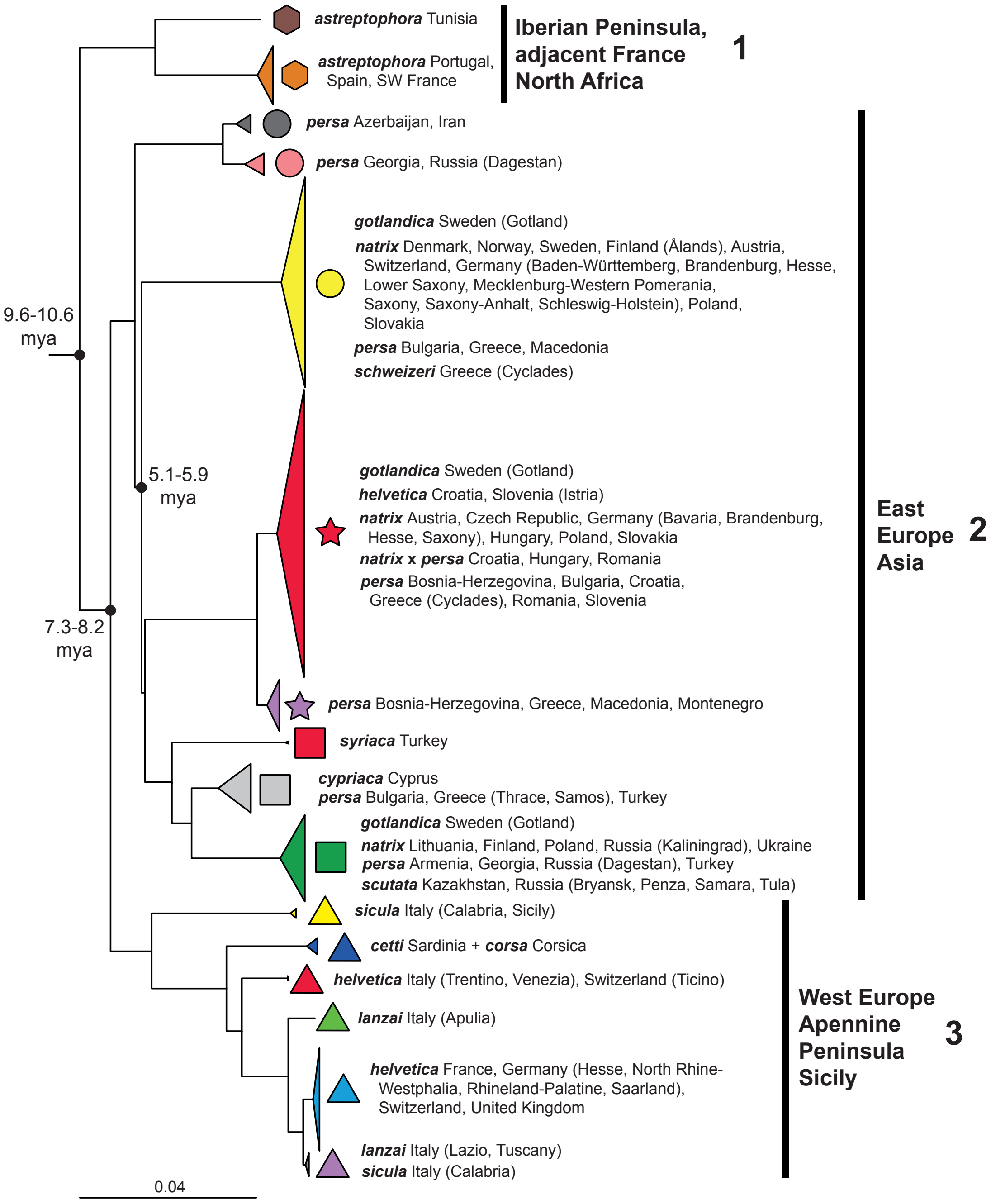

Figure S1. Simplified mitochondrial phylogeny for grass snakes (from Kindler *et al*. 2013, 2014 based on 1,984 bp of ND4 and cyt *b*). The three major clades 1-3 are highlighted; numbers at nodes indicate split ages according to Fritz *et al.* (2012). The new record for Gotland, Sweden, is added (clade with green square). Note the many mismatches between morphologically defined taxa and mitochondrial clades. The traditional identification of grass snakes from Istria (Kabisch 1999; Kreiner 2007) is in error and also conflicts with morphology (unpubl. data). The figure was created using ADOBE ILLUSTRATOR CS6 (http://www.adobe.com/products/illustrator.html).

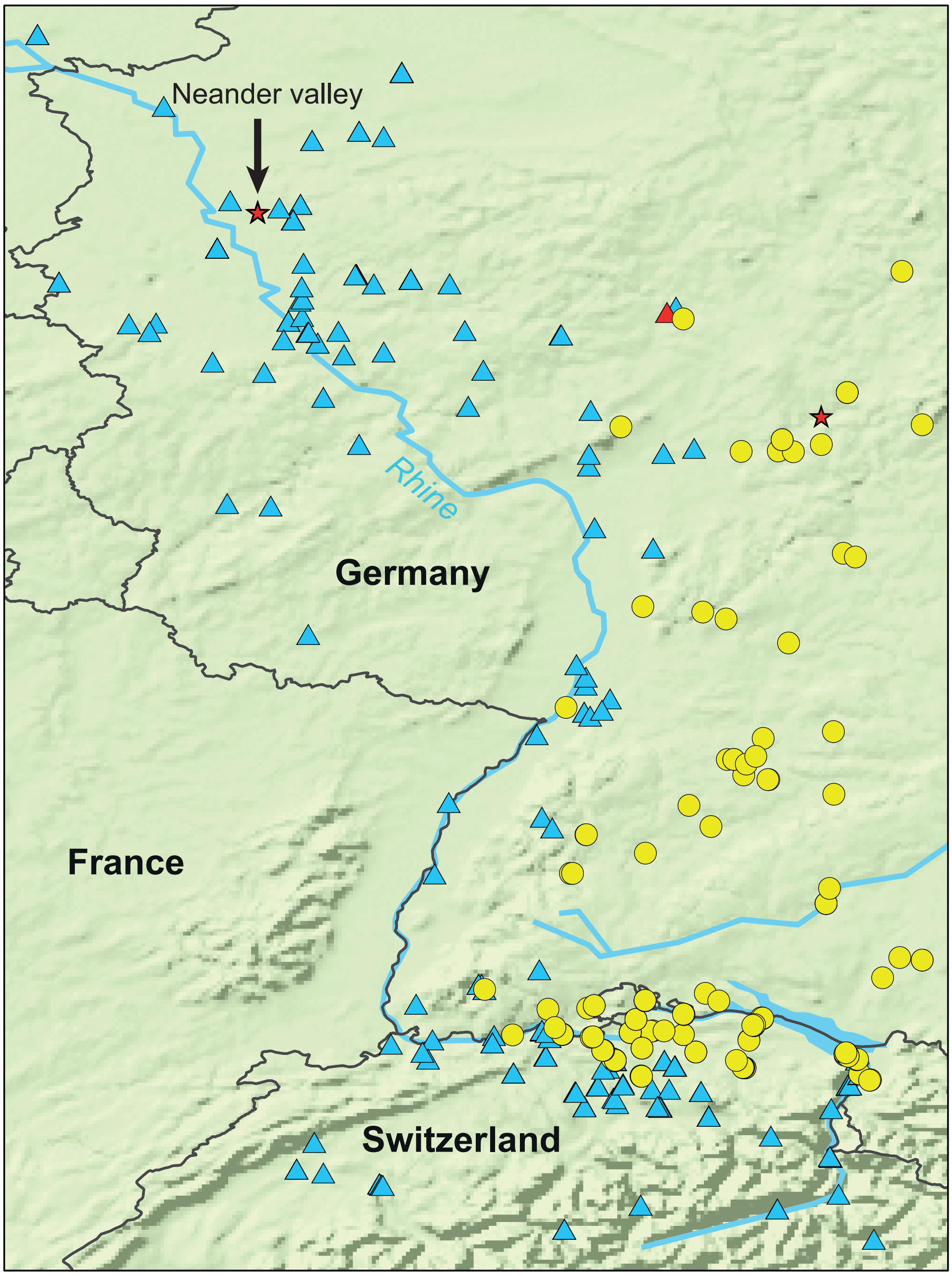

Figure S2. Distribution of mitochondrial haplotypes in the western contact zone. Symbols correspond to Figures 1 and S1. Arrow highlights allochthonous population of the ‘red lineage’ in the Neander valley. Red triangle represents a non-native lineage from northern Italy. Map was created using ARCGIS 10.2 (http://www.esri.com/arcgis) and ADOBE ILLUSTRATOR CS6 (http://www.adobe.com/products/illustrator.html).

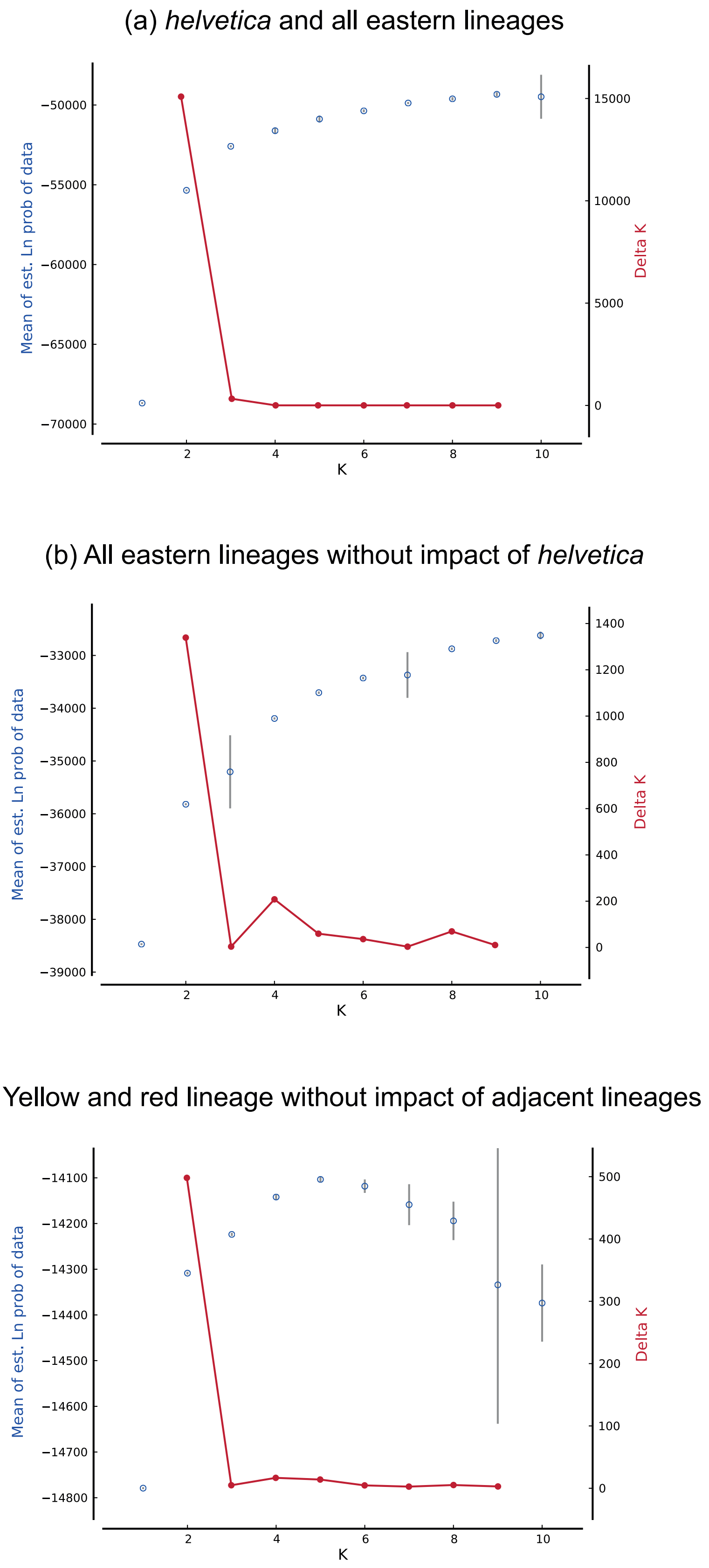

Figure S3. **Δ*K* values and posterior probabilities for STRUCTURE runs for three data sets using STRUCTURE HARVESTER (Earl & vonHoldt, 2012).** The modal value is always at *K*=2. *K*=1 could be excluded because of the higher posterior probabilities for *K*=2.

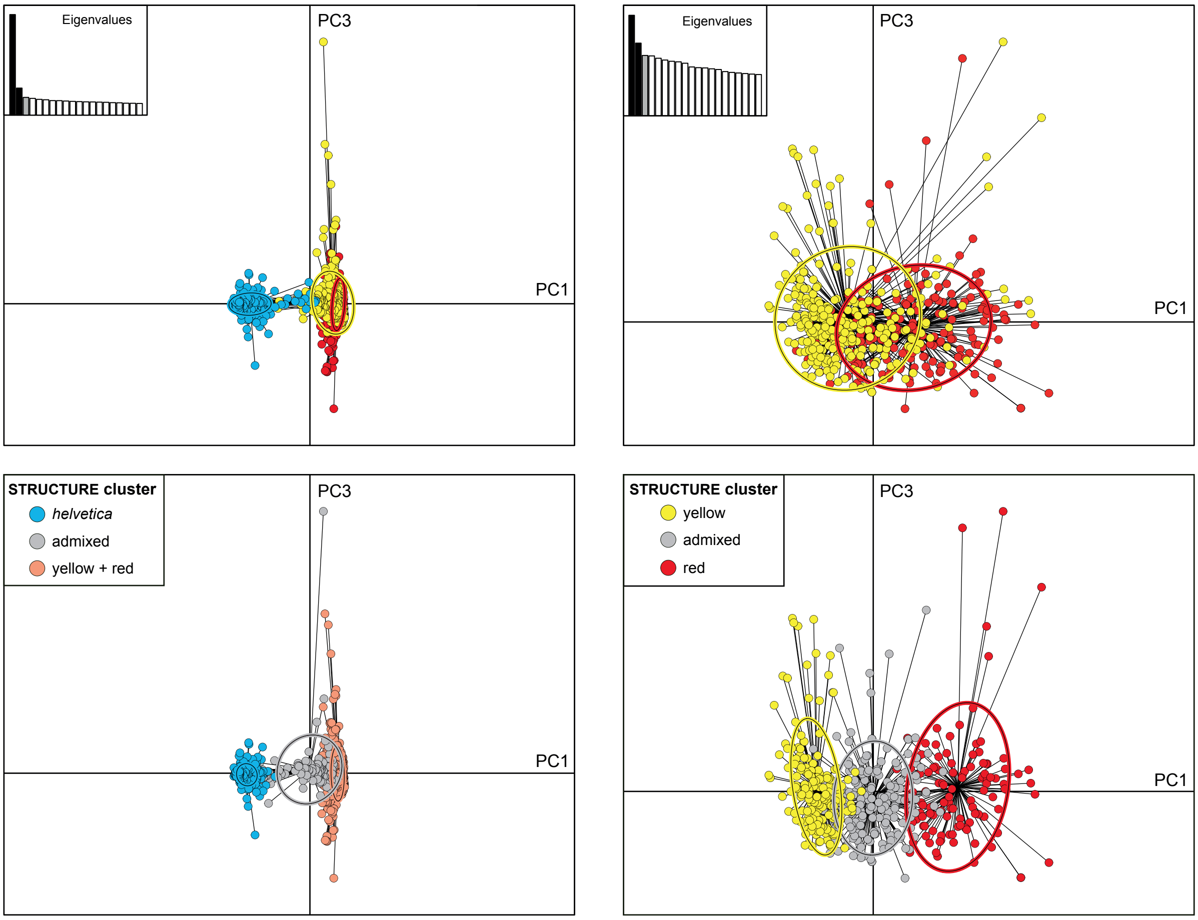

Figure S4. PCA axes 1–3 for microsatellite data of both contact zones. Samples are coloured according to mtDNA lineages (top) and STRUCTURE clusters (bottom). Admixed individuals were categorized according to HYBRIDLAB results. PCAs for the yellow and red lineages correspond to the samples from Figure 3c. Non-native samples were excluded. The oval outlines represent 95% confidential intervals. For *helvetica* and the eastern lineages (left), the x axis explains 16.6% and the y axis 2.9% of variation. For the eastern lineages (right), the x axis explains 3.8% and the y axis 2.4% of variation.

Table S1. Samples studied. Blue vouchers correspond to samples processed at the University of Basel. MtDNA data colour-coded according to genetic lineage. Accession numbers indicate samples from previous studies which were too short for an unambiguous assignment to haplotypes. STRUCTURE clusters colour-coded according to legend on the right.

Colour code:

helvetica cluster	cluster yellow + red	yellow cluster
eastern cluster	cluster adjacent lineages	red cluster
admixed	admixed	admixed

		mtDNA data			Microsatellite data			
**Voucher**	**Locality**	**mtDNA clade**	**ND4 haplotype**	**cyt *b* haplotype**	**First STRUCTURE run**	**Second STRUCTURE run**	**Third STRUCTURE run**	**Reference**
**MTD T 10627**	Albania: Boboshticë	5	l11	l10	0.00	0.39	-	This study
**MTD T 13543**	Albania: Cajupit pass	5	l2	l10	0.04	0.04	-	This study
**MTD T 13544**	Albania: Cajupit pass	5	l2	l8	0.00	0.71	-	This study
**MTD T 14777**	Albania: Divjakë-Karavastë National Park	5	l7	l15	0.03	0.04	-	This study
**MTD T 11602**	Albania: Fitore	5	l5	l8	0.01	0.18	-	This study
**MTD T 13003**	Albania: Grabovë	5	l2	l16	0.01	0.21	-	This study
**MTD T 11603**	Albania: Koder	5	l6	-	0.05	0.04	-	This study
**MTD T 13004**	Albania: near Memaliaj: Schendli Mountains	5	l3	-	0.00	0.02	-	This study
**MTD T 13005**	Albania: near Memaliaj: Schendli Mountains	5	l2	-	0.01	0.11	-	This study
**MTD T 13006**	Albania: near Memaliaj: Schendli Mountains	5	l9	l17	0.10	-	-	This study
**MTD T 13001**	Albania: near Sukë: Trebeshine Mountains	5	l2	l10	0.25	-	-	This study
**MTD T 13002**	Albania: near Sukë: Trebeshine Mountains	5	l2	l19	0.01	0.02	-	This study
**MTD T 11598**	Albania: near Sukë: Trebeshinë Mountains	5	l20	l20	0.01	0.33	-	This study
**MTD T 11599**	Albania: Shalës	5	l6	l10	0.00	0.16	-	This study
**MTD T 11600**	Albania: Shalës	5	l6	l21	0.00	0.10	-	This study
**MTD T 14757**	Armenia: Nshkhark	8	gn10	gn17	0.02	0.03	-	This study
**MTD T 14758**	Armenia: Nshkhark	8	gn10	gn17	0.01	0.02	-	This study
**MTD T 14759**	Armenia: Nshkhark	8	gn10	gn17	0.09	-	-	This study
**ZFMK 65105**	Austria: Burgenland: Andau	4	r30	r3	0.00	0.80	-	Kindler *et al*. (2013)
**NHMW 39266 (2)**	Austria: Burgenland: Apetlon	4	r3	r18	0.00	0.98	0.05	This study
**ZFMK 68656**	Austria: Burgenland: Apetlon	4	r4	r34	0.00	0.91	-	Kindler *et al*. (2013)
**ZFMK 55995**	Austria: Burgenland: Breitenbrunn	4	r4	r34	0.00	0.83	-	Kindler *et al*. (2013)
**NHMW 39030 (2)**	Austria: Burgenland: Eisenstadt	4	r4	r34	0.00	0.96	0.15	This study
**ZFMK 51746**	Austria: Burgenland: Hölle	4	-	r35	0.00	0.93	-	Kindler *et al*. (2013)
**ZFMK 74926**	Austria: Burgenland: Illmitz	4	r4	r34	0.00	0.92	-	Kindler *et al*. (2013)
**ZFMK 83775**	Austria: Burgenland: Illmitz	4	-	r65	0.00	0.96	0.15	Kindler *et al*. (2013)
**ZFMK 65687**	Austria: Burgenland: Jois	4	r3	r10	0.00	0.69	-	Kindler *et al*. (2013)
**NHMW 38644**	Austria: Burgenland: Lake Neusiedl	4	r3	-	0.00	0.92	-	This study
**MTD T 11991**	Austria: Burgenland: Lake Neusiedl: Illmitz	4	r4	-	0.00	0.56	-	This study
**MTD T 12062**	Austria: Burgenland: Lake Neusiedl: Illmitz	4	r4	-	0.01	0.52	-	This study
**MTD T 12063**	Austria: Burgenland: Lake Neusiedl: Illmitz	4	r4	-	0.00	0.86	-	This study
**NHMW 38809**	Austria: Burgenland: Lake Neusiedl: Illmitz	4	r4	r34	0.00	0.79	-	This study
**NHMW 39436 (2)**	Austria: Burgenland: Lake Neusiedl: Illmitz	4	r4	r43	0.00	0.97	0.09	This study
**NHMW 40024 (1)**	Austria: Burgenland: Lake Neusiedl: Illmitz	4	r4	r35	0.00	0.89	-	This study
**MTD T 11986**	Austria: Burgenland: Lake Neusiedl: Jois	4	r4	r53	0.00	0.90	-	This study
**MTD T 11987**	Austria: Burgenland: Lake Neusiedl: Jois	4	r4	r39	0.00	0.95	0.09	This study
**MTD T 11988**	Austria: Burgenland: Lake Neusiedl: Jois	4	r30	-	0.00	0.78	-	This study
**MTD T 11989**	Austria: Burgenland: Lake Neusiedl: Jois	4	r4	r34	0.00	0.98	0.18	This study
**MTD T 11990**	Austria: Burgenland: Lake Neusiedl: Jois	4	r4	-	-	-	-	This study
**MTD T 11992**	Austria: Burgenland: Lake Neusiedl: Jois	4	r4	r56	0.00	0.96	0.06	This study
**MTD T 12058**	Austria: Burgenland: Lake Neusiedl: Jois	4	r4	-	-	-	-	This study
**MTD T 12059**	Austria: Burgenland: Lake Neusiedl: Jois	4	r4	-	-	-	-	This study
**MTD T 12060**	Austria: Burgenland: Lake Neusiedl: Jois	4	r4	-	0.00	0.91	-	This study
**MTD T 12061**	Austria: Burgenland: Lake Neusiedl: Jois	4	r4	-	0.00	0.61	-	This study
**MTD T 12064**	Austria: Burgenland: Lake Neusiedl: Jois	4	r4	r53	0.00	0.79	-	This study
**MTD T 12065**	Austria: Burgenland: Lake Neusiedl: Jois	4	r4	r10	0.00	0.82	-	This study
**MTD T 12066**	Austria: Burgenland: Lake Neusiedl: Jois	4	r4	-	0.00	0.96	0.06	This study
**MTD T 12067**	Austria: Burgenland: Lake Neusiedl: Jois	4	r3	r10	0.00	0.57	-	This study
**MTD T 12068**	Austria: Burgenland: Lake Neusiedl: Jois	4	r13	-	-	-	-	This study
**MTD T 12069**	Austria: Burgenland: Lake Neusiedl: Jois	4	r4	-	0.00	0.98	0.18	This study
**MTD T 12070**	Austria: Burgenland: Lake Neusiedl: Jois	4	r4	-	-	-	-	This study
**MTD T 12071**	Austria: Burgenland: Lake Neusiedl: Jois	4	r4	-	0.00	0.96	0.10	This study
**MTD T 12072**	Austria: Burgenland: Lake Neusiedl: Jois	4	r4	-	-	-	-	This study
**MTD T 12073**	Austria: Burgenland: Lake Neusiedl: Jois	4	r4	-	-	-	-	This study
**MTD T 12074**	Austria: Burgenland: Lake Neusiedl: Jois	4	r4	-	0.00	0.90	-	This study
**MTD T 12075**	Austria: Burgenland: Lake Neusiedl: Jois	4	r4	-	-	-	-	This study
**MTD T 12076**	Austria: Burgenland: Lake Neusiedl: Jois	4	r30	r10	0.00	0.79	-	This study
**MTD T 12077**	Austria: Burgenland: Lake Neusiedl: Jois	4	r3	-	0.00	0.69	-	This study
**MTD T 12078**	Austria: Burgenland: Lake Neusiedl: Jois	4	r4	-	-	-	-	This study
**NHMW 39266 (1)**	Austria: Burgenland: Lake Neusiedl: Neusiedl am See	4	r4	r56	0.00	0.98	0.22	This study
**ZFMK 51670**	Austria: Burgenland: Weiden am See	4	r10	r34	0.00	0.67	-	Kindler *et al*. (2013)
**ZFMK 91242**	Austria: Burgenland: Zitzmannsdorfer Wiesen	4	r4	r35	0.00	0.89	-	Kindler *et al*. (2013)
**MTD T 11964**	Austria: Carinthia: Lake Faak	4	r4	-	-	-	-	This study
**NHMW 39030 (1)**	Austria: Lower Austria: Aspersdorf	4	r4	r34	0.00	0.99	0.14	This study
**MTD T 9912**	Austria: Lower Austria: Bergern	4	r3	r10	0.00	0.87	-	Kindler *et al*. (2013)
**NHMW 38541**	Austria: Lower Austria: Ernstbrunn	4	r4	r34	0.00	0.98	0.09	This study
**NHMW 36728**	Austria: Lower Austria: Grünbach am Schneeberg	4	r4	r34	0.00	0.98	0.37	This study
**ZFMK 83922**	Austria: Lower Austria: Losenheim	4	r9	r34	0.00	0.91	-	Kindler *et al*. (2013)
**NHMW 36405 (3)**	Austria: Lower Austria: NE Drösing	4	r4	r34	0.02	0.97	0.13	This study
**NHMW 36695 (1)**	Austria: Lower Austria: near Hollabrunn	4	r3	r10	0.00	0.99	0.17	This study
**NHMW 36695 (2)**	Austria: Lower Austria: near Hollabrunn	4	r3	r10	0.00	0.86	-	This study
**NHMW 36405 (2)**	Austria: Lower Austria: Oberedlitz	4	r4	r34	0.00	0.98	0.06	This study
**NHMW 40084 (2)**	Austria: Lower Austria: Orth an der Donau	4	r3	r10	0.00	0.89	-	This study
**NHMW 38557 (1)**	Austria: Lower Austria: Perchtoldsdorf	4	r4	r52	0.00	0.83	-	This study
**NHMW 39496**	Austria: Lower Austria: Petronell-Carnuntum	4	r3	r10	-	-	-	This study
**NHMW 39436 (1)**	Austria: Lower Austria: Sommerein	4	r4	r34	0.00	0.85	-	This study
**NHMW 37311**	Austria: Lower Austria: St. Pölten	4	r3	r10	0.03	0.96	0.10	This study
**NHMW 38810**	Austria: Lower Austria: St. Valentin	4	r3	r10	0.00	0.98	0.10	This study
**NHMW 40084 (1)**	Austria: Lower Austria: Stopfenreuth	4	r3	r3	0.00	0.97	0.08	This study
**ZFMK 73648**	Austria: Styria: Admont/Gesäuse	3	-	y5	0.00	0.96	0.06	Kindler *et al*. (2013)
**NHMW 36994**	Austria: Styria: Eibiswald	4	r4	r34	0.00	0.74	-	This study
**MTD T 14544**	Austria: Styria: Graz	4	r4	-	-	-	-	This study
**MTD T 13539**	Austria: Styria: Seewiesen	4	r4	r41	0.02	0.60	-	This study
**NHMW 39266 (3)**	Austria: Upper Austria: between Stadl Paura and Stadl Traun	4	r4	r35	0.00	0.84	-	This study
**NHMW 38946**	Austria: Upper Austria: Mauthausen	4	r3	r21	0.00	0.99	0.05	This study
**NHMW 36405 (1)**	Austria: Upper Austria: St. Ulrich bei Steyr	4	r3	r21	0.00	0.96	0.06	This study
**NHMW 37422**	Austria: Upper Austria: St. Ulrich bei Steyr	4	r3	r10	0.00	0.98	0.13	This study
**NHMW 38038**	Austria: Upper Austria: St. Ulrich bei Steyr	4	r3	r10	0.00	0.28	-	This study
**NHMW 38599**	Austria: Upper Austria: St. Ulrich bei Steyr	3	y1	y5	0.00	0.99	0.06	This study
**NHMW 39122**	Austria: Upper Austria: St. Ulrich bei Steyr	3	y1	y5	0.02	0.95	0.12	This study
**NHMW 40024 (2)**	Austria: Upper Austria: St. Ulrich bei Steyr	4	r3	r10	0.00	0.80	-	This study
**NHMW 40036 (1)**	Austria: Upper Austria: St. Ulrich bei Steyr	3	y1	y5	0.00	0.83	-	This study
**NHMW 40036 (2)**	Austria: Upper Austria: St. Ulrich bei Steyr	4	r3	r10	0.00	0.99	0.08	This study
**MTD T 14561**	Austria: Vienna	4	r3	r12	0.00	0.99	0.09	This study
**MTD T 14562**	Austria: Vienna	4	r3	r10	0.00	0.96	0.04	This study
**MTD T 9903**	Austria: Vienna: Danube Island	4	r3	r26	0.00	0.94	-	Kindler *et al*. (2013)
**MTD T 9904**	Austria: Vienna: Danube Island	4	r19	r34	0.00	0.80	-	Kindler *et al*. (2013)
**MTD T 9905**	Austria: Vienna: Danube Island	4	r3	r10	0.00	0.96	0.16	Kindler *et al*. (2013)
**NHMW 36284**	Austria: Vienna: Danube Island	4	r3	-	-	-	-	This study
**NHMW 36359**	Austria: Vienna: Danube Island	4	r3	r10	0.00	0.94	-	This study
**NHMW 38557 (2)**	Austria: Vienna: Hadersdorf-Weidlingau	4	r3	r10	0.00	0.96	0.08	This study
**MTD T 8976**	Bosnia and Herzegovina: Magljč Mountain: Prijevor	4	r7	r32	0.01	0.39	-	Kindler *et al*. (2013)
**MTD T 12836**	Bosnia and Herzegovina: near Capljina: Hutovo Blato	4	r4	r34	0.01	0.13	-	This study
**MTD T 12837**	Bosnia and Herzegovina: near Capljina: Hutovo Blato	5	l8	-	0.00	0.02	-	This study
**MTD T 8644**	Bosnia and Herzegovina: near Capljina: Hutovo Blato	5	l2	l12	0.00	0.03	-	Kindler *et al*. (2013)
**MTD T 8645**	Bosnia and Herzegovina: near Capljina: Hutovo Blato	5	l2	l12	0.01	0.05	-	Kindler *et al*. (2013)
**MTD D 40692**	Bulgaria: Albena	3	-	HF680126	0.01	0.23	-	Kindler *et al*. (2013)
**MTD T 10951**	Bulgaria: Arkutino	7	gy1	gy7	0.01	0.07	-	This study
**ZFMK 59679**	Bulgaria: Arkutino	7	gy1	-	0.00	0.07	-	Kindler *et al*. (2013)
**MTD T 10939**	Bulgaria: Asenovgrad: Asenovitza river	7	gy1	gy4	0.01	0.04	-	This study
**MTD T 10953**	Bulgaria: between Pismenovo and Primorsko	7	gy6	-	0.00	0.05	-	This study
**MTD T 10954**	Bulgaria: between Pismenovo and Primorsko	7	gy1	gy10	0.01	0.02	-	This study
**MTD T 8653**	Bulgaria: Bezhanovo	4	r3	r3	0.01	0.31	-	Kindler *et al*. (2013)
**MTD T 10952**	Bulgaria: Bosna pass	7	gy10	gy2	0.01	0.02	-	This study
**MTD T 10948**	Bulgaria: Brashlyan	7	gy2	gy4	0.00	0.10	-	This study
**MTD T 10949**	Bulgaria: Brashlyan	7	gy10	gy2	0.00	0.14	-	This study
**MTD T 8654**	Bulgaria: Etropole	4	r4	r34	0.02	0.26	-	Kindler *et al*. (2013)
**MTD D 18923**	Bulgaria: Levunovo	3	y20	y30	0.01	0.45	-	Kindler *et al*. (2013)

• Table S1 continued

		mtDNA data			Microsatellite data			
**Voucher**	**Locality**	**mtDNA clade**	**ND4 haplotype**	***cyt b* haplotype**	**First STRUCTURE run**	**Second STRUCTURE run**	**Third STRUCTURE run**	**Reference**
**MTD D 18924**	Bulgaria: Levunovo	3	y15	y10	0.00	0.08	-	Kindler *et al*. (2013)
**MTD T 10940**	Bulgaria: Liljanovo	3	y20	y29	0.00	0.55	-	This study
**MTD T 14446**	Bulgaria: Lozenets	7	gy1	gy4	0.01	0.02	-	This study
**MTD T 9321**	Bulgaria: Malko Gradište (pass)	7	gy1	gy4	0.00	0.53	-	Kindler *et al*. (2013)
**MTD D 29984**	Bulgaria: Mičurin	4	r4	r34	-	-	-	Kindler *et al*. (2013)
**BEV.T4054**	Bulgaria: Novo selo	3	y15	y10	0.01	0.29	-	This study
**MTD T 9910**	Bulgaria: Novo Selo	3	-	y10	0.01	0.29	-	Kindler *et al*. (2013)
**MTD T 10946**	Bulgaria: Pass to Slivarovo	7	gy10	gy2	0.01	0.05	-	This study
**MTD T 9012**	Bulgaria: Pejo Javorov	3	y18	y30	0.02	0.36	-	Kindler *et al*. (2013)
**MTD T 9013**	Bulgaria: Pejo Javorov	3	y20	y29	0.00	0.03	-	Kindler *et al*. (2013)
**MTD T 12965**	Bulgaria: Pomorie	7	gy4	gy11	0.00	0.03	-	This study
**MTD T 10941**	Bulgaria: Primorsko	7	gy5	gy6	0.00	0.31	-	This study
**MTD T 10943**	Bulgaria: Primorsko	7	gy1	gy4	0.03	0.04	-	This study
**MTD T 9318**	Bulgaria: Ropotamo	7	gy9	gy2	0.00	0.04	-	Kindler *et al*. (2013)
**MTD T 9319**	Bulgaria: Ropotamo	7	gy5	gy6	0.11	-	-	Kindler *et al*. (2013)
**MTD T 10942**	Bulgaria: Ropotamo	7	gy9	gy2	0.01	0.12	-	This study
**MTD D 19566**	Bulgaria: Sandanksi	3	y20	y30	0.01	0.27	-	Kindler *et al*. (2013)
**MTD D 20867**	Bulgaria: Sandanska Bistrica Valley	3	HF679836	HF680138	0.01	0.52	-	Kindler *et al*. (2013)
**MTD T 8649**	Bulgaria: Shipka pass	7	HF679838	gy4	0.01	0.47	-	Kindler *et al*. (2013)
**MTD T 14453**	Bulgaria: Svištov	4	r4	r61	0.01	0.43	-	This study
**MTD T 14454**	Bulgaria: Svištov	4	r4	r34	0.00	0.96	0.31	This study
**MTD T 10950**	Bulgaria: Zvezdets	7	gy3	gy5	0.01	0.05	-	This study
**ZFMK 54705**	Croatia: 25 km N Rijeka: Gomance	4	r18	-	0.00	0.05	-	Kindler *et al*. (2013)
**ZFMK 92199**	Croatia: Benkovac	4	r4	r34	0.01	0.21	-	Kindler *et al*. (2013)
**ZFMK 91558**	Croatia: Istria: NE Lupoglav	4	r17	r60	0.01	0.10	-	Kindler *et al*. (2013)
**MTD D 32031**	Croatia: Istria: Rovinj	4	r4	r34	0.02	0.02	-	Kindler *et al*. (2013)
**MTD D 32032**	Croatia: Istria: Rovinj	4	r4	r34	0.00	0.05	-	Kindler *et al*. (2013)
**MTD D 20790**	Croatia: Krk	4	r16	HF679930	0.00	0.06	-	Kindler *et al*. (2013)
**MTD T 11596**	Croatia: Krk	4	r16	r34	0.01	0.21	-	This study
**MTD T 11597**	Croatia: Krk	4	r16	r34	0.00	0.43	-	This study
**MTD T 13249**	Croatia: Krk	4	r16	r34	0.01	0.02	-	This study
**MTD T 13250**	Croatia: Krk	4	r16	r34	0.00	0.04	-	This study
**MTD T 13251**	Croatia: Krk	4	r18	r62	0.01	0.08	-	This study
**ZFMK 49094**	Croatia: Krk: N Krk	4	r16	-	0.00	0.04	-	Kindler *et al*. (2013)
**ZFMK 54706**	Croatia: Krk: Punat	4	r16	-	0.00	0.08	-	Kindler *et al*. (2013)
**ZFMK 54709**	Croatia: near Šibenik: Krka falls	4	r11	-	0.00	0.08	-	Kindler *et al*. (2013)
**NHMW 37852**	Croatia: Pag: Vlasici	4	r4	r35	0.00	0.04	-	This study
**ZFMK 54707**	Croatia: Plitvice	4	r16	r34	-	-	-	Kindler *et al*. (2013)
**MTD T 11112**	Croatia: Rab: Lopar	4	r4	r34	0.00	0.02	-	This study
**MTD T 11211**	Croatia: Rab: Lopar	4	r4	r34	0.00	0.04	-	This study
**BEV.1242**	Croatia: southern edge of the Neretva Delta	5	l15	-	0.01	0.02	-	This study
**MTD T 9873**	Croatia: Vransko Jezero	4	r16	-	0.00	0.02	-	Kindler *et al*. (2013)
**ZFMK 76296**	Cyprus: Larnaka: Paralimni	7	gy11	-	0.01	0.01	-	Kindler *et al*. (2013)
**MTD T 11876**	Cyprus: Xyliatos dam	7	gy11	-	0.01	0.01	-	This study
**MTD T 11908**	Cyprus: Xyliatos dam	7	gy11	gy3	0.00	0.01	-	This study
**MTD T 11909**	Cyprus: Xyliatos dam	7	gy11	gy3	0.00	0.01	-	This study
**MTD T 11910**	Cyprus: Xyliatos dam	7	gy11	gy3	0.01	0.01	-	This study
**MTD T 11911**	Cyprus: Xyliatos dam	7	gy11	gy3	0.01	0.01	-	This study
**MTD T 11912**	Cyprus: Xyliatos dam	7	gy6	gy9	0.01	0.03	-	This study
**MTD T 11913**	Cyprus: Xyliatos dam	7	gy6	gy9	0.00	0.03	-	This study
**MTD T 11914**	Cyprus: Xyliatos dam	7	gy6	gy9	0.00	0.04	-	This study
**MTD T 11915**	Cyprus: Xyliatos dam	7	gy6	gy9	0.01	0.02	-	This study
**MTD T 11916**	Cyprus: Xyliatos dam	7	gy6	gy9	0.00	0.08	-	This study
**MTD T 11917**	Cyprus: Xyliatos dam	7	gy6	gy9	0.00	0.09	-	This study
**MTD T 11982**	Cyprus: Xyliatos dam	7	gy11	gy3	0.00	0.01	-	This study
**MTD T 11984**	Cyprus: Xyliatos dam	7	gy11	-	0.01	0.01	-	This study
**MTD T 11985**	Cyprus: Xyliatos dam	7	-	gy3	0.01	0.01	-	This study
**MTD T 12084**	Cyprus: Xyliatos dam	7	-	gy3	0.01	0.01	-	This study
**MTD T 12085**	Cyprus: Xyliatos dam	7	gy11	gy3	0.01	0.01	-	This study
**MTD T 12087**	Cyprus: Xyliatos dam	7	gy11	gy3	0.01	0.01	-	This study
**MTD T 12089**	Cyprus: Xyliatos dam	7	gy11	gy3	0.01	0.01	-	This study
**MTD T 12090**	Cyprus: Xyliatos dam	7	gy11	gy3	0.01	0.01	-	This study
**MTD T 12094**	Cyprus: Xyliatos dam	7	gy11	gy3	0.01	0.01	-	This study
**MTD T 12095**	Cyprus: Xyliatos dam	7	gy11	gy3	0.01	0.01	-	This study
**MTD T 12096**	Cyprus: Xyliatos dam	7	gy11	gy3	0.01	0.01	-	This study
**MTD T 14830**	Cyprus: Xyliatos dam	7	gy11	gy3	0.01	0.01	-	This study
**ZFMK 54318**	Cyprus: Xyliatos dam	7	-	gy3	0.01	0.01	-	Kindler *et al*. (2013)
**ZFMK 61144**	Cyprus: Xyliatos dam	7	-	gy3	0.01	0.01	-	Kindler *et al*. (2013)
**ZFMK 66524**	Cyprus: Xyliatos dam	7	-	gy3	0.01	0.01	-	Kindler *et al*. (2013)
**MTD T 9888**	Czech Republic: Bartošovice	4	r1	-	0.00	0.61	-	Kindler *et al*. (2013)
**MTD T 9878**	Czech Republic: Běstvina	4	r3	-	0.01	0.94	-	Kindler *et al*. (2013)
**MTD T 9579**	Czech Republic: between Horušice and Ruda	4	r3	r10	0.00	0.77	-	Kindler *et al*. (2013)
**MTD T 9580**	Czech Republic: between Horušice and Ruda	4	r4	r34	0.00	0.94	-	Kindler *et al*. (2013)
**MTD T 12995**	Czech Republic: Čepice	4	r4	r34	0.00	0.98	0.22	This study
**MTD T 8963**	Czech Republic: České Budějovice	4	r33	r3	0.00	0.98	0.19	Kindler *et al*. (2013)
**MTD T 9886**	Czech Republic: Chřiby Mountains: Halenkovice	4	r4	r34	0.00	0.93	-	Kindler *et al*. (2013)
**MTD T 9880**	Czech Republic: Chřiby Mountains: Jankovice	4	r4	-	0.00	0.95	0.09	Kindler *et al*. (2013)
**MTD T 9885**	Czech Republic: Chřiby Mountains: Jankovice	4	-	r34	0.00	0.70	-	Kindler *et al*. (2013)
**MTD T 9882**	Czech Republic: Chřiby Mountains: Kudlovice	4	r4	-	0.00	0.97	0.16	Kindler *et al*. (2013)
**MTD T 8974**	Czech Republic: Chřiby Mountains: Kudlovická dolina	4	r32	r3	0.00	0.61	-	Kindler *et al*. (2013)
**MTD T 9879**	Czech Republic: Chřiby Mountains: Salaš	4	r4	-	0.00	0.92	-	Kindler *et al*. (2013)
**MTD T 13009**	Czech Republic: Dlouhá Loučka	4	r4	r34	-	-	-	This study
**MTD T 8636**	Czech Republic: Dolní Lištná	4	r3	-	-	-	-	Kindler *et al*. (2013)
**MTD T 8642**	Czech Republic: Dolní Lištná	4	r3	r10	0.00	0.97	0.64	Kindler *et al*. (2013)
**MTD T 10626**	Czech Republic: Dolní Marklovice;	4	r4	r34	0.01	0.97	0.37	This study
**MTD T 14862**	Czech Republic: Dražovice	4	r4	r34	0.00	0.99	0.20	This study
**MTD T 12781**	Czech Republic: Fojtka	4	r3	r16	0.00	0.99	0.50	This study
**MTD T 8962**	Czech Republic: Heřmanovice	4	r4	r34	0.00	0.97	0.22	Kindler *et al*. (2013)
**MTD T 8964**	Czech Republic: Heřmanovice	4	r4	r34	0.00	0.98	0.26	Kindler *et al*. (2013)
**MTD T 8972**	Czech Republic: Kamýk: Litoměřice	4	r3	r10	0.00	0.98	0.17	Kindler *et al*. (2013)
**MTD T 8634**	Czech Republic: Karviná: Olšiny	4	r4	r34	0.00	0.55	-	Kindler *et al*. (2013)
**MTD T 14863**	Czech Republic: Klikov	4	r4	r34	0.00	0.99	0.14	This study
**MTD T 8966**	Czech Republic: Kokořín	4	r3	r10	0.00	0.99	0.35	Kindler *et al*. (2013)
**MTD T 8965**	Czech Republic: Kokořínský Důl	4	r4	r34	0.00	0.98	0.77	Kindler *et al*. (2013)
**MTD T 8971**	Czech Republic: Kundratice: Litoměřice	4	r3	r10	0.00	0.99	0.60	Kindler *et al*. (2013)
**MTD T 13549**	Czech Republic: Lužnice	4	r3	r10	0.00	0.95	0.26	This study
**MTD T 9881**	Czech Republic: Moravský Písek	4	r4	-	0.00	0.97	0.20	Kindler *et al*. (2013)
**MTD T 14451**	Czech Republic: Na Plachtě, Hradec Králové	4	r3	-	0.00	0.96	0.44	This study
**MTD T 14756**	Czech Republic: Narodní park Podyjí	4	r4	r34	0.00	0.97	0.09	This study
**MTD T 13010**	Czech Republic: Olomouc: Černovír	4	r8	r34	0.00	0.96	0.33	This study
**MTD T 13007**	Czech Republic: Plinkout	4	r4	r58	0.00	0.96	0.31	This study
**MTD T 14755**	Czech Republic: Pouzdřanský rybník	4	r4	r34	0.00	0.96	0.09	This study
**MTD T 14861**	Czech Republic: Sadská	4	r3	r10	0.00	0.99	0.57	This study
**MTD T 8643**	Czech Republic: Staré Hamry	4	r3	r10	0.00	0.98	0.13	Kindler *et al*. (2013)
**MTD T 14769**	Czech Republic: Staré Město nad Metují	4	r2	r10	0.00	0.99	0.72	This study
**MTD T 12794**	Czech Republic: Stráž nad Ohří	3	y1	-	-	-	-	This study
**MTD T 13008**	Czech Republic: Vlárský průsmyk	4	r3	r22	-	-	-	This study
**MTD T 8969**	Czech Republic: Vysoká: Jihlava	4	r3	r9	0.00	0.97	0.12	Kindler *et al*. (2013)
**MTD T 8970**	Czech Republic: Vysoká: Jihlava	4	r3	r10	0.00	0.97	0.14	Kindler *et al*. (2013)
**MTD T 8961**	Czech Republic: Zlaté Hory	4	r2	r10	0.00	0.97	0.27	Kindler *et al*. (2013)
**MTD T 9652**	Denmark: Funen: NNW Svendborg	3	y1	y1	0.01	0.99	0.95	Kindler *et al*. (2013)
**MTD T 9653**	Denmark: Funen: NNW Svendborg	3	y1	y1	0.03	0.98	0.77	Kindler *et al*. (2013)
**MTD T 9654**	Denmark: Funen: NNW Svendborg	3	y1	y1	0.01	0.98	0.80	Kindler *et al*. (2013)
**MTD T 9655**	Denmark: Funen: NNW Svendborg	3	y1	y1	0.00	0.99	0.81	Kindler *et al*. (2013)
**MTD T 9913**	Denmark: Jutland: SE Gammel Rye	3	y1	y38	0.02	0.98	0.95	Kindler *et al*. (2013)
**MTD T 9914**	Denmark: Jutland: SE Gammel Rye	3	y1	y1	0.18	-	-	Kindler *et al*. (2013)
**MTD T 9915**	Denmark: Jutland: SE Gammel Rye	3	y1	y1	0.05	0.98	0.95	Kindler *et al*. (2013)
**MTD T 9916**	Denmark: Jutland: SE Skanderborg	3	y1	y1	0.10	-	-	Kindler *et al*. (2013)
**MTD T 14559**	Denmark: Langeland: Hov	3	y1	y37	0.01	0.99	0.95	This study
**MTD T 9651**	Denmark: Langeland: S Tranekær	3	y1	y17	0.01	0.97	0.93	Kindler *et al*. (2013)
**MTD T 10918**	Denmark: Zealand: 6 km W Koge	3	y30	y1	0.01	0.99	0.92	Kindler *et al*. (2014)
**ZMH R09201**	Denmark: Zealand: Brønsholm	3	y20	-	0.00	0.99	0.94	This study
**ZMH R09021**	Denmark: Zealand: Fredensborg	3	y20	-	-	-	-	This study
**ZMH R09022**	Denmark: Zealand: Fredensborg	3	y20	-	-	-	-	This study

• **Table S1 continued**

		mtDNA data			Microsatellite data			
**Voucher**	**Locality**	**mtDNA clade**	**ND4 haplotype**	**cyt *b* haplotype**	**First STRUCTURE run**	**Second STRUCTURE run**	**Third STRUCTURE run**	**Reference**
**ZMH R09204**	Denmark: Zealand: Fredensborg	**3**	**y20**	-	0.04	0.98	0.95	This study
**ZMH R09205**	Denmark: Zealand: Fredensborg	**3**	**y20**	-	-	-	-	This study
**ZMH R09206**	Denmark: Zealand: Fredensborg	**3**	**y20**	-	-	-	-	This study
**ZMH R09210**	Denmark: Zealand: Fredensborg	**3**	**y20**	-	-	-	-	This study
**ZMH R09211**	Denmark: Zealand: Fredensborg	**3**	**y20**	-	-	-	-	This study
**ZMH R09020**	Denmark: Zealand: Gribskov forest	**3**	**y20**	-	-	-	-	This study
**ZMH R09030**	Denmark: Zealand: Gribskov forest	**3**	**y20**	-	0.00	0.36	-	This study
**ZMH R09055**	Denmark: Zealand: Gribskov forest	**3**	**y20**	-	-	-	-	This study
**MTD T 14546**	Denmark: Zealand: Holte: Vaserne	**3**	**y1**	**y39**	0.00	0.99	0.87	This study
**MTD T 14547**	Denmark: Zealand: Holte: Vaserne	**3**	**y1**	**y40**	0.11	-	-	This study
**MTD T 14548**	Denmark: Zealand: Holte: Vaserne	**3**	**y1**	**y1**	0.00	0.99	0.95	This study
**MTD T 14751**	Denmark: Zealand: N Copenhagen: Jægersborg Dyrehave	**3**	**y1**	**y1**	0.21	-	-	This study
**MTD T 9649**	Denmark: Zealand: N Præstø: Feddet	**3**	**y34**	**y1**	0.01	0.98	0.95	Kindler *et al*. (2013)
**MTD T 9269**	Denmark: Zealand: S Borup	**3**	**y7**	**y1**	0.00	0.99	0.94	Kindler *et al*. (2013)
**MTD T 9270**	Denmark: Zealand: S Borup	**3**	**y7**	**y1**	0.00	0.99	0.95	Kindler *et al*. (2013)
**MTD T 9271**	Denmark: Zealand: S Borup	**3**	**y7**	-	0.00	0.99	0.95	Kindler *et al*. (2013)
**MTD T 14550**	Denmark: Zealand: S Hillerød: Børstingerød Mose	**3**	**y1**	**y40**	0.04	0.99	0.94	This study
**MTD T 14551**	Denmark: Zealand: S Hillerød: Børstingerød Mose	**3**	**y19**	**y1**	0.00	0.99	0.91	This study
**MTD T 14552**	Denmark: Zealand: S Hillerød: Børstingerød Mose	**3**	**y1**	**y1**	0.02	0.99	0.94	This study
**MTD T 14553**	Denmark: Zealand: S Hillerød: Børstingerød Mose	**3**	**y24**	**y1**	0.01	0.99	0.87	This study
**MTD T 14554**	Denmark: Zealand: S Hillerød: Børstingerød Mose	**3**	**y1**	**y40**	0.00	0.99	0.93	This study
**MTD T 14555**	Denmark: Zealand: S Hillerød: Børstingerød Mose	**3**	**y1**	**y1**	0.00	0.99	0.94	This study
**MTD T 14556**	Denmark: Zealand: S Hillerød: Børstingerød Mose	**3**	**y1**	**y40**	0.02	0.99	0.95	This study
**MTD T 14557**	Denmark: Zealand: S Hillerød: Børstingerød Mose	**3**	**y1**	**y1**	0.04	0.99	0.94	This study
**MTD T 14549**	Denmark: Zealand: S Hillerød: Brødeskov	**3**	**y1**	**y40**	0.01	0.99	0.95	This study
**MTD T 14558**	Denmark: Zealand: S Hillerød: near Lillerød: Ravnsholt	**3**	**y1**	**y1**	0.00	0.99	0.89	This study
**MTD T 14341**	Denmark: Zealand: W Køge	**3**	**y1**	**y1**	0.00	0.99	0.94	This study
**MTD T 14342**	Denmark: Zealand: W Køge	**3**	**y1**	**y1**	0.00	0.99	0.91	This study
**MTD T 9648**	Denmark: Zealand: W Køge	**3**	**y1**	**y1**	0.06	-	-	Kindler *et al*. (2013)
**MTD T 11583**	Finland: Åland	**3**	**y1**	**y25**	0.01	0.55	-	Kindler *et al*. (2014)
**MTD T 11584**	Finland: Åland	**3**	**y1**	**y5**	0.00	0.99	0.81	Kindler *et al*. (2014)
**MTD T 11586**	Finland: Åland	**3**	**y5**	**y5**	0.01	0.80	-	Kindler *et al*. (2014)
**MTD T 11589**	Finland: Åland: Finström	**3**	**y1**	**y5**	0.00	0.99	0.93	Kindler *et al*. (2014)
**MTD T 11587**	Finland: Åland: Lemland: Bergö	**3**	**y5**	**y5**	0.01	0.41	-	Kindler *et al*. (2014)
**MTD T 11588**	Finland: Åland: Lemland: Nåtö	**3**	**y1**	**y5**	0.03	0.69	-	Kindler *et al*. (2014)
**MTD T 11591**	Finland: Eastern Finland: Savonlinna: Hernemäki	**8**	**gn1**	**gn4**	0.00	0.02	-	Kindler *et al*. (2014)
**MTD T 11585**	Finland: Southern Finland: Hanko	**8**	**gn6**	**gn11**	0.00	0.05	-	Kindler *et al*. (2014)
**MTD T 11590**	Finland: Western Finland: near Kaarina	**8**	**gn1**	**gn5**	0.00	0.15	-	Kindler *et al*. (2014)
**MTD T 12985**	Former Yugoslav Republic of Macedonia: Crkvino	**3**	**y23**	**y29**	0.01	0.05	-	This study
**MTD T 12986**	Former Yugoslav Republic of Macedonia: Dojran Lake: Mrdaja	**3**	**y15**	-	0.01	0.25	-	This study
**MTD T 12987**	Former Yugoslav Republic of Macedonia: Dojran Lake: Mrdaja	**3**	**y17**	**y10**	0.05	0.15	-	This study
**MTD T 12988**	Former Yugoslav Republic of Macedonia: Dojran Lake: Mrdaja	**3**	**y21**	-	0.17	-	-	This study
**MTD T 12989**	Former Yugoslav Republic of Macedonia: Dojran Lake: Mrdaja	**3**	**y15**	**y11**	0.00	0.30	-	This study
**MTD T 12994**	Former Yugoslav Republic of Macedonia: Dolna Matka	**5**	**l2**	**l10**	0.00	0.41	-	This study
**BEV.T4055**	Former Yugoslav Republic of Macedonia: Jakubica Mountains	**3**	**y15**	**y10**	0.10	-	-	This study
**MTD T 9911**	Former Yugoslav Republic of Macedonia: Jakubica Mountains	**3**	**y15**	**y10**	0.10	-	-	Kindler *et al*. (2013)
**MTD T 12992**	Former Yugoslav Republic of Macedonia: Lake Prespa: Asamati	**5**	**l11**	**l9**	0.03	0.04	-	This study
**MTD T 12993**	Former Yugoslav Republic of Macedonia: Lake Prespa: Pokrvenik	**3**	**y12**	-	0.00	0.28	-	This study
**MTD T 12991**	Former Yugoslav Republic of Macedonia: Lake Prespa: Pretor	**5**	**l2**	-	0.00	0.17	-	This study
**MTD T 13000**	Former Yugoslav Republic of Macedonia: Lake Prespa: Stenje	**5**	**l11**	**l10**	0.15	-	-	This study
**MTD T 9877**	Former Yugoslav Republic of Macedonia: National Park Galičica	**5**	**l12**	**l22**	0.01	0.57	-	Kindler *et al*. (2013)
**MTD T 14447**	Former Yugoslav Republic of Macedonia: Nov Dojran	**3**	**y15**	**y10**	0.02	0.07	-	This study
**MTD T 9909**	Former Yugoslav Republic of Macedonia: Porodin	**5**	**l10**	**l10**	0.03	0.08	-	Kindler *et al*. (2013)
**MTD T 12990**	Former Yugoslav Republic of Macedonia: Star Dojran	**3**	**y15**	**y10**	0.12	-	-	This study
**MTD T 8633**	Former Yugoslav Republic of Macedonia: Stenje	**5**	**l2**	**l10**	0.06	-	-	Kindler *et al*. (2013)
**BEV.1690**	France: Alpes-de-Haute-Provence: Clumanc	**E**	**h7**	-	0.99	-	-	Pokrant *et al*. (2016)
**ZFMK 54711**	France: Ardèche: 10 km N Tournon-sur-Rhône	**E**	**h1**	**h1**	1.00	-	-	Kindler *et al*. (2013)
**BEV.6430**	France: Aveyron: Peyrusse-le-Roc	**E**	**h1**	-	1.00	-	-	Pokrant *et al*. (2016)
**MTD T 10091**	France: Bas-Rhin: Strasbourg	**E**	**h1**	-	1.00	-	-	Kindler *et al*. (2013)
**BEV.11494**	France: Bouches-du-Rhône: Aubagne	**E**	**h1**	-	-	-	-	This study
**BEV.9397**	France: Bouches-du-Rhône: between Fos-sur-Mer and Port-Saint-Louis-du-Rhône	**E**	**h1**	-	1.00	-	-	Pokrant *et al*. (2016)
**ZFMK 54712**	France: Bouches-du-Rhône: Camargue	**E**	**h1**	-	0.98	-	-	Kindler *et al*. (2013)
**BEV.6425**	France: Bouches-du-Rhône: Camargue: Clos de Lange	**E**	**h1**	-	1.00	-	-	Pokrant *et al*. (2016)
**BEV.284**	France: Bouches-du-Rhône: Camargue: Marais du Grenouillet	**E**	**h1**	-	1.00	-	-	Pokrant *et al*. (2016)
**ZMH R07158**	France: Bouches-du-Rhône: Camargue: near Fiélouse	**E**	**h1**	-	-	-	-	This study
**BEV.9398**	France: Bouches-du-Rhône: Camargue: Salin de Badon	**E**	**h1**	-	-	-	-	This study
**ZMH R07572**	France: Bouches-du-Rhône: Camargue: Salin de Badon	**E**	**h1**	**h1**	0.98	-	-	Pokrant *et al*. (2016)
**ZMH R07573**	France: Bouches-du-Rhône: Camargue: Salin de Badon	**E**	**h1**	**h1**	0.99	-	-	Pokrant *et al*. (2016)
**ZMH R06950**	France: Bouches-du-Rhône: Camargue: Tour du Valat	**E**	**h1**	**h1**	0.95	-	-	Pokrant *et al*. (2016)
**ZMH R06951**	France: Bouches-du-Rhône: Camargue: Tour du Valat	**E**	**h1**	**h1**	1.00	-	-	Pokrant *et al*. (2016)
**ZMH R06952**	France: Bouches-du-Rhône: Camargue: Tour du Valat	**E**	**h1**	**h1**	1.00	-	-	Pokrant *et al*. (2016)
**ZMH R06997**	France: Bouches-du-Rhône: Camargue: Tour du Valat	**E**	**h1**	-	0.98	-	-	Pokrant *et al*. (2016)
**ZMH R07062**	France: Bouches-du-Rhône: Camargue: Tour du Valat	**E**	**h1**	**h1**	1.00	-	-	Pokrant *et al*. (2016)
**ZFMK 54710**	France: Bouches-du-Rhône: Saint-Martin-de-Crau	**E**	**h1**	**h1**	0.98	-	-	Kindler *et al*. (2013)
**BEV.9401**	France: Bouches-du-Rhône: Saint-Martin-de-Crau: Mas de Pernes	**E**	**h1**	-	1.00	-	-	Pokrant *et al*. (2016)
**MTD T 11950**	France: Deux-Sèvres: near Niort	**E**	**h1**	**h9**	0.99	-	-	Pokrant *et al*. (2016)
**BEV.11533**	France: Gard: near Alzon: Col de la Barrière	**unknown**	-	-	0.99	-	-	Pokrant *et al*. (2016)
**BEV.9400**	France: Gard: Saint-Hippolyte-du-Fort	**E**	**h12**	-	0.99	-	-	Pokrant *et al*. (2016)
**BEV.T9260**	France: Gironde: near Léognan: Minoy	**E**	**h1**	**h1**	0.97	-	-	Pokrant *et al*. (2016)
**BEV.T9261**	France: Gironde: near Léognan: Minoy	**E**	**h1**	**h1**	1.00	-	-	Pokrant *et al*. (2016)
**BEV.T9264**	France: Hérault: Avène dam	**E**	**h1**	**h27**	0.97	-	-	Pokrant *et al*. (2016)
**BEV.9049**	France: Hérault: between Le Bosc and Loiras	**E**	**h1**	-	1.00	-	-	Pokrant *et al*. (2016)
**BEV.6428**	France: Hérault: Grabels	**unknown**	-	-	1.00	-	-	Pokrant *et al*. (2016)
**BEV.9029**	France: Hérault: Lansargues	**E**	**h1**	-	-	-	-	Pokrant *et al*. (2016)
**BEV.T9266**	France: Hérault: Lattes	**E**	**h1**	**h27**	0.97	-	-	Pokrant *et al*. (2016)
**BEV.T9258**	France: Hérault: Le Mas Blanc	**E**	**h4**	**h1**	0.99	-	-	Pokrant *et al*. (2016)
**BEV.T9259**	France: Hérault: Le Mas Blanc	**E**	**h1**	**h1**	-	-	-	Pokrant *et al*. (2016)
**BEV.T9565**	France: Hérault: Les Corrèges	**E**	**h1**	**h1**	0.99	-	-	This study
**BEV.6421**	France: Hérault: Massif du Caroux	**unknown**	-	-	1.00	-	-	Pokrant *et al*. (2016)
**BEV.8499**	France: Hérault: Massif du Caroux	**E**	**h1**	-	0.99	-	-	Pokrant *et al*. (2016)
**BEV.11496**	France: Hérault: Montpellier	**unknown**			0.99	-	-	Pokrant *et al*. (2016)
**BEV.847**	France: Hérault: Saint-Pierre-de-la-Fage	**E**	**h1**	-	0.99	-	-	Pokrant *et al*. (2016)
**BEV.10974**	France: Hérault: Soulagets	**E**	**h1**	**h15**	-	-	-	This study
**BEV.9041**	France: Hérault: Truscas	**unknown**	-	-	0.98	-	-	Pokrant *et al*. (2016)
**BEV.9293**	France: Isère: Clelles	**E**	**h1**	-	1.00	-	-	Pokrant *et al*. (2016)
**BEV.8324**	France: Loire: Sainte-Foy-Saint-Sulpice	**E**	**h1**	-	1.00	-	-	Pokrant *et al*. (2016)
**MTD T 10092**	France: Puy-de-Dôme: near Murol: Monneaux	**E**	**h1**	-	1.00	-	-	Kindler *et al*. (2013)
**MTD T 11999**	France: Pyrénées-Atlantiques: St. Jean de Luz	**E**	**h1**	-	-	-	-	Pokrant *et al*. (2016)
**MTD T 12024**	France: Saône-et-Loire: Anost	**E**	**h1**	-	-	-	-	This study
**ZFMK 61095**	France: Saône-et-Loire: Anost	**E**	**h1**	**h1**	-	-	-	Kindler *et al*. (2013)
**ZFMK 64930**	France: Saône-et-Loire: Charolles	**E**	**h1**	**h14**	-	-	-	Kindler *et al*. (2013)
**MTD D 40712**	France: Var: N La Londe les Maures	**E**	-	**h8**	-	-	-	Kindler *et al*. (2013)
**BEV.11530**	France: Vaucluse: Saint-Saturnin-lès-Apt	**E**	**h1**	-	0.97	-	-	Pokrant *et al*. (2016)
**MTD T 11973**	Georgia: Akhaldaba	**8**	**gn9**	-	0.00	0.03	-	This study
**MTD T 11975**	Georgia: Akhaldaba	**8**	**gn11**	-	0.00	0.01	-	This study
**ZFMK 76546**	Georgia: Batumi	**8**	**gn12**	**gn14**	0.00	0.02	-	Kindler *et al*. (2013)
**ZFMK 73721**	Georgia: Borshomi	**8**	**gn8**	**gn17**	0.00	0.03	-	Kindler *et al*. (2013)
**ZFMK 73722**	Georgia: Borshomi	**8**	-	**gn10**	0.00	0.03	-	Kindler *et al*. (2013)
**MTD T 14770**	Georgia: Kobuleti	**8**	**gn15**	**gn13**	0.01	0.01	-	This study
**MTD T 14771**	Georgia: Kobuleti	**8**	**gn16**	**gn10**	0.00	0.01	-	This study
**MTD T 8960**	Georgia: Kutaisi	**8**	**gn15**	**gn13**	0.00	0.02	-	Kindler *et al*. (2013)
**MTD T 9341**	Georgia: Mtskheta	**8**	**gn16**	**gn10**	0.01	0.02	-	Kindler *et al*. (2013)
**MTD T 9339**	Georgia: N Stepanzminda	**8**	**gn1**	**gn1**	0.01	0.02	-	Kindler *et al*. (2013)
**MTD T 9340**	Georgia: N Stepanzminda	**8**	**gn1**	**gn19**	0.01	0.02	-	Kindler *et al*. (2013)
**SMNS 14510**	Germany: Baden-Württemberg: Bad Waldsee	**3**	**y1**	**y1**	0.02	0.92	-	This study
**SMNS 14508**	Germany: Baden-Württemberg: Bad Wurzach	**3**	**y1**	**y1**	0.01	0.98	0.07	This study
**SMNS 14509**	Germany: Baden-Württemberg: Bad Wurzach	**3**	**y1**	**y1**	0.04	0.71	-	This study
**MTD T 14814**	Germany: Baden-Württemberg: Berau	**3**	**y1**	**y3**	0.34	-	-	This study
**ZFMK 78777**	Germany: Baden-Württemberg: between Leonberg and Gerlingen	**3**	-	**y5**	0.01	0.96	0.13	Kindler *et al*. (2013)
**MTD D 40907**	Germany: Baden-Württemberg: Böhringer See	**3**	**y1**	-	0.20	-	-	Kindler *et al*. (2013)
**MTD T 14531**	Germany: Baden-Württemberg: Bronnbach	**3**	**y1**	**y5**	0.08	-	-	This study
**SMNS 14503**	Germany: Baden-Württemberg: Dettenheim	**E**	**h1**	**h1**	-	-	-	This study
**MTD T 14816**	Germany: Baden-Württemberg: Freudenstadt	**3**	**y1**	**y34**	0.30	-	-	This study
**MTD T 14835**	Germany: Baden-Württemberg: Freudenstadt	**3**	**y1**	-	-	-	-	This study

• Table S1 continued

		mtDNA data			Microsatellite data			
**Voucher**	**Locality**	**mtDNA clade**	**ND4 haplotype**	**cyt *b* haplotype**	**First STRUCTURE run**	**Second STRUCTURE run**	**Third STRUCTURE run**	**Reference**
**MTD T 14858**	Germany: Baden-Württemberg: Gamburg	3	y1	y5	0.01	0.99	0.27	This study
**MTD T 14828**	Germany: Baden-Württemberg: Harpolingen	E	h1	h23	0.99	-	-	This study
**SMNS 14506**	Germany: Baden-Württemberg: Herrenberg	3	y1	y1	-	-	-	This study
**SMNS 14505**	Germany: Baden-Württemberg: Herten	E	h1	h1	0.97	-	-	This study
**SMNS 14512**	Germany: Baden-Württemberg: Kandern	E	h1	h5	1.00	-	-	This study
**MTD T 14874**	Germany: Baden-Württemberg: Kappel-Grafenhausen	E	h1	h1	1.00	-	-	This study
**MTD T 14837**	Germany: Baden-Württemberg: Karlsruhe	3	y1	y1	0.11	-	-	This study
**MTD T 14841**	Germany: Baden-Württemberg: Karlsruhe	E	h1	h6	0.90	-	-	This study
**SMNK-REP 689**	Germany: Baden-Württemberg: Karlsruhe	E	h1	-	-	-	-	This study
**SMNK-REP 910**	Germany: Baden-Württemberg: Karlsruhe	E	h1	-	0.99	-	-	This study
**SMNK-REP 1003**	Germany: Baden-Württemberg: Karlsruhe: between Staffort and Weingarten	E	h1	-	0.96	-	-	This study
**MTD T 14813**	Germany: Baden-Württemberg: Karlsruhe: Oberwaldsee	E	h1	h1	0.99	-	-	This study
**MTD T 14821**	Germany: Baden-Württemberg: Kniebis	E	h1	h10	1.00	-	-	This study
**MTD T 14843**	Germany: Baden-Württemberg: Laufenburg - Grunholz	3	y1	y1	0.73	-	-	This study
**MTD T 14820**	Germany: Baden-Württemberg: Lenzkirch	E	h1	h1	-	-	-	This study
**SMNK-REP 1268**	Germany: Baden-Württemberg: Linkenheim	E	h1	-	1.00	-	-	This study
**MTD T 14815**	Germany: Baden-Württemberg: lower Neckar valley	3	y1	y5	0.09	-	-	This study
**SMNS 12851**	Germany: Baden-Württemberg: Ludwigsburg	3	y1	-	0.08	-	-	This study
**SMNS 12227**	Germany: Baden-Württemberg: Mettnau	3	y37	y1	0.15	-	-	This study
**SMNS 14514**	Germany: Baden-Württemberg: near Gerlingen: Krummbachtal	3	y1	y26	0.03	0.98	0.16	This study
**SMNS 14515**	Germany: Baden-Württemberg: near Gerlingen: Krummbachtal	3	y1	y5	0.15	-	-	This study
**SMNS 14511**	Germany: Baden-Württemberg: near Heilbronn: Reichertshausen	3	y1	-	0.03	0.97	0.10	This study
**SMNS 9092**	Germany: Baden-Württemberg: near Ravensburg	3	y1	y1	-	-	-	This study
**MTD D 39068**	Germany: Baden-Württemberg: Oberweissach	3	y1	y1	0.18	-	-	Kindler *et al.* (2013)
**SMNS 14260**	Germany: Baden-Württemberg: Ohmden-Jesingen	3	y1	-	0.01	0.98	0.25	This study
**MTD T 14818**	Germany: Baden-Württemberg: Ottenhöfen	E	h1	h1	1.00	-	-	This study
**MTD T 14822**	Germany: Baden-Württemberg: Präg	3	y1	-	0.92	-	-	This study
**MTD T 14823**	Germany: Baden-Württemberg: Präg	E	h1	h1	0.92	-	-	This study
**MTD T 14824**	Germany: Baden-Württemberg: Präg	E	h1	h1	0.99	-	-	This study
**MTD T 14825**	Germany: Baden-Württemberg: Präg	E	h1	h1	0.99	-	-	This study
**MTD T 14826**	Germany: Baden-Württemberg: Präg	E	h1	h1	0.99	-	-	This study
**MTD T 14827**	Germany: Baden-Württemberg: Präg	E	h1	h1	1.00	-	-	This study
**MTD T 14525**	Germany: Baden-Württemberg: Riedlingen	3	y1	y2	0.00	0.99	0.13	This study
**MTD T 14526**	Germany: Baden-Württemberg: Riedlingen	3	y1	-	0.00	0.99	0.46	This study
**MTD T 14527**	Germany: Baden-Württemberg: Riedlingen	3	y1	-	0.06	-	-	This study
**MTD T 14528**	Germany: Baden-Württemberg: Riedlingen	3	y1	y2	0.02	0.99	0.21	This study
**MTD T 14529**	Germany: Baden-Württemberg: Riedlingen	3	y1	y2	0.00	0.99	0.29	This study
**MTD T 14829**	Germany: Baden-Württemberg: Schiltach	3	y1	y34	-	-	-	This study
**MTD T 14831**	Germany: Baden-Württemberg: Schiltach	3	y1	y34	0.18	-	-	This study
**MTD T 11994**	Germany: Baden-Württemberg: Schriesheim	3	y1	y5	0.32	-	-	This study
**MTD T 14839**	Germany: Baden-Württemberg: Steinmauern	E	h1	h1	-	-	-	This study
**SMNS 14517**	Germany: Baden-Württemberg: Stuttgart-Botnang	3	y1	y5	0.00	0.99	0.17	This study
**SMNS 14518**	Germany: Baden-Württemberg: Stuttgart-Feuerbach	3	y1	y5	0.24	-	-	This study
**SMNS 14151**	Germany: Baden-Württemberg: Stuttgart-Hohenheim	3	y1	y1	0.01	0.99	0.11	This study
**SMNS 14519**	Germany: Baden-Württemberg: Stuttgart-Hohenheim, Uni Campus, Eiszeitsee	3	y1	y5	0.09	-	-	This study
**SMNS 14085**	Germany: Baden-Württemberg: Stuttgart-Vaihingen	3	y1	y26	0.22	-	-	This study
**MTD T 14833**	Germany: Baden-Württemberg: Sulz-Fischingen	3	y1	y1	0.09	-	-	This study
**MTD T 12038**	Germany: Baden-Württemberg: Tübingen: Wurmlingen	3	y1	y1	0.02	0.98	0.32	This study
**MTD T 14834**	Germany: Baden-Württemberg: Zwiefalten	3	y1	y2	0.04	0.96	0.25	This study
**MTD T 11137**	Germany: Bavaria: 1 km SSW Jachenau	3	y1	y12	0.39	-	-	This study
**ZMH R09037**	Germany: Bavaria: Berchtesgaden	3	y20	-	-	-	-	This study
**MTD T 11141**	Germany: Bavaria: between Katschenreuth and Frankenberg	3	y1	y5	0.00	0.98	0.31	This study
**MTD T 11142**	Germany: Bavaria: between Katschenreuth and Frankenberg	3	y1	y5	0.01	0.98	0.54	This study
**MTD T 11143**	Germany: Bavaria: between Katschenreuth and Frankenberg	3	y1	y5	0.00	0.99	0.79	This study
**MTD T 11145**	Germany: Bavaria: Dreschen	3	y1	y5	0.01	0.98	0.62	This study
**MTD T 14560**	Germany: Bavaria: E Passau: Erlau	4	r3	r10	0.00	0.98	0.30	This study
**MTD T 11147**	Germany: Bavaria: Haunstetten	3	y1	y13	0.00	0.96	0.10	This study
**MTD T 11136**	Germany: Bavaria: Jochenstein	4	r3	r10	0.00	0.93	-	This study
**MTD T 11158**	Germany: Bavaria: Klautzenbach	4	r3	r10	0.00	0.99	0.53	This study
**ZFMK 71166**	Germany: Bavaria: Mammendorf	4	r3	r20	0.00	0.97	0.13	Kindler *et al.* (2013)
**MTD T 11134**	Germany: Bavaria: Munich	4	r3	r10	0.00	0.95	0.10	This study
**MTD T 11149**	Germany: Bavaria: Munich: Feldmoching	4	r3	r10	0.00	0.99	0.28	This study
**MTD T 11150**	Germany: Bavaria: Munich: Obermenzing	4	r3	r10	0.00	0.56	-	This study
**MTD T 11151**	Germany: Bavaria: Munich: Obermenzing	4	r3	r10	0.00	0.99	0.07	This study
**MTD T 11152**	Germany: Bavaria: Munich: Obermenzing	4	r3	r10	0.00	0.98	0.04	This study
**MTD T 11153**	Germany: Bavaria: Munich: Obermenzing	3	y1	y12	0.00	0.91	-	This study
**MTD T 11154**	Germany: Bavaria: Munich: Obermenzing	3	y1	y12	0.00	0.98	0.04	This study
**MTD T 11155**	Germany: Bavaria: Munich: Obermenzing	4	r3	r10	0.00	0.80	-	This study
**MTD T 11133**	Germany: Bavaria: Murnauer Moos	3	y1	y5	0.00	0.99	0.19	This study
**ZSM DNA 171**	Germany: Bavaria: near Bad Aibling	4	r3	r10	0.00	0.99	0.07	This study
**MTD T 11139**	Germany: Bavaria: near Haiming	4	r3	r10	0.00	0.98	0.07	This study
**MTD T 11144**	Germany: Bavaria: near Neuenreuth	3	y1	y5	0.04	0.98	0.83	This study
**MTD T 14352**	Germany: Bavaria: Olching	4	r3	r10	0.00	0.99	0.07	This study
**MTD T 11135**	Germany: Bavaria: Passau	4	r3	r10	0.00	0.99	0.11	This study
**ZFMK 56016**	Germany: Bavaria: Passau	4	r3	r10	0.01	0.94	-	Kindler *et al.* (2013)
**MTD T 11138**	Germany: Bavaria: Schliersee	4	r3	r10	0.02	0.99	0.11	This study
**MTD T 14832**	Germany: Bavaria: Schwandorf	3	y1	y12	0.00	0.99	0.08	This study
**MTD T 11140**	Germany: Bavaria: Wackersdorf	3	y1	-	0.00	0.99	0.34	This study
**MTD T 11148**	Germany: Bavaria: Wolfratshausen	3	y1	y12	0.00	0.99	0.14	This study
**MTD T 14172**	Germany: Berlin: Teufelssee	3	y1	-	0.00	0.93	-	This study
**MTD T 14173**	Germany: Berlin: Teufelssee	3	y1	-	0.00	0.97	0.15	This study
**MTD D 47570**	Germany: Brandenburg: Brieske	3	y1	-	0.00	0.99	0.56	Kindler *et al.* (2013)
**NME R 0492/06**	Germany: Brandenburg: Casel	3	y1	-	-	-	-	This study
**MTD D 29503**	Germany: Brandenburg: Cumlosen	4	r4	HF680017	0.01	0.51	-	Kindler *et al.* (2013)
**MTD T 11460**	Germany: Brandenburg: Forst	3	y1	y6	0.00	0.98	0.41	This study
**MTD T 14362**	Germany: Brandenburg: Golm	4	r3	r10	0.00	0.98	0.60	This study
**MTD T 14363**	Germany: Brandenburg: Golm	3	y1	y17	0.00	0.99	0.84	This study
**MTD T 14364**	Germany: Brandenburg: Golm	3	y1	y5	0.00	0.99	0.92	This study
**MTD T 14365**	Germany: Brandenburg: Golm	3	y35	y33	0.00	0.99	0.67	This study
**MTD T 14366**	Germany: Brandenburg: Golm	3	y1	y5	0.00	0.96	0.88	This study
**MTD T 14367**	Germany: Brandenburg: Golm	3	y1	y5	0.00	0.99	0.89	This study
**MTD T 14369**	Germany: Brandenburg: Golm	3	y1	y5	0.00	0.99	0.92	This study
**MTD T 14370**	Germany: Brandenburg: Golm	3	y1	y5	0.00	0.98	0.78	This study
**MTD T 14371**	Germany: Brandenburg: Golm	3	y1	y5	0.00	0.99	0.83	This study
**MTD T 14372**	Germany: Brandenburg: Golm	4	r3	r15	0.00	0.99	0.54	This study
**MTD T 14373**	Germany: Brandenburg: Golm	3	y35	y33	0.00	0.98	0.66	This study
**MTD T 14374**	Germany: Brandenburg: Golm	3	y35	y33	0.00	0.99	0.93	This study
**MTD T 14375**	Germany: Brandenburg: Golm	3	y1	y5	0.00	0.96	0.68	This study
**MTD T 14376**	Germany: Brandenburg: Golm	3	y1	y5	0.00	0.99	0.80	This study
**MTD T 14377**	Germany: Brandenburg: Golm	3	y1	y5	0.00	0.97	0.87	This study
**MTD T 14378**	Germany: Brandenburg: Golm	4	r3	r10	0.00	0.97	0.90	This study
**MTD T 14379**	Germany: Brandenburg: Golm	3	y1	y5	0.00	0.98	0.56	This study
**MTD T 14380**	Germany: Brandenburg: Golm	3	y1	y5	0.00	0.96	0.78	This study
**MTD T 14381**	Germany: Brandenburg: Golm	3	y1	y5	0.00	0.97	0.87	This study
**MTD T 14382**	Germany: Brandenburg: Golm	3	y1	y5	0.00	0.99	0.89	This study
**MTD T 14383**	Germany: Brandenburg: Golm	3	y1	y5	0.00	0.95	0.96	This study
**MTD T 14384**	Germany: Brandenburg: Golm	3	y1	y5	0.00	0.99	0.86	This study
**MTD T 14385**	Germany: Brandenburg: Golm	3	y1	y5	0.00	0.98	0.83	This study
**MTD T 14386**	Germany: Brandenburg: Golm	3	y1	y5	0.00	0.99	0.92	This study
**MTD T 14387**	Germany: Brandenburg: Golm	3	y1	y5	0.00	0.99	0.91	This study
**MTD T 14530**	Germany: Brandenburg: Gülpe	3	y8	y17	0.00	0.95	0.88	This study
**MTD D 47197**	Germany: Brandenburg: Guteborn	3	y1	y5	0.00	0.97	0.48	Kindler *et al.* (2013)
**MTD T 3813**	Germany: Brandenburg: Guteborn	3	-	y5	0.00	0.97	0.48	Kindler *et al.* (2013)
**MTD D 45304**	Germany: Brandenburg: Hosena	3	y1	y19	0.00	0.98	0.89	Kindler *et al.* (2013)
**ZMB 66570**	Germany: Brandenburg: near Bamme	3	y1	y5	0.00	0.99	0.60	Kindler *et al.* (2014)
**MTD D 29504**	Germany: Brandenburg: Perleberg	3	y20	y29	-	-	-	Kindler *et al.* (2013)
**MTD T 14345**	Germany: Brandenburg: Schwedt/Oder	3	y1	y17	0.00	0.99	0.92	This study
**ZFMK 76358**	Germany: Brandenburg: Senftenberg	3	y1	y17	0.00	0.98	0.89	Kindler *et al.* (2013)
**MTD T 14368**	Germany: Brandenburg: Werder	3	y13	y17	0.00	0.98	0.74	This study
**ZMH R09735**	Germany: Hamburg: Klövensteen	3	y1	y20	0.00	0.99	0.94	This study
**ZMH R06162**	Germany: Hamburg: Ohlstedt	3	y1	-	0.03	0.99	0.95	This study
**ZMH R09009**	Germany: Hamburg: Wohldorf	3	y1	-	-	-	-	This study
**ZMH R09048**	Germany: Hamburg: Wohldorf	3	y20	-	-	-	-	This study
**MTD T 14620**	Germany: Hesse: Bebra-Weiterode	3	y1	y5	0.00	0.98	0.84	This study

• Table S1 continued

		mtDNA data			Microsatellite data			
**Voucher**	**Locality**	**mtDNA clade**	**ND4 haplotype**	**cyt *b* haplotype**	**First STRUCTURE run**	**Second STRUCTURE run**	**Third STRUCTURE run**	**Reference**
**SMF 76440**	Germany: Hesse: Bergen-Enkheim	**E**	**h1**	**h1**	0.91	-	-	This study
**SMF 71026**	Germany: Hesse: Biebergemünd	**3**	**y1**	-	0.01	0.99	0.62	This study
**SMF 71027**	Germany: Hesse: Biebergemünd	**3**	**y1**	**y31**	0.06	-	-	This study
**SMF 77543**	Germany: Hesse: Biebergemünd-Breitenborn	**3**	**y1**	**y5**	0.20	-	-	This study
**SMF 78480**	Germany: Hesse: Flieden	**3**	**y1**	**y31**	0.00	0.98	0.69	This study
**ZFMK 58023**	Germany: Hesse: Flieden	**3**	**y39**	**y5**	0.09	-	-	This study
**ZFMK 82929**	Germany: Hesse: Flieden	**3**	**y1**	**y31**	0.06	-	-	Kindler *et al*. (2013)
**SMF 79183**	Germany: Hesse: Hasselroth-Neuenhaßlau	**3**	**y1**	**y5**	0.39	-	-	This study
**ZFMK 89403**	Germany: Hesse: Herborn	**E**	**h1**	**h1**	-	-	-	Kindler *et al*. (2013)
**MTD T 14112**	Germany: Hesse: Herborn	**E**	**h1**	**h1**	1.00	-	-	This study
**MTD T 14842**	Germany: Hesse: Hirschhorn	**3**	**y1**	**y5**	-	-	-	This study
**MTD D 35776**	Germany: Hesse: Hofheim/Lorsbuch	**E**	**h1**	**h24**	0.97	-	-	Kindler *et al*. (2013)
**MTD T 14175**	Germany: Hesse: Lautertal-Beedenkirchen	**E**	**h1**	-	0.73	-	-	This study
**ZSUM Re.S2.1.2.c**	Germany: Hesse: Marburg	**E**	**h1**	**h1**	0.97	-	-	This study
**ZSUM Re.S2.1.1.a**	Germany: Hesse: Marburg: Lahn mountains	C	**c1**	**c2**	0.33	-	-	This study
**ZSUM Re.S2.1.2.a**	Germany: Hesse: Marburg: Lahn mountains	C	**c1**	-	0.43	-	-	This study
**ZSUM Re.S2.1.2.b**	Germany: Hesse: Marburg: Lahn mountains	**unknown**	-	-	0.47	-	-	This study
**ZSUM Re.S2.1.2.g**	Germany: Hesse: Marburg: Lahn mountains	C	**c1**	-	0.99	-	-	This study
**MTD T 13929**	Germany: Hesse: Marburg: new botanical garden	**3**	**y1**	-	-	-	-	This study
**MTD T 13930**	Germany: Hesse: Marburg: new botanical garden	**3**	**y1**	-	-	-	-	This study
**MTD T 13931**	Germany: Hesse: Marburg: new botanical garden	**3**	**y39**	**y5**	0.01	0.95	0.80	This study
**MTD T 13932**	Germany: Hesse: Marburg: new botanical garden	**3**	**y39**	-	0.03	0.93	-	This study
**MTD T 13933**	Germany: Hesse: Marburg: new botanical garden	**3**	**y39**	**y5**	0.41	-	-	This study
**MTD T 13400**	Germany: Hesse: near Bad Homburg	**3**	**y1**	-	0.00	0.99	0.90	This study
**SMF 73339**	Germany: Hesse: near Bieber	**3**	**y1**	**y31**	0.04	0.96	0.82	This study
**SMF 72196**	Germany: Hesse: near Hanau: Mittelbuchen	**E**	**h1**	-	-	-	-	This study
**SMF 72456**	Germany: Hesse: near Kelkheim: Fischbach	**E**	**h1**	**h24**	0.99	-	-	This study
**ZFMK 82930**	Germany: Hesse: Steinau	**4**	**r3**	**r10**	0.00	0.83	-	Kindler *et al*. (2013)
**SMF 75825**	Germany: Hesse: SW Gross-Gerau: Kühkopf	**E**	**h1**	**h1**	1.00	-	-	This study
**SMF 74531**	Germany: Hesse: W Oberndorf	**3**	**y1**	**y31**	0.07	-	-	This study
**SMF 78703**	Germany: Hesse: Weilrod: Niederlauken	**E**	**h1**	**h4**	0.99	-	-	This study
**MTD T 14749**	Germany: Lower Saxony: Cuxhaven: Spanger Heide	**3**	**y1**	**y17**	0.00	0.99	0.95	This study
**MTD T 14750**	Germany: Lower Saxony: Cuxhaven: Spanger Heide	**3**	**y1**	**y18**	0.00	0.99	0.89	This study
**LMNM REP963**	Germany: Lower Saxony: Frieschenmoor	**3**	**y1**	**y17**	0.05	0.87	-	This study
**MTD T 13919**	Germany: Lower Saxony: Gartow	**3**	**y35**	-	0.00	0.99	0.90	This study
**MTD T 13374**	Germany: Lower Saxony: Hannover	**3**	**y1**	-	-	-	-	This study
**MTD T 13367**	Germany: Lower Saxony: Hannover	**3**	**y1**	**y27**	-	-	-	This study
**LMNM REP961**	Germany: Lower Saxony: Hatterwüsting	**3**	**y1**	**y17**	-	-	-	This study
**LMNM REP107**	Germany: Lower Saxony: Hude	**3**	**y1**	**y17**	0.02	0.99	0.95	This study
**LMNM REP960**	Germany: Lower Saxony: Huntlosen	**3**	**y1**	**y18**	0.16	-	-	This study
**MTD T 13370**	Germany: Lower Saxony: Lachendorf	**3**	**y35**	-	0.01	0.98	0.90	This study
**MTD T 13371**	Germany: Lower Saxony: Lachendorf	**3**	**y1**	-	0.00	0.99	0.95	This study
**ZFMK 86134**	Germany: Lower Saxony: Lachendorf	**3**	**y1**	**y5**	0.00	0.99	0.92	Kindler *et al*. (2013)
**ZFMK 89088**	Germany: Lower Saxony: Leiferde	**3**	**y1**	**y9**	0.01	0.99	0.71	Kindler *et al*. (2013)
**ZMH R06756**	Germany: Lower Saxony: Lüchow-Dannenberg: Pevestorf	**3**	**y20**	**y29**	0.00	0.65	-	This study
**ZMH R06758**	Germany: Lower Saxony: Lüchow-Dannenberg: Pevestorf	**3**	**y20**	**y29**	0.12	-	-	This study
**ZMH R06945**	Germany: Lower Saxony: Lüchow-Dannenberg: Pevestorf	**3**	**y20**	**y29**	0.00	0.93	-	This study
**ZMH R06959**	Germany: Lower Saxony: Lüchow-Dannenberg: Pevestorf	**3**	**y20**	-	0.01	0.46	-	This study
**ZMH R07057**	Germany: Lower Saxony: Lüchow-Dannenberg: Pevestorf	**3**	**y20**	**y29**	0.01	0.78	-	This study
**ZMH R07878**	Germany: Lower Saxony: Lüchow-Dannenberg: Pevestorf	**3**	**y20**	**y29**	0.00	0.73	-	This study
**ZMH R08415**	Germany: Lower Saxony: Lüchow-Dannenberg: Pevestorf	**3**	**y20**	**y29**	0.00	0.08	-	This study
**ZMH R08936**	Germany: Lower Saxony: Lüchow-Dannenberg: Pevestorf	**3**	**y20**	**y29**	0.00	0.39	-	This study
**ZMH R09335**	Germany: Lower Saxony: Lüchow-Dannenberg: Pevestorf	**3**	**y20**	**y29**	0.00	0.40	-	This study
**ZMH R09336**	Germany: Lower Saxony: Lüchow-Dannenberg: Pevestorf	**3**	**y20**	-	0.00	0.92	-	This study
**ZMH R09337**	Germany: Lower Saxony: Lüchow-Dannenberg: Pevestorf	**3**	**y20**	**y29**	0.00	0.58	-	This study
**ZMH R09540**	Germany: Lower Saxony: Lüchow-Dannenberg: Pevestorf	**3**	**y20**	**y29**	0.01	0.85	-	This study
**ZMH R09636**	Germany: Lower Saxony: Lüchow-Dannenberg: Pevestorf	**3**	**y20**	**y29**	0.00	0.92	-	This study
**ZMH R09742**	Germany: Lower Saxony: Lüchow-Dannenberg: Pevestorf	**3**	**y20**	**y29**	0.00	0.66	-	This study
**ZMH R09754**	Germany: Lower Saxony: Lüchow-Dannenberg: Pevestorf	**3**	**y20**	**y29**	0.00	0.85	-	This study
**MTD T 13373**	Germany: Lower Saxony: Meißendorf	**3**	**y1**	**y17**	0.01	0.98	0.77	This study
**MTD T 13368**	Germany: Lower Saxony: near Celle: Bannetzer Moor	**3**	**y1**	-	0.01	0.99	0.54	This study
**MTD T 13369**	Germany: Lower Saxony: near Cuxhaven: Arensch	**3**	**y1**	-	0.01	0.99	0.55	This study
**MTD T 13372**	Germany: Lower Saxony: near Dannefeld	**3**	**y35**	-	0.00	0.97	0.77	This study
**LMNM REP957**	Germany: Lower Saxony: Oldenbrok	**3**	**y1**	**y17**	0.08	-	-	This study
**LMNM REP496**	Germany: Lower Saxony: Oldenburg	**3**	**y1**	-	-	-	-	This study
**LMNM REP962**	Germany: Lower Saxony: Oldenburg: Eversten	**3**	**y1**	**y17**	0.15	-	-	This study
**MTD T 14621**	Germany: Lower Saxony: Oldendorf (Luhe)	**3**	-	**y19**	-	-	-	This study
**MTD T 14622**	Germany: Lower Saxony: Oldendorf (Luhe)	**3**	**y1**	**y19**	0.00	0.99	0.93	This study
**LMNM REP553**	Germany: Lower Saxony: Rastede	**3**	**y1**	-	-	-	-	This study
**LMNM REP128**	Germany: Lower Saxony: S Oldenburg: Barneführer Holz	**3**	**y1**	-	0.02	0.98	0.42	This study
**ZMH R06717**	Germany: Lower Saxony: Salzhausen	**3**	**y1**	-	-	-	-	This study
**ZMH R09016**	Germany: Lower Saxony: Stade	**3**	**y1**	-	-	-	-	This study
**MTD T 13917**	Germany: Lower Saxony: Steinhuder Meer	**3**	**y1**	**y17**	0.03	0.99	0.91	This study
**MTD T 13918**	Germany: Lower Saxony: Steinhuder Meer	**3**	**y1**	**y17**	0.02	0.99	0.84	This study
**MTD T 11465**	Germany: Mecklenburg-Western Pomerania: 3 km E Wesenberg	**3**	**y1**	**y22**	0.00	0.99	0.94	Kindler *et al*. (2014)
**MTD T 11466**	Germany: Mecklenburg-Western Pomerania: 3 km E Wesenberg	**3**	**y1**	**y17**	0.00	0.98	0.87	Kindler *et al*. (2014)
**MTD T 11467**	Germany: Mecklenburg-Western Pomerania: 3 km E Wesenberg	**3**	**y1**	**y17**	0.00	0.99	0.91	Kindler *et al*. (2014)
**MTD T 11468**	Germany: Mecklenburg-Western Pomerania: 3 km E Wesenberg	**3**	**y31**	**y17**	0.01	0.91	-	Kindler *et al*. (2014)
**MTD T 11469**	Germany: Mecklenburg-Western Pomerania: 3 km E Wesenberg	**3**	**y1**	**y17**	0.00	0.98	0.85	Kindler *et al*. (2014)
**MTD T 11470**	Germany: Mecklenburg-Western Pomerania: 3 km E Wesenberg	**3**	**y1**	**y17**	0.00	0.99	0.87	Kindler *et al*. (2014)
**MTD T 11471**	Germany: Mecklenburg-Western Pomerania: 3 km E Wesenberg	**3**	**y1**	**y17**	0.00	0.98	0.89	Kindler *et al*. (2014)
**MTD T 11472**	Germany: Mecklenburg-Western Pomerania: 3 km E Wesenberg	**3**	**y1**	**y17**	0.00	0.99	0.89	Kindler *et al*. (2014)
**MTD T 11473**	Germany: Mecklenburg-Western Pomerania: 3 km E Wesenberg	**3**	**y1**	**y17**	0.00	0.99	0.84	Kindler *et al*. (2014)
**MTD T 11474**	Germany: Mecklenburg-Western Pomerania: 3 km E Wesenberg	**3**	**y1**	**y17**	0.00	0.98	0.95	Kindler *et al*. (2014)
**MTD T 11476**	Germany: Mecklenburg-Western Pomerania: 3 km E Wesenberg	**3**	**y1**	**y17**	0.00	0.99	0.89	Kindler *et al*. (2014)
**MTD T 11477**	Germany: Mecklenburg-Western Pomerania: 3 km E Wesenberg	**3**	**y1**	**y22**	0.00	0.99	0.90	Kindler *et al*. (2014)
**MTD T 11478**	Germany: Mecklenburg-Western Pomerania: 3 km E Wesenberg	**3**	**y1**	**y17**	0.00	0.98	0.91	Kindler *et al*. (2014)
**MTD T 11479**	Germany: Mecklenburg-Western Pomerania: 3 km E Wesenberg	**3**	**y1**	**y17**	0.00	0.96	0.48	Kindler *et al*. (2014)
**MTD T 11480**	Germany: Mecklenburg-Western Pomerania: 3 km E Wesenberg	**3**	**y1**	**y17**	0.00	0.99	0.94	Kindler *et al*. (2014)
**MTD T 11481**	Germany: Mecklenburg-Western Pomerania: 3 km E Wesenberg	**3**	**y1**	**y17**	0.00	0.99	0.91	Kindler *et al*. (2014)
**MTD T 11482**	Germany: Mecklenburg-Western Pomerania: 3 km E Wesenberg	**3**	**y1**	**y17**	0.00	0.98	0.92	Kindler *et al*. (2014)
**NME R 0567/08**	Germany: Mecklenburg-Western Pomerania: Boitin	**3**	**y1**	**y17**	0.00	0.99	0.93	This study
**NME R 0973/14**	Germany: Mecklenburg-Western Pomerania: Groß-Miltzow	**3**	**y22**	-	0.00	0.97	0.18	This study
**NME R 0974/14**	Germany: Mecklenburg-Western Pomerania: Groß-Miltzow	**3**	**y1**	**y17**	0.00	0.99	0.95	This study
**ZFMK 61035**	Germany: Mecklenburg-Western Pomerania: Güstrow	**3**	-	**y17**	-	-	-	Kindler *et al*. (2013)
**NME R 0414/02**	Germany: Mecklenburg-Western Pomerania: near Ahlbeck	**3**	**y1**	-	0.00	0.99	0.69	This study
**NME R 0963/14**	Germany: Mecklenburg-Western Pomerania: near Ahlbeck	**3**	**y1**	-	0.00	0.98	0.43	This study
**MTD T 12145**	Germany: Mecklenburg-Western Pomerania: near Altwarp	**3**	**y1**	**y17**	0.00	0.97	0.90	This study
**MTD T 12146**	Germany: Mecklenburg-Western Pomerania: near Altwarp	**3**	**y1**	**y17**	0.00	0.98	0.79	This study
**MTD T 12147**	Germany: Mecklenburg-Western Pomerania: near Altwarp	**3**	**y1**	**y17**	0.00	0.98	0.93	This study
**MTD T 12148**	Germany: Mecklenburg-Western Pomerania: near Altwarp	**3**	**y1**	**y17**	0.00	0.97	0.95	This study
**MTD T 12149**	Germany: Mecklenburg-Western Pomerania: near Altwarp	**3**	**y1**	-	0.00	0.98	0.92	This study
**MTD T 12150**	Germany: Mecklenburg-Western Pomerania: near Altwarp	**3**	**y1**	-	0.00	0.98	0.80	This study
**MTD T 12134**	Germany: Mecklenburg-Western Pomerania: near Carwitz	**3**	**y22**	**y17**	0.00	0.99	0.95	This study
**MTD T 12135**	Germany: Mecklenburg-Western Pomerania: near Carwitz	**3**	**y1**	**y17**	0.00	0.99	0.91	This study
**MTD T 12136**	Germany: Mecklenburg-Western Pomerania: near Carwitz	**3**	**y1**	**y17**	0.00	0.99	0.85	This study
**MTD T 12137**	Germany: Mecklenburg-Western Pomerania: near Carwitz	**3**	**y1**	**y17**	0.00	0.99	0.81	This study
**MTD T 12138**	Germany: Mecklenburg-Western Pomerania: near Carwitz	**3**	**y22**	**y17**	0.00	0.99	0.88	This study
**MTD T 12139**	Germany: Mecklenburg-Western Pomerania: near Carwitz	**3**	**y1**	-	0.00	0.99	0.86	This study
**MTD T 12140**	Germany: Mecklenburg-Western Pomerania: near Carwitz	**3**	**y1**	**y17**	0.00	0.90	-	This study
**MTD T 12141**	Germany: Mecklenburg-Western Pomerania: near Carwitz	**3**	**y22**	**y17**	0.00	0.99	0.62	This study
**MTD T 12142**	Germany: Mecklenburg-Western Pomerania: near Carwitz	**3**	**y1**	-	0.00	0.98	0.90	This study
**MTD T 12143**	Germany: Mecklenburg-Western Pomerania: near Carwitz	**3**	**y22**	**y17**	0.01	0.98	0.61	This study
**MTD T 12144**	Germany: Mecklenburg-Western Pomerania: near Carwitz	**3**	**y1**	**y17**	0.00	0.98	0.90	This study
**MTD T 14482**	Germany: Mecklenburg-Western Pomerania: near Stralsund	**3**	**y1**	**y17**	0.00	0.98	0.88	This study
**MTD T 12829**	Germany: Mecklenburg-Western Pomerania: near Wustrow	**3**	**y1**		0.00	0.99	0.40	This study
**MTD T 11462**	Germany: Mecklenburg-Western Pomerania: Neubrandenburg	**3**	**y1**	**y17**	0.00	0.99	0.91	Kindler *et al*. (2014)
**MTD T 11463**	Germany: Mecklenburg-Western Pomerania: Neubrandenburg	**3**	**y1**	**y17**	0.00	0.99	0.92	Kindler *et al*. (2014)
**MTD T 11464**	Germany: Mecklenburg-Western Pomerania: Neubrandenburg	**3**	**y1**	**y17**	0.00	0.99	0.83	Kindler *et al*. (2014)
**NME R 0475/06**	Germany: Mecklenburg-Western Pomerania: Neubrandenburg	**3**	**y1**	**y17**	0.00	0.98	0.88	This study
**MTD T 11475**	Germany: Mecklenburg-Western Pomerania: Neubrandenburg: Lake Tollense	**3**	**y1**	**y17**	0.00	0.98	0.77	Kindler *et al*. (2014)
**ZFMK 47435**	Germany: Mecklenburg-Western Pomerania: Neukloster	**3**	**y1**	-	0.00	0.99	0.95	Kindler *et al*. (2013)
**NME R 0474/06**	Germany: Mecklenburg-Western Pomerania: Prerow	**3**	**y43**	**y1**	0.00	0.99	0.93	This study
**MTD T 14508**	Germany: Mecklenburg-Western Pomerania: Rostock: Radelsee	**3**	**y10**	**y17**	0.00	0.99	0.95	This study

• Table S1 continued

Voucher	Locality	mtDNA data: mtDNA clade	mtDNA data: ND4 haplotype	mtDNA data: cyt *b* haplotype	Microsatellite data: First STRUCTURE run	Microsatellite data: Second STRUCTURE run	Microsatellite data: Third STRUCTURE run	Reference
**MTD T 14483**	Germany: Mecklenburg-Western Pomerania: Saal: Neuendorf-Heide	**3**	**y1**	**y1**	0.00	0.96	0.94	This study
**MTD T 14484**	Germany: Mecklenburg-Western Pomerania: Saal: Neuendorf-Heide	**3**	**y1**	**y1**	0.00	0.98	0.91	This study
**MTD T 11483**	Germany: Mecklenburg-Western Pomerania: Usedom	**3**	**y1**	**y17**	0.00	0.94	-	Kindler *et al*. (2014)
**MTD T 11484**	Germany: Mecklenburg-Western Pomerania: Usedom	**3**	**y1**	**y17**	0.00	0.98	0.90	Kindler *et al*. (2014)
**ZFMK 68431**	Germany: North Rhine-Westphalia: Bad Honnef	**E**	**h1**	-	1.00	-	-	Kindler *et al*. (2013)
**ZFMK 68432**	Germany: North Rhine-Westphalia: Bad Honnef	**E**	**h1**	**h1**	1.00	-	-	Kindler *et al*. (2013)
**ZFMK 92193**	Germany: North Rhine-Westphalia: Bad Münstereifel	**E**	**h1**	**h1**	0.99	-	-	Kindler *et al*. (2013)
**MTD T 11191**	Germany: North Rhine-Westphalia: between Ammelsbüren and Davensberg	**E**	**h1**	**h1**	0.98	-	-	This study
**MTD T 11193**	Germany: North Rhine-Westphalia: between Ammelsbüren and Davensberg	**E**	**h1**	**h1**	1.00	-	-	This study
**MTD T 11194**	Germany: North Rhine-Westphalia: between Ammelsbüren and Davensberg	**E**	**h1**	**h1**	0.99	-	-	This study
**MTD T 11195**	Germany: North Rhine-Westphalia: between Ammelsbüren and Davensberg	**E**	**h1**	**h1**	0.99	-	-	This study
**MTD T 11196**	Germany: North Rhine-Westphalia: between Ammelsbüren and Davensberg	**E**	**h1**	**h1**	0.99	-	-	This study
**MTD T 11197**	Germany: North Rhine-Westphalia: between Ammelsbüren and Davensberg	**E**	**h1**	**h1**	1.00	-	-	This study
**MTD T 11198**	Germany: North Rhine-Westphalia: between Ammelsbüren and Davensberg	**E**	**h1**	**h1**	0.85	-	-	This study
**MTD T 11199**	Germany: North Rhine-Westphalia: between Ammelsbüren and Davensberg	**E**	**h1**	**h1**	1.00	-	-	This study
**MTD T 11200**	Germany: North Rhine-Westphalia: between Ammelsbüren and Davensberg	**E**	**h1**	**h1**	1.00	-	-	This study
**MTD T 11204**	Germany: North Rhine-Westphalia: between Ammelsbüren and Davensberg	**E**	**h1**	**h1**	1.00	-	-	This study
**MTD T 11408**	Germany: North Rhine-Westphalia: between Ammelsbüren and Davensberg	**E**	**h1**	**h1**	1.00	-	-	This study
**MTD T 11409**	Germany: North Rhine-Westphalia: between Ammelsbüren and Davensberg	**E**	**h1**	**h1**	1.00	-	-	This study
**MTD T 11412**	Germany: North Rhine-Westphalia: between Ammelsbüren and Davensberg	**E**	**h1**	**h1**	0.99	-	-	This study
**MTD T 11414**	Germany: North Rhine-Westphalia: between Ammelsbüren and Davensberg	**E**	**h1**	**h1**	1.00	-	-	This study
**MTD T 11415**	Germany: North Rhine-Westphalia: between Ammelsbüren and Davensberg	**E**	**h1**	**h1**	0.99	-	-	This study
**MTD T 11417**	Germany: North Rhine-Westphalia: between Ammelsbüren and Davensberg	**E**	**h1**	**h1**	1.00	-	-	This study
**MTD T 11420**	Germany: North Rhine-Westphalia: between Ammelsbüren and Davensberg	**E**	**h1**	**h1**	0.99	-	-	This study
**MTD T 11421**	Germany: North Rhine-Westphalia: between Ammelsbüren and Davensberg	**E**	**h1**	**h1**	0.98	-	-	This study
**MTD T 11422**	Germany: North Rhine-Westphalia: between Ammelsbüren and Davensberg	**E**	**h1**	**h1**	0.98	-	-	This study
**MTD T 11424**	Germany: North Rhine-Westphalia: between Ammelsbüren and Davensberg	**E**	**h1**	**h1**	1.00	-	-	This study
**MTD T 11426**	Germany: North Rhine-Westphalia: between Ammelsbüren and Davensberg	**E**	**h1**	**h1**	1.00	-	-	This study
**MTD T 11427**	Germany: North Rhine-Westphalia: between Ammelsbüren and Davensberg	**E**	**h1**	**h1**	0.98	-	-	This study
**MTD T 11429**	Germany: North Rhine-Westphalia: between Ammelsbüren and Davensberg	**E**	**h1**	**h1**	0.99	-	-	This study
**MTD T 11430**	Germany: North Rhine-Westphalia: between Ammelsbüren and Davensberg	**E**	**h1**	**h1**	1.00	-	-	This study
**MTD T 11432**	Germany: North Rhine-Westphalia: between Ammelsbüren and Davensberg	**E**	**h1**	**h1**	0.98	-	-	This study
**ZFMK 75088**	Germany: North Rhine-Westphalia: between Bergisch-Gladbach and Herrenstrunden	**E**	**h1**	-	1.00	-	-	Kindler *et al*. (2013)
**ZFMK 70420**	Germany: North Rhine-Westphalia: between Hürtgenwald and Gey	**E**	**h1**	**h1**	1.00	-	-	Kindler *et al*. (2013)
**MTD T 12494**	Germany: North Rhine-Westphalia: between Ratingen and Düsseldorf	**E**	**h1**	**h1**	1.00	-	-	This study
**ZFMK 68433**	Germany: North Rhine-Westphalia: Bonn	**E**	**h10**	**h1**	0.99	-	-	Kindler *et al*. (2013)
**ZFMK 92228**	Germany: North Rhine-Westphalia: Bonn	**E**	**h1**	-	0.98	-	-	Kindler *et al*. (2013)
**ZFMK 92536**	Germany: North Rhine-Westphalia: Bonn	**E**	**h1**	**h1**	0.96	-	-	Kindler *et al*. (2013)
**ZFMK 92537**	Germany: North Rhine-Westphalia: Bonn	**E**	**h10**	**h1**	0.98	-	-	Kindler *et al*. (2013)
**ZFMK 89393**	Germany: North Rhine-Westphalia: Bonn: Kottenforst	**E**	**h10**	**h1**	0.99	-	-	Kindler *et al*. (2013)
**MTD T 12106**	Germany: North Rhine-Westphalia: Brüchermühle	**E**	**h1**	-	1.00	-	-	This study
**MTD T 12107**	Germany: North Rhine-Westphalia: Brüchermühle	**E**	**h1**	**h1**	0.98	-	-	This study
**MTD T 12108**	Germany: North Rhine-Westphalia: Brüchermühle	**E**	**h1**	-	1.00	-	-	This study
**MTD T 12109**	Germany: North Rhine-Westphalia: Brüchermühle	**E**	**h1**	-	0.99	-	-	This study
**MTD T 12110**	Germany: North Rhine-Westphalia: Brüchermühle	**E**	**h1**	-	1.00	-	-	This study
**MTD T 12111**	Germany: North Rhine-Westphalia: Brüchermühle	**E**	**h1**	-	0.99	-	-	This study
**MTD T 12112**	Germany: North Rhine-Westphalia: Brüchermühle	**E**	**h1**	**h1**	0.98	-	-	This study
**MTD T 12115**	Germany: North Rhine-Westphalia: Brüchermühle	**E**	**h1**	-	1.00	-	-	This study
**MTD T 12117**	Germany: North Rhine-Westphalia: Brüchermühle	**E**	**h1**	-	1.00	-	-	This study
**MTD T 12118**	Germany: North Rhine-Westphalia: Brüchermühle	**E**	**h1**	**h1**	1.00	-	-	This study
**MTD T 12119**	Germany: North Rhine-Westphalia: Brüchermühle	**E**	**h1**	**h1**	0.99	-	-	This study
**MTD T 12120**	Germany: North Rhine-Westphalia: Brüchermühle	**E**	**h1**	**h1**	1.00	-	-	This study
**MTD T 12121**	Germany: North Rhine-Westphalia: Brüchermühle	**E**	**h1**	**h1**	1.00	-	-	This study
**MTD T 12122**	Germany: North Rhine-Westphalia: Brüchermühle	**E**	**h1**	**h1**	0.99	-	-	This study
**MTD T 12123**	Germany: North Rhine-Westphalia: Brüchermühle	**E**	**h1**	-	1.00	-	-	This study
**MTD T 12124**	Germany: North Rhine-Westphalia: Brüchermühle	**E**	**h1**	-	1.00	-	-	This study
**MTD T 12125**	Germany: North Rhine-Westphalia: Brüchermühle	**E**	**h1**	**h1**	1.00	-	-	This study
**MTD T 12126**	Germany: North Rhine-Westphalia: Brüchermühle	**E**	**h1**	**h1**	0.98	-	-	This study
**MTD T 12127**	Germany: North Rhine-Westphalia: Brüchermühle	**E**	**h1**	**h1**	1.00	-	-	This study
**MTD T 12128**	Germany: North Rhine-Westphalia: Brüchermühle	**E**	**h1**	-	1.00	-	-	This study
**MTD T 14059**	Germany: North Rhine-Westphalia: Dortmund	**E**	**h1**	**h1**	1.00	-	-	This study
**MTD T 14060**	Germany: North Rhine-Westphalia: Dortmund	**E**	**h1**	**h1**	1.00	-	-	This study
**MTD T 11981**	Germany: North Rhine-Westphalia: Dortmund: Kirchderne	**E**	**h1**	**h1**	1.00	-	-	This study
**ZFMK 86786**	Germany: North Rhine-Westphalia: Drove	**E**	**h1**	-	1.00	-	-	Kindler *et al*. (2013)
**SMNS 14502**	Germany: North Rhine-Westphalia: Herfterath	**E**	**h1**	**h1**	0.99	-	-	This study
**MTD T 11786**	Germany: North Rhine-Westphalia: Herne	**E**	**h1**	**h1**	0.98	-	-	This study
**MTD T 12021**	Germany: North Rhine-Westphalia: Herne	**E**	**h1**	-	0.98	-	-	This study
**MTD T 12020**	Germany: North Rhine-Westphalia: Königswinter	**E**	**h10**	-	0.94	-	-	This study
**ZFMK 82773**	Germany: North Rhine-Westphalia: Königswinter: Ittenbach	**E**	**h1**	**h17**	0.98	-	-	Kindler *et al*. (2013)
**MTD T 11790**	Germany: North Rhine-Westphalia: Mettmann: Neandertal	**4**	**r4**	-	0.00	0.94	-	This study
**MTD T 11809**	Germany: North Rhine-Westphalia: Mettmann: Neandertal	**4**	**r4**	-	0.00	0.97	0.23	This study
**MTD T 11874**	Germany: North Rhine-Westphalia: Mettmann: Neandertal	**4**	**r4**	**r35**	0.00	0.97	0.23	This study
**MTD T 11879**	Germany: North Rhine-Westphalia: Mettmann: Neandertal	**4**	**r4**	-	-	-	-	This study
**MTD T 11888**	Germany: North Rhine-Westphalia: Mettmann: Neandertal	**4**	**r4**	**r35**	0.00	0.98	0.05	This study
**MTD T 11889**	Germany: North Rhine-Westphalia: Mettmann: Neandertal	**4**	**r4**	**r34**	0.00	0.99	0.17	This study
**MTD T 11890**	Germany: North Rhine-Westphalia: Mettmann: Neandertal	**4**	**r4**	**r35**	0.00	0.97	0.21	This study
**MTD T 11891**	Germany: North Rhine-Westphalia: Mettmann: Neandertal	**4**	**r4**	-	0.00	0.96	0.08	This study
**MTD T 11892**	Germany: North Rhine-Westphalia: Mettmann: Neandertal	**4**	**r4**	-	0.00	0.91	-	This study
**MTD T 11894**	Germany: North Rhine-Westphalia: Mettmann: Neandertal	**4**	**r4**	-	0.00	0.97	0.10	This study
**MTD T 11895**	Germany: North Rhine-Westphalia: Mettmann: Neandertal	**4**	**r4**	-	0.00	0.98	0.06	This study
**MTD T 11574**	Germany: North Rhine-Westphalia: Much	**E**	**h1**	**h1**	1.00	-	-	This study
**MTD T 11575**	Germany: North Rhine-Westphalia: Much	**E**	**h1**	**h1**	1.00	-	-	This study
**MTD T 11576**	Germany: North Rhine-Westphalia: Much	**E**	**h1**	**h1**	1.00	-	-	This study
**MTD T 11577**	Germany: North Rhine-Westphalia: Much	**E**	**h1**	**h1**	0.99	-	-	This study
**MTD T 14087**	Germany: North Rhine-Westphalia: near Dormagen: Knechtstedener Wald	**E**	**h1**	**h1**	1.00	-	-	This study
**MTD T 14088**	Germany: North Rhine-Westphalia: near Dormagen: Knechtstedener Wald	**E**	**h1**	**h1**	1.00	-	-	This study
**MTD T 14089**	Germany: North Rhine-Westphalia: near Dormagen: Knechtstedener Wald	**E**	**h1**	-	-	-	-	This study
**MTD T 14090**	Germany: North Rhine-Westphalia: near Dormagen: Knechtstedener Wald	**E**	-	**h1**	1.00	-	-	This study
**MTD T 14091**	Germany: North Rhine-Westphalia: near Dormagen: Knechtstedener Wald	**E**	**h1**	-	1.00	-	-	This study
**MTD T 14092**	Germany: North Rhine-Westphalia: near Dormagen: Knechtstedener Wald	**E**	**h1**	**h1**	1.00	-	-	This study
**MTD T 14094**	Germany: North Rhine-Westphalia: near Dormagen: Knechtstedener Wald	**E**	**h1**	-	1.00	-	-	This study
**MTD T 14095**	Germany: North Rhine-Westphalia: near Dormagen: Knechtstedener Wald	**E**	**h1**	-	1.00	-	-	This study
**MTD T 14096**	Germany: North Rhine-Westphalia: near Dormagen: Knechtstedener Wald	**E**	**h1**	**h1**	1.00	-	-	This study
**MTD T 14097**	Germany: North Rhine-Westphalia: near Dormagen: Knechtstedener Wald	**E**	**h1**	-	-	-	-	This study
**MTD T 14098**	Germany: North Rhine-Westphalia: near Dormagen: Knechtstedener Wald	**E**	**h1**	-	1.00	-	-	This study
**MTD T 14099**	Germany: North Rhine-Westphalia: near Dormagen: Knechtstedener Wald	**E**	**h1**	-	1.00	-	-	This study
**MTD T 14100**	Germany: North Rhine-Westphalia: near Dormagen: Knechtstedener Wald	**E**	**h1**	**h1**	1.00	-	-	This study
**MTD T 14101**	Germany: North Rhine-Westphalia: near Dormagen: Knechtstedener Wald	**E**	**h1**	-	1.00	-	-	This study
**MTD T 14103**	Germany: North Rhine-Westphalia: near Dormagen: Knechtstedener Wald	**E**	**h1**	**h1**	1.00	-	-	This study
**MTD T 14104**	Germany: North Rhine-Westphalia: near Dormagen: Knechtstedener Wald	**E**	**h1**	**h1**	-	-	-	This study
**MTD T 14105**	Germany: North Rhine-Westphalia: near Dormagen: Knechtstedener Wald	**E**	**h1**	**h1**	1.00	-	-	This study
**MTD T 14106**	Germany: North Rhine-Westphalia: near Dormagen: Knechtstedener Wald	**E**	**h1**	-	1.00	-	-	This study
**MTD T 14107**	Germany: North Rhine-Westphalia: near Dormagen: Knechtstedener Wald	**E**	**h1**	**h1**	-	-	-	This study
**MTD T 14109**	Germany: North Rhine-Westphalia: near Dormagen: Knechtstedener Wald	**E**	**h1**	-	1.00	-	-	This study
**MTD T 14111**	Germany: North Rhine-Westphalia: near Dormagen: Knechtstedener Wald	**E**	**h1**	**h1**	1.00	-	-	This study
**MTD T 14485**	Germany: North Rhine-Westphalia: near Olpe: Friesenhagen	**E**	**h1**	**h1**	0.95	-	-	This study
**ZFMK 83771**	Germany: North Rhine-Westphalia: Nideggen/Eifel	**E**	**h1**	**h1**	0.98	-	-	Kindler *et al*. (2013)
**MTD T 13972**	Germany: North Rhine-Westphalia: Rösrath	**E**	**h1**	**h1**	0.99	-	-	This study
**ZFMK 89086**	Germany: North Rhine-Westphalia: Sankt Augustin-Hangelar	**E**	**h1**	**h1**	0.96	-	-	Kindler *et al*. (2013)
**MTD T 11157**	Germany: North Rhine-Westphalia: Troisdorf	**E**	**h1**	**h1**	-	-	-	This study
**MTD T 11996**	Germany: North Rhine-Westphalia: Troisdorf	**E**	**h1**	**h1**	1.00	-	-	This study
**MTD T 14486**	Germany: North Rhine-Westphalia: Wesel	**E**	**h1**	**h1**	0.97	-	-	This study
**MTD T 11795**	Germany: North Rhine-Westphalia: Wuppertal: Kohlfurt	**E**	**h1**	**h1**	1.00	-	-	This study
**MTD T 11873**	Germany: North Rhine-Westphalia: Wuppertal: Kohlfurt	**E**	**h1**	**h1**	-	-	-	This study
**MTD T 11877**	Germany: North Rhine-Westphalia: Wuppertal: Kohlfurt	**E**	**h1**	-	0.97	-	-	This study
**MTD T 11878**	Germany: North Rhine-Westphalia: Wuppertal: Kohlfurt	**E**	**h1**	**h1**	0.99	-	-	This study
**MTD T 11880**	Germany: North Rhine-Westphalia: Wuppertal: Kohlfurt	**E**	**h1**	**h1**	1.00	-	-	This study
**MTD T 11881**	Germany: North Rhine-Westphalia: Wuppertal: Kohlfurt	**E**	**h1**	**h1**	1.00	-	-	This study
**MTD T 11883**	Germany: North Rhine-Westphalia: Wuppertal: Kohlfurt	**E**	**h1**	**h1**	1.00	-	-	This study
**MTD T 11884**	Germany: North Rhine-Westphalia: Wuppertal: Kohlfurt	**E**	**h1**	-	0.96	-	-	This study
**MTD T 11886**	Germany: North Rhine-Westphalia: Wuppertal: Kohlfurt	**E**	**h1**	**h1**	0.99	-	-	This study
**MTD T 11887**	Germany: North Rhine-Westphalia: Wuppertal: Kohlfurt	**E**	**h1**	**h1**	1.00	-	-	This study
**MTD T 11904**	Germany: North Rhine-Westphalia: Wuppertal: Kohlfurt	**E**	**h1**	**h1**	1.00	-	-	This study
**MTD T 11905**	Germany: North Rhine-Westphalia: Wuppertal: Kohlfurt	**E**	**h1**	**h1**	1.00	-	-	This study

• Table S1 continued

Voucher	Locality	mtDNA data			Microsatellite data			Reference
		mtDNA clade	ND4 haplotype	cyt *b* haplotype	First STRUCTURE run	Second STRUCTURE run	Third STRUCTURE run	
MTD T 11907	Germany: North Rhine-Westphalia: Wuppertal: Kohlfurt	E	h1	-	1.00	-	-	This study
MTD T 12043	Germany: North Rhine-Westphalia: Wuppertal: Kohlfurt	E	h1	-	-	-	-	This study
MTD T 12045	Germany: North Rhine-Westphalia: Wuppertal: Kohlfurt	E	h1	-	0.99	-	-	This study
MTD T 12048	Germany: North Rhine-Westphalia: Wuppertal: Kohlfurt	E	h1	-	1.00	-	-	This study
MTD T 12049	Germany: North Rhine-Westphalia: Wuppertal: Kohlfurt	E	h1	-	1.00	-	-	This study
MTD T 12050	Germany: North Rhine-Westphalia: Wuppertal: Kohlfurt	E	h1	h1	0.99	-	-	This study
MTD T 12051	Germany: North Rhine-Westphalia: Wuppertal: Kohlfurt	E	h1	h1	1.00	-	-	This study
MTD T 12052	Germany: North Rhine-Westphalia: Wuppertal: Kohlfurt	E	h1	h1	1.00	-	-	This study
MTD T 12053	Germany: North Rhine-Westphalia: Wuppertal: Kohlfurt	E	h1	h1	0.93	-	-	This study
MTD T 12054	Germany: North Rhine-Westphalia: Wuppertal: Kohlfurt	E	h1	-	1.00	-	-	This study
ZFMK 73645	Germany: North Rhine-Westphalia: Wuppertal: Morsbachtal	E	h1	h1	1.00	-	-	Kindler *et al.* (2013)
MTD T 12057	Germany: North Rhine-Westphalia: Wuppertal: Neu-Dornap	E	h1	-	1.00	-	-	This study
ZFMK 73646	Germany: North Rhine-Westphalia: Wuppertal: Vorwerk	E	h1	h1	1.00	-	-	Kindler *et al.* (2013)
MTD T 14838	Germany: Rhineland-Palatinate: Altenahr	E	h5	h1	-	-	-	This study
MTD T 12042	Germany: Rhineland-Palatinate: between Oberpleis and Eudenbach	E	h1	-	-	-	-	This study
SMF 56593	Germany: Rhineland-Palatinate: Bürdenbach	E	h1	h1	0.89	-	-	This study
SMNS 14504	Germany: Rhineland-Palatinate: Germersheim	E	h1	h1	1.00	-	-	This study
ZFMK 80876	Germany: Rhineland-Palatinate: Horhausen	E	h1	h1	0.92	-	-	Kindler *et al.* (2013)
MTD D 35933	Germany: Rhineland-Palatinate: Lieser	E	h1	h1	1.00	-	-	Kindler *et al.* (2013)
ZFMK 83703	Germany: Rhineland-Palatinate: Maria Laach	E	h1	h1	-	-	-	Kindler *et al.* (2013)
SMF 73299	Germany: Rhineland-Palatinate: Nauroth	E	h1	h1	1.00	-	-	This study
MTD T 11573	Germany: Rhineland-Palatinate: near Dreis	E	h1	h1	1.00	-	-	This study
MTD T 11758	Germany: Rhineland-Palatinate: near Linz: St. Katharinen	E	h1	-	-	-	-	This study
SMF 74940	Germany: Rhineland-Palatinate: SW Koblenz: between Macken and Burgen	E	h1	h1	0.99	-	-	This study
ZFMK 80875	Germany: Rhineland-Palatinate: Weltersburg	E	h1	-	1.00	-	-	Kindler *et al.* (2013)
ZFMK 54321	Germany: Saarland: Hirschberg/Neunkirchen	E	-	h28	0.99	-	-	Kindler *et al.* (2013)
MTD T 14481	Germany: Saxony: Altenberg: Bärenstein	4	r3	r10	0.00	0.99	0.82	This study
MTD D 39712	Germany: Saxony: Bad Düben	3	y1	HF680028	-	-	-	Kindler *et al.* (2013)
MTD D 39331	Germany: Saxony: Bad Gottleuba	4	r3	r17	0.00	0.98	0.19	Kindler *et al.* (2013)
MTD D 47231	Germany: Saxony: Bärnsdorf	4	r3	r10	0.00	0.98	0.49	Kindler *et al.* (2013)
MTD T 10923	Germany: Saxony: between Burkhartswalde and Obermunzig	3	y1	y5	0.01	0.81	-	This study
MTD T 14545	Germany: Saxony: between Mohorn and Helbigsdorf	4	r4	r34	0.00	0.99	0.34	This study
MTD T 10997	Germany: Saxony: between Volkersdorf and Boxdorf	4	r28	r10	0.00	0.98	0.91	This study
MTD T 10998	Germany: Saxony: between Volkersdorf and Boxdorf	4	r3	r10	-	-	-	This study
MTD T 3183	Germany: Saxony: Biehla	3	y1	y6	0.00	0.98	0.23	Kindler *et al.* (2013)
MTD D 31066	Germany: Saxony: Borna	4	r3	r17	0.02	0.96	0.15	Kindler *et al.* (2013)
MWLK 192/13	Germany: Saxony: Burkau	3	y2	y5	0.00	0.98	0.62	This study
MTD D 47317	Germany: Saxony: Chemnitz	3	y9	y5	0.00	0.99	0.64	Kindler *et al.* (2013)
MTD D 47318	Germany: Saxony: Chemnitz	3	-	y5	0.00	0.99	0.93	Kindler *et al.* (2013)
MWLK 803/05	Germany: Saxony: Cunnersdorf	4	r3	r10	0.00	0.94	-	This study
MWLK 626/05	Germany: Saxony: Cunnersdorf-Biehla	3	y2	y5	0.00	0.99	0.38	Kindler *et al.* (2013)
MTD D 46238	Germany: Saxony: Dahlen	3	y1	y5	0.00	0.96	0.72	Kindler *et al.* (2013)
MWLK 588/11	Germany: Saxony: Döbra	3	y1	y6	0.00	0.98	0.85	This study
MTD D 47638	Germany: Saxony: Dorfhain	4	r3	r10	0.00	0.99	0.65	Kindler *et al.* (2013)
MTD D 45527	Germany: Saxony: Dresden	3	-	y27	0.00	0.97	0.18	Kindler *et al.* (2013)
MTD D 47639	Germany: Saxony: Dresden	4	r3	r10	0.01	0.98	0.75	Kindler *et al.* (2013)
MTD D 47728	Germany: Saxony: Dresden	3	y1	-	0.00	0.99	0.78	Kindler *et al.* (2013)
MTD T 11539	Germany: Saxony: Dresden	3	y1	y27	0.02	0.99	0.86	This study
MTD T 13025	Germany: Saxony: Dresden	4	r3	r10	0.00	0.99	0.60	This study
MTD T 13090	Germany: Saxony: Dresden	3	y1	y27	0.00	0.98	0.65	This study
MTD T 14057	Germany: Saxony: Dresden	4	r3	r10	-	-	-	This study
MTD T 14487	Germany: Saxony: Dresden	4	r3	r10	0.00	0.99	0.12	This study
MTD T 11159	Germany: Saxony: Dresden: Airport	4	r3	r10	0.00	0.99	0.87	This study
MTD T 12816	Germany: Saxony: Dresden: Klotzsche	4	r3	r10	0.02	0.98	0.77	This study
MTD T 12832	Germany: Saxony: Dresden: Klotzsche	4	r3	r10	0.00	0.99	0.79	This study
MWLK 76/08	Germany: Saxony: Dresden: Klotzsche	3	y1	y27	-	-	-	This study
MTD D 48178	Germany: Saxony: Dresden: Langebrück	4	r4	r34	0.00	0.98	0.57	Kindler *et al.* (2013)
MTD T 12835	Germany: Saxony: Dresden: Langebrück	3	y1	y27	0.00	0.98	0.22	This study
MWLK 665/11	Germany: Saxony: Dresden: Langebrück	3	y1	-	-	-	-	This study
MTD D 47258	Germany: Saxony: Dresden: Weixdorf	4	r3	r10	0.02	0.98	0.87	Kindler *et al.* (2013)
MTD D 48756	Germany: Saxony: Dresden: Weixdorf	3	y1	y27	0.00	0.99	0.76	This study
MWLK 250/08	Germany: Saxony: Droben	3	y1	y8	0.00	0.99	0.40	This study
MWLK 399/01	Germany: Saxony: Dubring - Scheckthal	3	y1	y6	-	-	-	This study
MWLK 51/04	Germany: Saxony: Dubringer Moor	3	y1	y6	0.00	0.98	0.58	Kindler *et al.* (2013)
MWLK 432/08	Germany: Saxony: Fischbach	4	r3	r10	0.00	0.99	0.31	This study
MWLK 265/08	Germany: Saxony: Groß Neida	3	y1	y5	0.00	0.98	0.55	This study
MWLK 266/08	Germany: Saxony: Groß Neida	3	y1	-	0.00	0.99	0.40	This study
MTD D 38701	Germany: Saxony: Großdittmannsdorf	4	-	r34	-	-	-	Kindler *et al.* (2013)
MWLK 370/04	Germany: Saxony: Großdittmannsdorf	4	r3	r10	-	-	-	Kindler *et al.* (2013)
MWLK 776/05	Germany: Saxony: Guttau	3	y1	y5	0.00	0.92	-	Kindler *et al.* (2013)
MTD D 47002	Germany: Saxony: Hartmannsgrün	3	-	y5	0.00	0.99	0.67	Kindler *et al.* (2013)
MTD T 14752	Germany: Saxony: Helbigsdorf	4	r4	r59	0.00	0.99	0.88	This study
MWLK 794/05	Germany: Saxony: Jetscheba	3	y1	y5	0.01	0.93	-	This study
MWLK 182/12	Germany: Saxony: Kamenz	3	y2	y5	0.00	0.97	0.29	This study
MWLK 466/05	Germany: Saxony: Kamenz	4	r3	-	0.00	0.99	0.46	Kindler *et al.* (2013)
MWLK 988/05	Germany: Saxony: Kamenz	3	y2	y5	0.00	0.97	0.26	Kindler *et al.* (2013)
MTD T 10924	Germany: Saxony: Karsdorf	4	r3	r10	0.00	0.99	0.61	This study
MTD T 10925	Germany: Saxony: Karsdorf	4	r3	r10	0.01	0.99	0.26	This study
MTD T 10926	Germany: Saxony: Karsdorf	3	y1	y5	0.01	0.98	0.09	This study
MTD T 10927	Germany: Saxony: Karsdorf	4	r3	r10	-	-	-	This study
MTD T 10928	Germany: Saxony: Karsdorf	4	r3	r10	-	-	-	This study
MTD T 10929	Germany: Saxony: Karsdorf	4	r4	r34	0.01	0.99	0.22	This study
MTD T 10930	Germany: Saxony: Karsdorf	3	y1	y5	0.01	0.98	0.62	This study
MTD T 10932	Germany: Saxony: Karsdorf	4	r4	r34	0.00	0.99	0.90	This study
MTD T 10933	Germany: Saxony: Karsdorf	4	r3	r10	0.00	0.99	0.30	This study
MTD T 10934	Germany: Saxony: Karsdorf	4	r3	r17	-	-	-	This study
MTD T 10843	Germany: Saxony: Kleba	4	r3	r10	0.00	0.90	-	This study
MTD T 14350	Germany: Saxony: Klingenberg	4	r3	r10	0.00	0.98	0.20	This study
MTD T 14351	Germany: Saxony: Klingenberg	4	r4	r34	0.00	0.99	0.14	This study
MTD T 14444	Germany: Saxony: Klingenberg	4	r3	r10	0.00	0.99	0.83	This study
MTD T 14445	Germany: Saxony: Klingenberg	4	r3	r10	0.00	0.98	0.09	This study
MTD D 40611	Germany: Saxony: Kreischa-Lockmittel	4	r3	r10	0.00	0.99	0.61	Kindler *et al.* (2013)
MTD D 45996	Germany: Saxony: Lauterbach	3	-	y5	0.00	0.99	0.95	Kindler *et al.* (2013)
MTD T 9528	Germany: Saxony: Lengefeld / Neunzehnhain	4	r3	r11	0.00	0.98	0.73	Kindler *et al.* (2013)
MWLK 343/10	Germany: Saxony: Liebegast	3	y1	y27	0.01	0.99	0.86	This study
MTD D 48837	Germany: Saxony: Liegau-Augustusbad	4	r3	r10	0.00	0.99	0.20	This study
MTD T 10999	Germany: Saxony: Liegau-Augustusbad	4	r3	r10	0.00	0.99	0.22	This study
MTD T 11227	Germany: Saxony: Liegau-Augustusbad	4	r3	r10	0.00	0.98	0.50	This study
MTD T 11461	Germany: Saxony: Liegau-Augustusbad	4	r3	r10	0.00	0.97	0.30	This study
MTD T 12782	Germany: Saxony: Liegau-Augustusbad	3	y1	y27	0.00	0.98	0.28	This study
MTD T 13332	Germany: Saxony: Liegau-Augustusbad	3	y1	y27	0.00	0.97	0.33	This study
MWLK 76/01	Germany: Saxony: Linz	4	r3	r10	0.00	0.99	0.52	Kindler *et al.* (2013)
MTD T 10936	Germany: Saxony: Medingen	3	y1	y27	0.00	0.98	0.35	This study
MTD T 11106	Germany: Saxony: Meißen	3	y1	y5	0.08	-	-	This study
MTD T 11107	Germany: Saxony: Meißen	4	r3	r10	0.00	0.99	0.95	This study
MTD T 11108	Germany: Saxony: Meißen	4	r3	r10	0.00	0.98	0.26	This study
MTD T 11396	Germany: Saxony: Meißen	4	r3	r10	0.00	0.98	0.20	This study
MTD T 11397	Germany: Saxony: Meißen	3	y1	y5	0.00	0.99	0.56	This study
MTD T 11398	Germany: Saxony: Meißen	4	r3	r14	0.00	0.98	0.66	This study
MTD T 11400	Germany: Saxony: Meißen	4	r3	r14	-	-	-	This study
MWLK 371/01	Germany: Saxony: Michalken	3	y1	y5	0.00	0.98	0.22	Kindler *et al.* (2013)
MWLK 372/01	Germany: Saxony: Michalken	3	y1	y6	0.00	0.97	0.45	Kindler *et al.* (2013)
MWLK 624/07	Germany: Saxony: Michalken	3	y1	y5	0.01	0.99	0.51	This study
MTD D 45006	Germany: Saxony: Milkel	3	y1	y6	-	-	-	Kindler *et al.* (2013)
MWLK 793/05	Germany: Saxony: Milkel	3	y1	y8	0.00	0.98	0.35	This study
MWLK 1032/05	Germany: Saxony: Mönau	3	y1	y5	0.00	0.99	0.92	This study
MWLK 1049/05	Germany: Saxony: Mönau	3	y1	y5	0.01	0.96	0.20	This study
MTD T 10770	Germany: Saxony: Moritzburg	4	r4	r34	0.01	0.73	-	This study
MTD T 10771	Germany: Saxony: Moritzburg	4	r4	r34	0.00	0.99	0.71	This study
MTD T 10772	Germany: Saxony: Moritzburg	4	r4	r44	0.01	0.99	0.58	This study
MTD T 10773	Germany: Saxony: Moritzburg	4	r4	r34	0.01	0.99	0.92	This study
MTD T 10774	Germany: Saxony: Moritzburg	3	y1	y5	0.00	0.92	-	This study
MTD T 11031	Germany: Saxony: Moritzburg	4	r3	r10	0.00	0.99	0.39	This study
MTD T 11032	Germany: Saxony: Moritzburg	4	r4	r34	0.01	0.98	0.32	This study

• **Table S1 continued**

Voucher	Locality	mtDNA data			Microsatellite data			Reference
		mtDNA clade	ND4 haplotype	cyt *b* haplotype	First STRUCTURE run	Second STRUCTURE run	Third STRUCTURE run	
**MTD T 11033**	Germany: Saxony: Moritzburg	4	r4	r34	0.00	0.99	0.10	This study
**MTD T 11035**	Germany: Saxony: Moritzburg	4	r4	r34	0.00	0.99	0.92	This study
**MWLK 665/11**	Germany: Saxony: Naunhof	4	r3	r10	0.01	0.98	0.75	This study
**MTD T 11395**	Germany: Saxony: near Dresden	3	y1	y27	0.01	0.93	-	This study
**MWLK 105/01**	Germany: Saxony: Neudorf	3	y1	y5	0.00	0.96	0.73	This study
**MWLK 311/05**	Germany: Saxony: Neukirch	3	y1	y7	0.00	0.95	0.25	Kindler *et al.* (2013)
**MTD D 47429**	Germany: Saxony: Oberschöna	4	r3	r10	-	-	-	Kindler *et al.* (2013)
**MTD D 32501**	Germany: Saxony: Oppach	3	y1	y7	-	-	-	Kindler *et al.* (2013)
**MWLK 141/09**	Germany: Saxony: Oppitz	3	y1	y19	0.01	0.98	0.33	This study
**MTD D 42679**	Germany: Saxony: Ottendorf-Okrilla	3	y1	y7	0.00	0.99	0.63	Kindler *et al.* (2013)
**MTD T 11222**	Germany: Saxony: Ottendorf-Okrilla	3	y1	y6	-	-	-	This study
**MTD T 14857**	Germany: Saxony: Pirk: dam	3	y9	y5	0.00	0.98	0.50	This study
**MWLK 378/10**	Germany: Saxony: Prachenau	3	y1	y7	0.00	0.98	0.74	This study
**MWLK 297/10**	Germany: Saxony: Pulsnitz	4	r3	r10	0.00	0.82	-	This study
**MWLK 284/99**	Germany: Saxony: Rehnsdorf	3	y2	y5	0.00	0.99	0.53	Kindler *et al.* (2013)
**MTD T 13915**	Germany: Saxony: Rietschen	3	y3	y5	0.00	0.99	0.60	This study
**MTD T 13916**	Germany: Saxony: Rietschen	3	y41	y5	0.00	0.98	0.49	This study
**MWLK 194/09**	Germany: Saxony: Röhrsdorf	4	r3	r10	0.00	0.91	-	This study
**MWLK 68/02**	Germany: Saxony: Schönau	3	y1	y19	0.00	0.98	0.25	This study
**MWLK 1486/06**	Germany: Saxony: Schönbach-Bulleritz	3	y2	y5	0.00	0.99	0.29	This study
**MTD T 11030**	Germany: Saxony: Schönborn	3	y1	y6	0.00	0.99	0.06	This study
**MWLK 53/04**	Germany: Saxony: Schwepnitz	3	y3	y5	0.00	0.95	0.39	Kindler *et al.* (2013)
**MTD T 12833**	Germany: Saxony: Seilitz	4	r4	r34	0.01	0.98	0.86	This study
**MWLK 137/12**	Germany: Saxony: Skaska	3	y1	y6	0.00	0.97	0.46	This study
**MWLK 204/12**	Germany: Saxony: Skaska	3	y1	y6	0.00	0.98	0.93	This study
**MWLK 687/11**	Germany: Saxony: Skaska	3	y1	y5	0.01	0.99	0.77	This study
**MWLK 537/11**	Germany: Saxony: Steinitz	3	y1	y5	0.00	0.90	-	This study
**MWLK 483/07**	Germany: Saxony: Straßgräbchen	3	y2	y5	0.00	0.99	0.25	This study
**MWLK 795/05**	Germany: Saxony: Teicha	3	-	y5	0.00	0.97	0.13	Kindler *et al.* (2013)
**MTD T 13015**	Germany: Saxony: Tharandt	4	r3	r10	0.00	0.99	0.19	This study
**MWLK 1047/05**	Germany: Saxony: Treugeböhla	3	y14	y5	0.00	0.98	0.76	This study
**MWLK 10/13**	Germany: Saxony: Uhyst - Mönau	3	y32	y7	0.00	0.96	0.72	This study
**MWLK 447/11**	Germany: Saxony: Uhyst - Mönau	3	y1	y5	0.00	0.99	0.61	This study
**MTD T 12185**	Germany: Saxony: w Niesky	3	-	y5	0.00	0.98	0.77	This study
**MTD D 42680**	Germany: Saxony: Waldenburg	3	y1	y15	0.00	0.99	0.73	Kindler *et al.* (2013)
**MWLK 1050/05**	Germany: Saxony: Wartha-Steinitz	3	y26	y6	0.00	0.90	-	This study
**MWLK 115/09**	Germany: Saxony: Weinböhla	3	y1	y1	0.01	0.99	0.74	This study
**MTD T 10775**	Germany: Saxony: Wermsdorf	3	y1	y5	0.00	0.97	0.47	This study
**MTD T 10994**	Germany: Saxony: Wermsdorf	3	y1	y5	0.00	0.99	0.81	This study
**MTD T 10995**	Germany: Saxony: Wermsdorf	4	r3	-	0.00	0.98	0.60	This study
**MTD T 10996**	Germany: Saxony: Wermsdorf	4	r3	r10	0.00	0.98	0.35	This study
**MTD T 11223**	Germany: Saxony: Wermsdorf	4	r3	r10	0.00	0.83	-	This study
**MWLK 815/05**	Germany: Saxony: Wilthen	3	y1	y5	0.00	0.99	0.11	This study
**MWLK 144/01**	Germany: Saxony: Wittichenau	3	y1	y5	0.00	0.97	0.13	Kindler *et al.* (2013)
**MTD D 41418**	Germany: Saxony-Anhalt: Altenbrak	3	y1	y17	0.01	0.98	0.84	Kindler *et al.* (2013)
**MTD T 11578**	Germany: Saxony-Anhalt: near Klobikau: Lake Geißeltal	3	y1	y5	0.00	0.95	0.64	This study
**ZFMK 89087**	Germany: Saxony-Anhalt: Sangerhausen	3	y1	y5	0.00	0.99	0.33	Kindler *et al.* (2013)
**MTD T 14072**	Germany: Schleswig-Holstein: Bad Bramstedt	3	y1	y20	0.00	0.99	0.92	This study
**ZMH R09486**	Germany: Schleswig-Holstein: Bargteheide	3	y1	y19	0.08	-	-	This study
**ZMH R09497**	Germany: Schleswig-Holstein: Bargteheide	3	y1	y20	0.01	0.99	0.85	This study
**ZMH R09855**	Germany: Schleswig-Holstein: Bargteheide	3	y1	y20	0.00	0.99	0.87	This study
**ZMH R11001**	Germany: Schleswig-Holstein: Bargteheide	3	y1	y20	0.02	0.99	0.90	This study
**MTD T 14846**	Germany: Schleswig-Holstein: Bark: Barker Heide	3	y1	y20	0.01	0.95	0.66	This study
**MTD T 14847**	Germany: Schleswig-Holstein: Dägeling: Heide Nordoe	3	y1	y20	0.01	0.94	-	This study
**MTD T 14070**	Germany: Schleswig-Holstein: Daldorf: Kiebitzholmer Moor	3	y1	y20	0.01	0.98	0.96	This study
**MTD T 14845**	Germany: Schleswig-Holstein: Dätgen	3	y11	y20	0.01	0.98	0.67	This study
**ZMH R09046**	Germany: Schleswig-Holstein: Dithmarschen	3	y1	-	-	-	-	This study
**MTD T 14073**	Germany: Schleswig-Holstein: Felm: Kaltenhofer Moor	3	y1	y21	0.02	0.99	0.91	This study
**MTD T 14074**	Germany: Schleswig-Holstein: Felm: Kaltenhofer Moor	3	y1	y21	0.00	0.99	0.94	This study
**MTD T 14848**	Germany: Schleswig-Holstein: Felm: Kaltenhofer Moor	3	y1	y21	0.00	0.99	0.94	This study
**MTD T 14071**	Germany: Schleswig-Holstein: Gribbohm	3	y11	-	0.01	0.96	0.89	This study
**MTD T 14066**	Germany: Schleswig-Holstein: Hamdorf	3	y11	y20	0.00	0.99	0.88	This study
**MTD T 14069**	Germany: Schleswig-Holstein: Hamdorf	3	y1	y20	0.01	0.98	0.83	This study
**ZFMK 85184**	Germany: Schleswig-Holstein: Kiel: Elmschenhagen	3	y1	y20	0.00	0.99	0.95	Kindler *et al.* (2013)
**ZFMK 62405**	Germany: Schleswig-Holstein: Kiel: Landwehr	3	y1	HF680066	0.00	0.99	0.82	Kindler *et al.* (2013)
**MTD T 13934**	Germany: Schleswig-Holstein: Klein Rönnau	3	y1	-	0.00	0.99	0.95	This study
**ZMH R09338**	Germany: Schleswig-Holstein: Langenhorst	3	y1	y19	0.00	0.99	0.95	This study
**MTD T 14068**	Germany: Schleswig-Holstein: Lübeck: Grönauer Heide	3	y1	y20	0.00	0.99	0.92	This study
**MTD T 13935**	Germany: Schleswig-Holstein: Lübeck: Schellbruch	3	y1	y20	0.01	0.95	0.85	This study
**MTD T 13936**	Germany: Schleswig-Holstein: Lübeck: Schellbruch	3	y1	y20	0.01	0.99	0.96	This study
**MTD T 14063**	Germany: Schleswig-Holstein: Osterrönfeld	3	y1	-	0.00	0.99	0.92	This study
**ZFMK 92535**	Germany: Schleswig-Holstein: Probstei: Hagener Moor	3	y1	y41	0.00	0.99	0.80	Kindler *et al.* (2013)
**ZFMK 73639**	Germany: Schleswig-Holstein: Probsteierhagen	3	y1	y41	0.00	0.99	0.87	Kindler *et al.* (2013)
**ZMH R04381**	Germany: Schleswig-Holstein: Rendsburg-Eckernförde: Warder	3	y1	y5	0.00	0.99	0.76	This study
**MTD T 14064**	Germany: Schleswig-Holstein: Sehestedt	3	y1	y20	0.00	0.99	0.95	This study
**NME R 0409/02**	Germany: Thuringia: Cospeda	3	y1	y5	0.00	0.99	0.45	This study
**NME R 0445/04**	Germany: Thuringia: Erfurt: Rhoda	3	y1	y5	0.00	0.97	0.76	This study
**NME R 0507/07**	Germany: Thuringia: Erfurt: Rhoda	3	y1	y5	0.00	0.99	0.53	This study
**NME R 0556/08**	Germany: Thuringia: Erfurt: Rhoda	3	y1	y5	0.00	0.98	0.69	This study
**NME R 0696/11**	Germany: Thuringia: Geroda	3	y1	y5	0.00	0.98	0.84	This study
**NME R 0793/13**	Germany: Thuringia: Großheringen	3	y1	y5	0.00	0.97	0.93	This study
**NME R 0956/14**	Germany: Thuringia: Heroldsdorf	3	y1	y5	-	-	-	This study
**NME R 0415/02**	Germany: Thuringia: Rotheul	3	y1	y16	0.00	0.99	0.11	This study
**MTD T 12792**	Germany: Thuringia: Schömbach dam	3	y1	y4	0.00	0.98	0.83	This study
**NME R 0253/99**	Germany: Thuringia: Triebes	3	y1	y5	-	-	-	This study
**NME R 0676/10**	Germany: Thuringia: Triebes	3	y1	y5	0.00	0.98	0.55	This study
**NME R 0495/06**	Germany: Thuringia: Uhlstädt-Kirchhasel	3	y1	-	-	-	-	This study
**NME R 0890/13**	Germany: Thuringia: Wandersleben	3	y1	y24	0.06	-	-	This study
**NME R 0413/02**	Germany: Thuringia: Zeulenroda-Triebes	3	y9	y5	0.00	0.99	0.59	This study
**MTD T 14169**	Great Britain	E	h1	h21	-	-	-	This study
**MTD T 14807**	Great Britain: Brecklands area of Norfolk: near Thetford	E	h1	-	-	-	-	This study
**MTD T 14808**	Great Britain: Brecklands area of Norfolk: near Thetford	E	h1	h7	1.00	-	-	This study
**MTD T 14809**	Great Britain: Brecklands area of Norfolk: near Thetford	E	h1	h1	1.00	-	-	This study
**MTD T 14810**	Great Britain: Brecklands area of Norfolk: near Thetford	E	h1	h1	1.00	-	-	This study
**MTD T 14811**	Great Britain: Brecklands area of Norfolk: near Thetford	E	h1	h1	1.00	-	-	This study
**MTD T 14812**	Great Britain: Brecklands area of Norfolk: near Thetford	E	h1	h1	1.00	-	-	This study
**MTD T 14130**	Great Britain: Buckingshamshire: Chesham	E	h1	h1	1.00	-	-	This study
**MTD T 14144**	Great Britain: Denbighshire: Eryrys	E	h1	-	1.00	-	-	This study
**MTD T 14165**	Great Britain: Dorset	E	h1	h1	1.00	-	-	This study
**MTD T 14166**	Great Britain: Dorset	E	h1	h1	0.99	-	-	This study
**MTD T 14131**	Great Britain: Dorset: Bovington	E	h6	-	-	-	-	This study
**MTD T 14132**	Great Britain: Dorset: Christchurch	E	h1	h1	0.99	-	-	This study
**MTD T 14117**	Great Britain: Essex: Loughton	E	h1	h1	1.00	-	-	This study
**MTD T 14118**	Great Britain: Essex: Loughton	E	h1	h1	1.00	-	-	This study
**MTD T 14119**	Great Britain: Essex: Loughton	E	h1	h1	1.00	-	-	This study
**MTD T 14120**	Great Britain: Essex: Loughton	E	h1	h1	1.00	-	-	This study
**MTD T 14121**	Great Britain: Essex: Loughton	E	h1	h1	1.00	-	-	This study
**MTD T 14122**	Great Britain: Essex: Loughton	E	h1	h1	1.00	-	-	This study
**MTD T 14136**	Great Britain: Gloucestershire: Milkwall	E	h1	h1	1.00	-	-	This study
**MTD T 14124**	Great Britain: Hereford: 4km E Ledbury	E	h1	h1	1.00	-	-	This study
**MTD T 14123**	Great Britain: Hereford: Ledbury	E	h2	h1	1.00	-	-	This study
**MTD T 14139**	Great Britain: Herefordshire: Abbey Dore	E	h1	h1	1.00	-	-	This study
**MTD T 14167**	Great Britain: Herefordshire: Hatfield	E	h1	-	1.00	-	-	This study
**MTD T 14137**	Great Britain: Herefordshire: Tedstone Wafre	E	h1	h21	0.97	-	-	This study
**MTD T 13012**	Great Britain: Hull	E	h6	h1	1.00	-	-	This study
**MTD T 13011**	Great Britain: Hull	E	h6	h1	1.00	-	-	This study
**MTD T 14133**	Great Britain: Kent: Hildenborough	E	h1	-	1.00	-	-	This study
**MTD T 14126**	Great Britain: near Scotland	E	h1	h1	-	-	-	This study
**MTD T 14127**	Great Britain: near Scotland	E	h1	h1	-	-	-	This study
**MTD T 14128**	Great Britain: near Scotland	E	h1	h1	-	-	-	This study
**MTD T 14421**	Great Britain: Norfolk: Aylsham	E	-	h1	1.00	-	-	This study
**MTD T 14116**	Great Britain: Norfolk: Cirby Cane	E	h1	h1	1.00	-	-	This study
**MTD T 9982**	Great Britain: North Wales: Gwaith Powder	E	h1	-	1.00	-	-	Kindler *et al.* (2013)
**MTD T 9983**	Great Britain: North Wales: Gwaith Powder	E	h1	-	1.00	-	-	Kindler *et al.* (2013)

• Table S1 continued

Voucher	Locality	mtDNA data			Microsatellite data			Reference
		mtDNA clade	ND4 haplotype	cyt *b* haplotype	First STRUCTURE run	Second STRUCTURE run	Third STRUCTURE run	
**MTD T 14141**	Great Britain: Northhamptonshire: Sywell	**E**	**h1**	-	1.00	-	-	This study
**MTD T 14140**	Great Britain: Nottinghamshire: Jacksdale	**E**	**h1**	**h1**	1.00	-	-	This study
**MTD T 14113**	Great Britain: Pembrokeshire: Talbenny	**E**	**h1**	-	0.92	-	-	This study
**MTD T 14142**	Great Britain: Staffordshire: between Wombourn and Sedgley	**E**	**h1**	**h1**	1.00	-	-	This study
**MTD T 14163**	Great Britain: Suffolk: Martlesham	**E**	**h1**	**h1**	1.00	-	-	This study
**MTD T 14164**	Great Britain: Suffolk: Martlesham	**E**	**h1**	**h1**	1.00	-	-	This study
**MTD T 14134**	Great Britain: Surrey: Milford	**E**	**h1**	**h1**	1.00	-	-	This study
**MTD T 14147**	Great Britain: Surrey: Newdigate	C	**c1**	**c1**	0.98	-	-	This study
**MTD T 14148**	Great Britain: Surrey: Newdigate	**E**	**h1**	**h1**	0.73	-	-	This study
**MTD T 14149**	Great Britain: Surrey: Newdigate	**E**	**h1**	**h21**	0.96	-	-	This study
**MTD T 14150**	Great Britain: Surrey: Newdigate	**E**	**h1**	**h1**	0.99	-	-	This study
**MTD T 14151**	Great Britain: Surrey: Newdigate	**E**	**h8**	**h1**	0.90	-	-	This study
**MTD T 14152**	Great Britain: Surrey: Newdigate	C	**c1**	**c1**	0.84	-	-	This study
**MTD T 14153**	Great Britain: Surrey: Newdigate	C	**c1**	**c1**	1.00	-	-	This study
**MTD T 14154**	Great Britain: Surrey: Newdigate	**E**	**h1**	**h21**	0.99	-	-	This study
**MTD T 14155**	Great Britain: Surrey: Newdigate	**E**	**h8**	**h1**	0.98	-	-	This study
**MTD T 14156**	Great Britain: Surrey: Newdigate	**E**	**h1**	**h21**	0.98	-	-	This study
**MTD T 14157**	Great Britain: Surrey: Newdigate	C	**c1**	**c1**	0.94	-	-	This study
**MTD T 14158**	Great Britain: Surrey: Newdigate	**E**	**h1**	**h1**	0.98	-	-	This study
**MTD T 14159**	Great Britain: Surrey: Newdigate	**E**	**h1**	**h1**	0.98	-	-	This study
**MTD T 14160**	Great Britain: Surrey: Newdigate	**E**	**h1**	**h21**	0.96	-	-	This study
**MTD T 14161**	Great Britain: Surrey: Newdigate	**E**	**h1**	**h21**	0.82	-	-	This study
**MTD T 14464**	Great Britain: Surrey: Newdigate	**E**	**h1**	**h21**	0.99	-	-	This study
**MTD T 14465**	Great Britain: Surrey: Newdigate	**E**	**h1**	**h1**	0.83	-	-	This study
**MTD T 14466**	Great Britain: Surrey: Newdigate	F	**f1**	**f1**	0.93	-	-	This study
**MTD T 14467**	Great Britain: Surrey: Newdigate	**4**	**r4**	**r34**	1.00	-	-	This study
**MTD T 14468**	Great Britain: Surrey: Newdigate	**E**	**h1**	**h1**	0.99	-	-	This study
**MTD T 14135**	Great Britain: Sussex: Heathfield	**E**	**h1**	**h1**	1.00	-	-	This study
**MTD T 14143**	Great Britain: Warwickshire: Oxhill	**E**	**h1**	**h1**	1.00	-	-	This study
**MTD T 14129**	Great Britain: Yorkshire: Bradford	**E**	**h1**	**h1**	1.00	-	-	This study
**MTD T 14359**	Great Britain: Yorkshire: Skipwith Common	**E**	**h6**	**h20**	1.00	-	-	This study
**MTD T 14360**	Great Britain: Yorkshire: Southern Washlands	**E**	**h6**	**h1**	1.00	-	-	This study
**MTD T 14361**	Great Britain: Yorkshire: Southern Washlands	**E**	**h6**	**h1**	1.00	-	-	This study
**BEV.12928**	Greece: 4 km NE Kalpaki	**5**	**l13**	**l14**	0.01	0.13	-	This study
**ZFMK 62945**	Greece: Chalkidiki: N Stratoni	**3**	**y16**	**y10**	0.01	0.21	-	Kindler *et al*. (2013)
**ZFMK 69946**	Greece: Chalkidiki: N Stratoni	**3**	**y15**	**y10**	0.00	0.40	-	Kindler *et al*. (2013)
**MTD D 19183**	Greece: Cyclades: Milos	**3**	-	**y10**	0.00	0.60	-	Kindler *et al*. (2013)
**ZFMK 85407**	Greece: Cyclades: Milos	**3**	**y15**	-	0.00	0.78	-	Kindler *et al*. (2013)
**MTD D 25827**	Greece: Cyclades: Paros	**4**	**r18**	-	-	-	-	Kindler *et al*. (2013)
**MTD T 8646**	Greece: Dadia	**7**	**gy9**	**gy1**	0.06	-	-	Kindler *et al*. (2013)
**MTD D 25843**	Greece: Kythira	**5**	**l16**	**l6**	0.01	0.12	-	Kindler *et al*. (2013)
**ZFMK 54702**	Greece: Little Prespa Lake near Florina	**5**	**l11**	**l10**	0.01	0.15	-	Kindler *et al*. (2013)
**MTD T 14327**	Greece: Milos	**3**	**y15**	**y10**	0.00	0.50	-	This study
**ZFMK 71679**	Greece: Nestos Delta	**3**	-	**y10**	0.02	0.03	-	Kindler *et al*. (2013)
**ZFMK 62500**	Greece: Peloponnesus: Itilo	**5**	**l16**	-	0.00	0.24	-	Kindler *et al*. (2013)
**MTD T 14337**	Greece: Peloponnesus: Kalogria	**5**	**l17**	**l7**	0.00	0.65	-	This study
**ZFMK 65179**	Greece: Peloponnesus: Kalogria	**5**	**l2**	-	0.00	0.03	-	Kindler *et al*. (2013)
**ZFMK 71680**	Greece: Peloponnesus: Kato Kastanea	**5**	**l16**	**l1**	0.02	0.28	-	Kindler *et al*. (2013)
**MTD T 13548**	Greece: Peloponnesus: Lake Doxa	**5**	-	**l4**	0.00	0.10	-	This study
**MTD T 14329**	Greece: Peloponnesus: Lake Stimfalia	**5**	**l16**	**l3**	0.03	0.09	-	This study
**ZFMK 92217**	Greece: Peloponnesus: Limni Strofilia near Kalogria	**5**	**l14**	**l8**	0.00	0.05	-	Kindler *et al*. (2013)
**ZFMK 82103**	Greece: Peloponnesus: near Pylos	**5**	**l18**	**l5**	0.00	0.16	-	Kindler *et al*. (2013)
**ZFMK 83029**	Greece: Peloponnesus: near Pylos	**5**	**l16**	**l2**	0.01	0.20	-	Kindler *et al*. (2013)
**ZFMK 84037**	Greece: Peloponnesus: near Pylos	**5**	**l18**	**l5**	0.03	0.04	-	Kindler *et al*. (2013)
**ZFMK 86043**	Greece: Peloponnesus: near Pylos	**5**	**l16**	**l5**	0.04	0.07	-	Kindler *et al*. (2013)
**ZFMK 86045**	Greece: Peloponnesus: near Pylos	**5**	-	**l5**	0.01	0.22	-	Kindler *et al*. (2013)
**MTD T 14336**	Greece: Peloponnesus: Strofilia	**5**	**l19**	**l8**	0.01	0.08	-	This study
**ZFMK 89415**	Greece: Samos	**7**	**gy8**	-	0.02	0.02	-	Kindler *et al*. (2013)
**MTD T 11813**	Greece: Samothrace	**7**	**gy6**	-	0.00	0.02	-	This study
**MTD T 11814**	Greece: Samothrace	**7**	**gy6**	-	0.00	0.03	-	This study
**MTD T 11815**	Greece: Samothrace	**7**	**gy6**	-	0.01	0.05	-	This study
**MTD T 11816**	Greece: Samothrace	**7**	**gy6**	**gy9**	0.00	0.16	-	This study
**MTD T 11817**	Greece: Samothrace	**7**	**gy6**	**gy9**	0.00	0.04	-	This study
**MTD T 11818**	Greece: Samothrace	**7**	**gy6**	-	0.00	0.04	-	This study
**MTD T 11819**	Greece: Samothrace	**7**	**gy6**	-	0.01	0.13	-	This study
**MTD T 11822**	Greece: Samothrace	**7**	**gy6**	-	0.01	0.12	-	This study
**MTD T 11824**	Greece: Samothrace	**7**	**gy6**	-	0.00	0.09	-	This study
**MTD T 11825**	Greece: Samothrace	**7**	**gy6**	-	0.00	0.10	-	This study
**MTD T 11826**	Greece: Samothrace	**7**	**gy6**	-	0.01	0.04	-	This study
**MTD T 11829**	Greece: Samothrace	**7**	**gy6**	-	0.00	0.03	-	This study
**MTD T 11830**	Greece: Samothrace	**7**	**gy6**	-	0.00	0.04	-	This study
**MTD T 11834**	Greece: Samothrace	**7**	**gy6**	-	0.00	0.14	-	This study
**MTD T 11837**	Greece: Samothrace	**7**	**gy6**	-	0.00	0.02	-	This study
**MTD T 9907**	Greece: Skiros	**3**	**y15**	**y10**	0.00	0.03	-	Kindler *et al*. (2013)
**MTD T 8639**	Greece: Thessaloniki	**3**	HF679875	-	0.03	0.12	-	Kindler *et al*. (2013)
**MTD D 29269**	Greece: Volos	**3**	**y15**	HF680174	0.01	0.17	-	Kindler *et al*. (2013)
**ZFMK 62956**	Greece: Xanthus: Avdira	**3**	**y15**	**y10**	0.00	0.06	-	Kindler *et al*. (2013)
**ZFMK 92957**	Greece: Xanthus: Avdira	**3**	**y15**	-	0.00	0.08	-	Kindler *et al*. (2013)
**ZFMK 62958**	Greece: Xanthus: Jasmos	**7**	**gy8**	-	0.01	0.08	-	Kindler *et al*. (2013)
**MTD T 12033**	Hungary: Balaton: Balatonszepezd	**4**	**r4**	-	0.00	0.31	-	This study
**ZFMK 58024**	Hungary: Balaton: Balatonszepezd	**4**	**r4**	-	0.00	0.31	-	Kindler *et al*. (2013)
**ZFMK 74927**	Hungary: Balaton: Tihany	**4**	**r3**	**r10**	0.00	0.64	-	Kindler *et al*. (2013)
**MTD T 11207**	Hungary: Balaton: Tihany	**4**	**r3**	**r2**	0.00	0.92	-	This study
**MTD T 11858**	Hungary: Balaton: Tihany	**4**	**r24**	-	0.00	0.72	-	This study
**MTD T 11859**	Hungary: Balaton: Tihany	**4**	**r22**	**r34**	0.00	0.91	-	This study
**ZFMK 65686**	Hungary: Barcs	**4**	**r4**	**r34**	0.00	0.65	-	Kindler *et al*. (2013)
**ZFMK 88062**	Hungary: between Sarród and Fertóujlak	**4**	**r4**	**r34**	0.00	0.40	-	Kindler *et al*. (2013)
**MTD T 12942**	Hungary: Bodrogkisfalud	**4**	**r3**	**r25**	0.01	0.82	-	This study
**ZFMK 61029**	Hungary: Györ	**4**	**r3**	**r10**	0.00	0.97	0.07	Kindler *et al*. (2013)
**ZFMK 91241**	Hungary: Hansági-főcsatorna	**4**	**r4**	**r35**	0.00	0.77	-	Kindler *et al*. (2013)
**NHMW 36337**	Hungary: Hortobagy	**4**	**r3**	-	0.00	0.86	-	This study
**MTD T 9906**	Hungary: Hortobágy	**4**	**r3**	**r3**	0.00	0.89	-	Kindler *et al*. (2013)
**MTD T 7571**	Hungary: Kunpeszér	**4**	**r4**	**r34**	0.00	0.93	-	Kindler *et al*. (2013)
**MTD T 7574**	Hungary: Kunpeszér	**4**	**r3**	**r27**	0.00	0.62	-	Kindler *et al*. (2013)
**MTD T 13547**	Hungary: Létavértes	**4**	**r3**	**r3**	0.00	0.72	-	This study
**MTD T 12016**	Hungary: Lipót	**4**	**r3**	-	0.00	0.88	-	This study
**MTD T 11862**	Hungary: near Fülöpstallas	**4**	**r30**	-	0.00	0.97	0.51	This study
**MTD T 11863**	Hungary: near Fülöpstallas	**4**	**r4**	**r39**	0.00	0.52	-	This study
**MTD T 11595**	Hungary: near Kunpeszér: Sarlóspuszta	**4**	**r4**	**r53**	0.00	0.96	0.37	This study
**MTD T 12751**	Hungary: near Tokaj	**4**	**r3**	**r3**	0.00	0.95	0.43	This study
**MTD T 12752**	Hungary: near Tokaj	**4**	**r26**	**r28**	0.00	0.72	-	This study
**MTD T 12753**	Hungary: near Tokaj	**4**	**r3**	**r3**	0.00	0.98	0.16	This study
**MTD T 12754**	Hungary: near Tokaj	**4**	**r3**	**r30**	0.01	0.95	0.71	This study
**MTD T 12755**	Hungary: near Tokaj	**4**	**r3**	**r3**	0.00	0.60	-	This study
**MTD T 12756**	Hungary: near Tokaj	**4**	**r27**	**r3**	0.00	0.87	-	This study
**MTD T 12757**	Hungary: near Tokaj	**4**	**r3**	**r13**	0.00	0.92	-	This study
**MTD T 12758**	Hungary: near Tokaj	**4**	**r3**	**r3**	0.00	0.79	-	This study
**MTD T 12759**	Hungary: near Tokaj	**4**	**r3**	**r3**	0.00	0.96	0.12	This study
**MTD T 12760**	Hungary: near Tokaj	**4**	**r3**	**r3**	0.00	0.99	0.41	This study
**MTD T 12761**	Hungary: near Tokaj	**4**	**r27**	**r3**	0.00	0.98	0.10	This study
**MTD T 12762**	Hungary: near Tokaj	**4**	**r3**	**r3**	0.00	0.74	-	This study
**MTD T 12763**	Hungary: near Tokaj	**4**	**r3**	**r3**	0.00	0.68	-	This study
**MTD T 12764**	Hungary: near Tokaj	**4**	**r27**	**r3**	0.00	0.98	0.37	This study
**MTD T 12765**	Hungary: near Tokaj	**4**	**r3**	**r3**	0.00	0.99	0.07	This study
**MTD T 12766**	Hungary: near Tokaj	**4**	**r3**	**r4**	0.00	0.76	-	This study
**MTD T 12767**	Hungary: near Tokaj	**4**	**r3**	**r6**	0.01	0.98	0.35	This study
**MTD T 12768**	Hungary: near Tokaj	**4**	**r3**	**r23**	0.00	0.99	0.58	This study
**MTD T 12769**	Hungary: near Tokaj	**4**	**r3**	**r3**	0.00	0.96	0.47	This study
**MTD T 12770**	Hungary: near Tokaj	**4**	**r3**	**r3**	0.01	0.78	-	This study
**MTD T 12771**	Hungary: near Tokaj	**4**	**r3**	**r8**	0.00	0.92	-	This study
**MTD T 12772**	Hungary: near Tokaj	**4**	**r3**	**r3**	0.00	0.94	-	This study
**MTD T 12773**	Hungary: near Tokaj	**4**	**r3**	**r3**	0.00	0.91	-	This study
**MTD T 12774**	Hungary: near Tokaj	**4**	**r3**	**r3**	0.00	0.98	0.31	This study
**MTD T 12775**	Hungary: near Tokaj	**4**	**r3**	**r6**	0.00	0.96	0.26	This study

• Table S1 continued

		mtDNA data			Microsatellite data			
**Voucher**	**Locality**	**mtDNA clade**	**ND4 haplotype**	***cyt b* haplotype**	**First STRUCTURE run**	**Second STRUCTURE run**	**Third STRUCTURE run**	**Reference**
**MTD T 12776**	Hungary: near Tokaj	4	r3	r3	0.01	0.92	-	This study
**MTD T 12777**	Hungary: near Tokaj	4	r3	r4	0.00	0.95	0.08	This study
**MTD T 12778**	Hungary: near Tokaj	4	r3	r3	0.00	0.98	0.54	This study
**MTD T 12779**	Hungary: near Tokaj	4	r3	r3	0.00	0.97	0.64	This study
**MTD T 12780**	Hungary: near Tokaj	4	r3	r3	0.00	0.81	-	This study
**ZFMK 85905**	Hungary: S Budapest	4	r4	r34	0.00	0.84	-	Kindler *et al*. (2013)
**ZFMK 82795**	Hungary: Somogyszob	4	-	r34	0.00	0.55	-	Kindler *et al*. (2013)
**MTD T 11918**	Hungary: Szeged: Lake Feher	4	r14	-	0.00	0.68	-	This study
**MTD T 11920**	Hungary: Szeged: Lake Feher	4	r3	-	0.00	0.72	-	This study
**MTD T 12014**	Hungary: Szeged: Lake Feher	4	r3	-	0.00	0.46	-	This study
**MTD T 12015**	Hungary: Szeged: Lake Feher	4	r3	-	0.00	0.72	-	This study
**MTD T 11864**	Hungary: Tiszaalpár	4	r3	r3	0.00	0.91	-	This study
**MTD T 11865**	Hungary: Tiszaalpár	4	r3	r24	0.00	0.75	-	This study
**MTD T 11867**	Hungary: Tiszaalpár	4	r3	-	0.00	0.90	-	This study
**MTD T 11979**	Hungary: Tömörd	4	r4	r34	0.00	0.54	-	This study
**MTD T 11980**	Hungary: Tömörd	4	r4	r56	0.00	0.69	-	This study
**MTD T 7594**	Hungary: Villany	4	r4	r45	0.00	0.87	-	Kindler *et al*. (2013)
**BEV.T7571**	Kazakhstan: Lake Edylsor	8	gn1	gn1	0.02	0.05	-	This study
**BEV.T7572**	Kazakhstan: Lake Edylsor	8	gn1	gn1	0.02	0.06	-	This study
**MTD T 14459**	Kosovo: Ferizaj	4	r20	r31	0.03	0.49	-	This study
**MTD T 14460**	Kosovo: Ferizaj	5	l1	l11	0.01	0.22	-	This study
**MTD T 14462**	Kosovo: Gjonaj	5	l2	-	0.00	0.13	-	This study
**MTD T 14458**	Kosovo: Nerodime e Poshtme	4	r20	-	0.01	0.26	-	This study
**MTD T 14461**	Kosovo: Topojan	3	y21	y29	0.06	-	-	This study
**MTD T 8967**	Lithuania: Marcinkonys	8	gn1	gn6	0.00	0.10	-	Kindler *et al*. (2013)
**MTD T 12787**	Lithuania: NE Šilutė	8	gn3	gn1	0.00	0.74	-	This study
**MTD T 12788**	Lithuania: NE Šilutė	8	gn3	gn1	0.00	0.20	-	This study
**MTD T 12790**	Lithuania: NE Šilutė	8	gn3	gn1	0.00	0.27	-	This study
**MTD T 8968**	Lithuania: Trasninkas	8	gn1	gn19	0.00	0.39	-	Kindler *et al*. (2013)
**MTD T 14775**	Montenegro: Bjelasica	4	r4	r1	0.06	-	-	This study
**ZFMK 92216**	Montenegro: near island Ada	5	l2	l12	0.00	0.12	-	Kindler *et al*. (2013)
**MTD T 9875**	Montenegro: Sasoviči, Zelenica, Boka kotorska	5	l2	l10	0.01	0.03	-	Kindler *et al*. (2013)
**ZFMK 92205**	Montenegro: Skutari-Lake: Limljani	5	l4	l18	0.07	-	-	Kindler *et al*. (2013)
**ZFMK 92206**	Montenegro: Skutari-Lake: Limljani	5	l2	l13	0.01	0.07	-	Kindler *et al*. (2013)
**MTD T 9908**	Montenegro: Spuz	5	l2	l12	0.02	0.03	-	Kindler *et al*. (2013)
**MTD T 13957**	Netherlands: Amstelveen	E	h1	-	1.00	-	-	This study
**MTD T 13958**	Netherlands: Amstelveen	E	h1	-	1.00	-	-	This study
**MTD T 13959**	Netherlands: Amstelveen	E	h1	h1	1.00	-	-	This study
**MTD T 12131**	Netherlands: Amsterdam: IJ	E	h1	-	1.00	-	-	This study
**MTD T 12132**	Netherlands: Amsterdam: IJ	E	h1	-	1.00	-	-	This study
**MTD T 13969**	Netherlands: Boschoord	E	h1	h1	1.00	-	-	This study
**MTD T 13970**	Netherlands: Boschoord	E	h1	h1	0.97	-	-	This study
**MTD T 13971**	Netherlands: Boschoord	E	h1	h1	1.00	-	-	This study
**MTD T 13954**	Netherlands: Durgerdam V.E.	E	h1	h29	1.00	-	-	This study
**MTD T 13955**	Netherlands: Durgerdam V.E.	E	h1	h29	1.00	-	-	This study
**MTD T 13956**	Netherlands: Durgerdam V.E.	E	h1	h29	1.00	-	-	This study
**MTD T 12129**	Netherlands: Flevoland: Kuinderbos	E	h1	-	1.00	-	-	This study
**MTD T 12133**	Netherlands: Flevoland: Kuinderbos	E	h1	-	0.98	-	-	This study
**MTD T 13960**	Netherlands: Hilversum	E	h1	h1	1.00	-	-	This study
**MTD T 13961**	Netherlands: Hilversum	E	h1	h1	1.00	-	-	This study
**MTD T 13962**	Netherlands: Hilversum	E	h1	h1	1.00	-	-	This study
**MTD T 13942**	Netherlands: Houten	E	h1	-	-	-	-	This study
**MTD T 13943**	Netherlands: Houten	E	h1	h29	1.00	-	-	This study
**MTD T 13944**	Netherlands: Houten	E	h1	h29	0.99	-	-	This study
**MTD T 13939**	Netherlands: Leersum	E	h1	h1	1.00	-	-	This study
**MTD T 13940**	Netherlands: Leersum	E	h1	-	-	-	-	This study
**MTD T 13941**	Netherlands: Leersum	E	h1	h29	1.00	-	-	This study
**MTD T 13966**	Netherlands: Oldeberkoop	E	h1	h1	1.00	-	-	This study
**MTD T 13967**	Netherlands: Oldeberkoop	E	h1	h1	1.00	-	-	This study
**MTD T 13968**	Netherlands: Oldeberkoop	E	h1	h1	1.00	-	-	This study
**MTD T 13945**	Netherlands: Oostvaardersplassen	E	h1	h1	1.00	-	-	This study
**MTD T 13946**	Netherlands: Oostvaardersplassen	E	h1	h1	0.96	-	-	This study
**MTD T 13947**	Netherlands: Oostvaardersplassen	E	h1	h1	0.98	-	-	This study
**MTD T 13963**	Netherlands: Rimburg	E	h1	h1	1.00	-	-	This study
**MTD T 13964**	Netherlands: Rimburg	E	h1	h1	0.99	-	-	This study
**MTD T 13965**	Netherlands: Rimburg	E	h1	h1	0.99	-	-	This study
**MTD T 13951**	Netherlands: Velp: Beekhuizen	E	h1	h1	0.99	-	-	This study
**MTD T 13952**	Netherlands: Velp: Beekhuizen	E	h1	h1	1.00	-	-	This study
**MTD T 13953**	Netherlands: Velp: Beekhuizen	E	h1	h1	1.00	-	-	This study
**MTD T 13948**	Netherlands: Wageningen	E	h1	h25	1.00	-	-	This study
**MTD T 13949**	Netherlands: Wageningen	E	h1	h1	1.00	-	-	This study
**MTD T 13950**	Netherlands: Wageningen	E	h1	h1	1.00	-	-	This study
**MTD T 11568**	Norway: Akershus: Ski	3	y1	y1	0.03	0.98	0.94	Kindler *et al*. (2014)
**MTD T 11569**	Norway: Akershus: Ski	3	y1	y1	0.01	0.90	-	Kindler *et al*. (2014)
**ZMUO 54-96**	Norway: Akershus: Son	3	y1	y17	0.01	0.95	0.95	Kindler *et al*. (2013)
**MTD T 11567**	Norway: Andebu: Ilestadvannet	3	y1	y5	0.04	0.95	0.83	Kindler *et al*. (2014)
**MTD T 11566**	Norway: Holmestrand: Hallingsrud	3	y1	y17	0.01	0.99	0.94	Kindler *et al*. (2014)
**ZMUO 31-83**	Norway: Oslo: Grønmo: Sølvdobla	3	y1	y17	0.00	0.99	0.95	Kindler *et al*. (2013)
**ZMUO 26-85**	Norway: Oslo: Maridalen: Skjervensaga	3	y1	y17	0.02	0.99	0.95	Kindler *et al*. (2013)
**MTD T 11571**	Norway: Østfold: Moss	3	y1	y1	0.01	0.98	0.93	Kindler *et al*. (2014)
**MTD T 11570**	Norway: southern Oslo	3	y1	y17	0.06	-	-	Kindler *et al*. (2014)
**ZMUO IH 10/2000**	Norway: Telemark: Levangshalvøya	3	y1	y17	-	-	-	Kindler *et al*. (2013)
**MTD T 11966**	Poland: Lublin: Kosyn	8	gn5	-	0.00	0.03	-	This study
**MTD T 12793**	Poland: Małopolska: Książ Wielki	4	r3	r3	0.00	0.60	-	This study
**MTD T 12830**	Poland: Małopolska: Książ Wielki	4	r3	r3	0.00	0.88	-	This study
**MTD T 12831**	Poland: Małopolska: Książ Wielki	4	r3	r3	0.00	0.84	-	This study
**MTD T 10318**	Poland: Mazovia: Kampinos National Park	4	r3	r26	0.00	0.58	-	Kindler *et al*. (2013)
**MTD T 9965**	Poland: Mazovia: Kampinos National Park	4	r3	-	0.00	0.37	-	Kindler *et al*. (2013)
**MTD T 9966**	Poland: Mazovia: Kampinos National Park	4	r3	-	0.00	0.67	-	Kindler *et al*. (2013)
**MTD T 9968**	Poland: Mazovia: Kampinos National Park	4	r3	r3	0.00	0.87	-	Kindler *et al*. (2013)
**MTD T 9969**	Poland: Mazovia: Kampinos National Park	4	r3	r26	0.00	0.90	-	Kindler *et al*. (2013)
**MTD T 9972**	Poland: Mazovia: Kampinos National Park	4	r3	r3	0.02	0.56	-	Kindler *et al*. (2013)
**MTD T 9974**	Poland: Mazovia: Kampinos National Park	4	r3	-	0.00	0.57	-	Kindler *et al*. (2013)
**BEV.7605**	Poland: Podlachia: Białowieża National Park	8	gn1	gn1	0.00	0.05	-	This study
**MTD T 10314**	Poland: Podlachia: Białowieża National Park	8	-	gn1	0.00	0.44	-	Kindler *et al*. (2013)
**MTD T 10315**	Poland: Podlachia: Białowieża National Park	8	gn1	gn2	0.00	0.28	-	Kindler *et al*. (2013)
**MTD T 10316**	Poland: Podlachia: Białowieża National Park	8	gn1	gn1	0.01	0.08	-	Kindler *et al*. (2013)
**MTD T 9975**	Poland: Podlachia: Białowieża National Park	8	gn1	-	0.00	0.12	-	Kindler *et al*. (2013)
**MTD T 9976**	Poland: Podlachia: Białowieża National Park	8	gn1	gn1	0.00	0.20	-	Kindler *et al*. (2013)
**MTD T 9977**	Poland: Podlachia: Białowieża National Park	8	gn1	gn1	0.00	0.41	-	Kindler *et al*. (2013)
**MTD T 9637**	Poland: Upper Silesia: Golczowice	4	r2	r10	0.00	0.95	0.18	Kindler *et al*. (2013)
**MTD T 10025**	Poland: Upper Silesia: Kotórz Wielki	4	r3	r10	0.00	0.95	0.09	Kindler *et al*. (2013)
**MTD T 10026**	Poland: Upper Silesia: Kotórz Wielki	3	y1	-	0.00	0.97	0.26	Kindler *et al*. (2013)
**MTD T 9635**	Poland: Upper Silesia: Krośnica	4	r4	r34	0.00	0.99	0.38	Kindler *et al*. (2013)
**MTD T 9638**	Poland: Upper Silesia: Ligota Tułowicka	4	r2	r10	0.00	0.90	-	Kindler *et al*. (2013)
**MTD T 9636**	Poland: Upper Silesia: SE Opole	4	r4	r34	0.00	0.90	-	Kindler *et al*. (2013)
**MTD T 11485**	Poland: Usedom	3	y1	y17	0.00	0.99	0.91	Kindler *et al*. (2014)
**MTD T 11486**	Poland: Usedom	3	y1	y23	0.00	0.97	0.73	Kindler *et al*. (2014)
**MTD T 8640**	Romania: Cerna Sat	4	r6	-	0.00	0.98	0.06	Kindler *et al*. (2013)
**MTD T 9889**	Romania: Chilia Veche	4	r3	-	0.00	0.70	-	Kindler *et al*. (2013)
**ZFMK 82775**	Romania: Constanța: Histria	4	-	r3	0.01	0.50	-	Kindler *et al*. (2013)
**MTD T 11604**	Romania: Crivina	4	r5	r34	0.01	0.83	-	This study
**MTD T 8650**	Romania: Geoagiu de Sus	4	r29	r3	0.01	0.80	-	Kindler *et al*. (2013)
**MTD T 13542**	Romania: Tarnovita	4	r3	r3	0.00	0.36	-	This study
**ZFMK 91040**	Russia: Bryansk	8	gn7	gn11	0.01	0.01	-	Kindler *et al*. (2013)
**ZFMK 91041**	Russia: Bryansk	8	gn1	gn1	0.00	0.01	-	Kindler *et al*. (2013)
**MTD T 13545**	Russia: Cherusti	8	gn1	gn1	0.02	0.05	-	This study
**MTD T 11970**	Russia: Dagestan: Machachkala	8	gn16	-	0.01	0.01	-	This study
**MTD T 11971**	Russia: Dagestan: Machachkala	8	gn1	-	0.00	0.04	-	This study
**ZFMK 65692**	Russia: Dagestan: Machačkala	8	gn4	gn20	0.00	0.02	-	Kindler *et al*. (2013)
**ZFMK 62936**	Russia: Dagestan: Tatayurt	8	gn1	gn9	0.01	0.01	-	Kindler *et al*. (2013)
**ZFMK 60732**	Russia: Krasnodar: Otdalyonnyy	8	gn1	gn3	0.00	0.05	-	Kindler *et al*. (2013)
**ZFMK 58029**	Russia: Krasnodar: Tuapse	8	gn1	gn21	0.02	0.03	-	Kindler *et al*. (2013)
**ZFMK 70614**	Russia: Tula: S Tula: Chopr River	8	gn1	gn1	0.07	-	-	Kindler *et al*. (2013)

• **Table S1 continued**

		mtDNA data			Microsatellite data			
**Voucher**	**Locality**	**mtDNA clade**	**ND4 haplotype**	***cyt b* haplotype**	**First STRUCTURE run**	**Second STRUCTURE run**	**Third STRUCTURE run**	**Reference**
**ZFMK 72270**	Russia: Tula: S Tula: Chopr River	8	gn1	gn7	0.00	0.01	-	Kindler *et al.* (2013)
**MTD T 14456**	Serbia: Belo Polje	4	r4	-	0.00	0.45	-	This study
**MTD T 14455**	Serbia: Belo Polje	4	r4	-	0.00	0.75	-	This study
**MTD T 14457**	Serbia: Dići	4	r4	r34	0.02	0.58	-	This study
**MTD T 12999**	Serbia: Donji Dušnik	4	r4	r54	0.05	0.56	-	This study
**MTD T 12984**	Serbia: Prohor	3	y15	y10	0.01	0.40	-	This study
**MTD T 8975**	Slovakia: between Topoľa and Runina	4	r3	-	0.00	0.93	-	Kindler *et al.* (2013)
**MTD T 12960**	Slovakia: Bodíky	4	r4	r56	0.00	0.97	0.07	This study
**MTD T 12961**	Slovakia: Bodíky	4	r3	r19	0.00	0.95	0.19	This study
**MTD T 12962**	Slovakia: Bodíky	4	r3	r10	0.00	0.97	0.06	This study
**MTD T 12963**	Slovakia: Bodíky	4	r3	r10	0.00	0.90	-	This study
**MTD T 12966**	Slovakia: Bratislava - Dúbravka	4	r3	r34	0.00	0.99	0.19	This study
**MTD T 12998**	Slovakia: Bratislava - Dúbravka	4	r4	r34	0.01	0.97	0.10	This study
**MTD T 12964**	Slovakia: Bratislava - Karlova Ves	4	r4	r34	0.00	0.95	0.08	This study
**MTD T 12967**	Slovakia: Bratislava - Karlova Ves	4	r3	r56	0.00	0.95	0.14	This study
**MTD T 14764**	Slovakia: Bratislava - Petržalka	4	r3	r10	0.00	0.99	0.08	This study
**MTD T 14765**	Slovakia: Bratislava - Petržalka	4	r3	r10	0.00	0.97	0.07	This study
**MTD T 14766**	Slovakia: Bratislava - Petržalka	4	r3	r19	0.00	0.98	0.14	This study
**MTD T 14767**	Slovakia: Bratislava - Petržalka	4	r3	r10	0.00	0.68	-	This study
**MTD T 10938**	Slovakia: Bratislava: Devínská Kobyla	4	r3	r10	0.00	0.98	0.06	This study
**MTD T 12944**	Slovakia: Bratislava: Železná Studnička	4	r3	r10	0.00	0.85	-	This study
**MTD T 12945**	Slovakia: Bratislava: Železná Studnička	4	r3	r16	0.00	0.96	0.35	This study
**MTD T 12946**	Slovakia: Bratislava: Železná Studnička	4	r3	r10	0.00	0.89	-	This study
**MTD T 12968**	Slovakia: Bratislava: Železná Studnička	4	r4	r53	0.00	0.95	0.09	This study
**MTD T 12969**	Slovakia: Bratislava: Železná Studnička	4	r3	r39	0.00	0.97	0.08	This study
**MTD T 12970**	Slovakia: Bratislava: Železná Studnička	4	r4	r57	0.00	0.98	0.05	This study
**MTD T 9017**	Slovakia: Bujakovo	4	r3	r2	0.00	0.96	0.10	Kindler *et al.* (2013)
**MTD T 8648**	Slovakia: Červený Kláštor	4	r3	r3	0.00	0.84	-	Kindler *et al.* (2013)
**MTD T 14452**	Slovakia: Chotínske Piesky	4	r8	r64	0.00	0.97	0.36	This study
**MTD T 14450**	Slovakia: Čunovo	4	r4	r34	0.00	0.98	0.41	This study
**MTD T 9014**	Slovakia: Devín	4	r3	r10	0.00	0.95	0.08	Kindler *et al.* (2013)
**MTD T 14760**	Slovakia: Devínské Jazero	4	r3	r3	0.00	0.74	-	This study
**MTD T 14761**	Slovakia: Devínské Jazero	4	r3	r19	0.00	0.97	0.08	This study
**MTD T 12996**	Slovakia: Dunajská Lužná	4	r3	r10	0.00	0.85	-	This study
**MTD T 12997**	Slovakia: Dunajská Lužná	4	r4	r37	0.00	0.93	-	This study
**MTD T 14763**	Slovakia: Gbelce: Parížské močiare	4	r4	r34	0.00	0.78	-	This study
**MTD T 14448**	Slovakia: Istragov	4	r30	r3	0.00	0.85	-	This study
**MTD T 8641**	Slovakia: Janova Lehota	4	r3	r29	0.00	0.98	0.12	Kindler *et al.* (2013)
**MTD T 9011**	Slovakia: Jurský Šúr	4	r4	r53	0.00	0.98	0.05	Kindler *et al.* (2013)
**MTD T 14754**	Slovakia: Kalinkovo - Hrušovská zdrž	4	r3	r10	0.00	0.99	0.27	This study
**MTD T 8635**	Slovakia: Kamenica nad Hronom	4	r8	-	-	-	-	Kindler *et al.* (2013)
**MTD T 12983**	Slovakia: Kosorín	4	r4	r46	0.00	0.95	0.10	This study
**MTD T 8632**	Slovakia: Kraľovany: Kraľovanská dolina	3	y1	y14	0.00	0.85	-	Kindler *et al.* (2013)
**MTD T 14772**	Slovakia: Kysak: Hornád river	4	r3	r5	0.00	0.99	0.28	This study
**MTD T 14776**	Slovakia: Liptovský Hrádok: Belá river	4	r1	r10	0.00	0.95	0.69	This study
**MTD T 14762**	Slovakia: Maňa: Žitavský luh	4	r4	r34	0.00	0.83	-	This study
**MTD T 14773**	Slovakia: Margecany: Ružín Dam	4	r3	r3	0.00	0.87	-	This study
**MTD T 12947**	Slovakia: Marianka	4	r3	r10	0.01	0.97	0.40	This study
**MTD T 12948**	Slovakia: Marianka	4	r4	r56	0.00	0.97	0.12	This study
**MTD T 12949**	Slovakia: Marianka	4	r8	r64	0.00	0.95	0.13	This study
**MTD T 12950**	Slovakia: Marianka	4	r4	r34	0.00	0.94	-	This study
**MTD T 12951**	Slovakia: Marianka	4	r4	r34	0.00	0.99	0.13	This study
**MTD T 12952**	Slovakia: Marianka	4	r3	r10	0.00	0.76	-	This study
**MTD T 12953**	Slovakia: Marianka	4	r3	r10	0.00	0.89	-	This study
**MTD T 12954**	Slovakia: Marianka	4	r4	r56	0.00	0.99	0.23	This study
**MTD T 12955**	Slovakia: Marianka	4	r3	r10	0.00	0.90	-	This study
**MTD T 12956**	Slovakia: Marianka	4	r4	r53	0.00	0.93	-	This study
**MTD T 12957**	Slovakia: Marianka	4	r4	r34	0.00	0.94	-	This study
**MTD T 12958**	Slovakia: Marianka	4	r4	r48	0.00	0.76	-	This study
**MTD T 12959**	Slovakia: Marianka	4	r4	r34	0.00	0.96	0.03	This study
**MTD T 14774**	Slovakia: Michalovce: Šíravský kanál	4	r3	r2	0.00	0.92	-	This study
**MTD T 9019**	Slovakia: Muránska Lehota	4	r31	r26	0.00	0.79	-	Kindler *et al.* (2013)
**MTD T 12943**	Slovakia: near Marcelová	4	r4	r34	-	-	-	This study
**MTD T 9018**	Slovakia: Regetovka	4	r3	r7	0.00	0.98	0.59	Kindler *et al.* (2013)
**MTD T 14449**	Slovakia: Rusovce	4	r23	-	0.00	0.98	0.05	This study
**MTD T 9016**	Slovakia: Rusovce	4	r4	r34	0.00	0.96	0.17	Kindler *et al.* (2013)
**MTD T 11594**	Slovakia: Ružomberok	3	y1	y14	0.00	0.93	-	This study
**MTD T 9009**	Slovakia: Silica: Farárova jama	4	r4	-	0.00	0.99	0.37	Kindler *et al.* (2013)
**MTD T 8637**	Slovakia: Strážne	4	r3	r3	0.00	0.84	-	Kindler *et al.* (2013)
**MTD T 9020**	Slovakia: Stupava	4	r8	r64	0.00	0.98	0.07	Kindler *et al.* (2013)
**MTD T 10937**	Slovakia: Svätý Jur	4	r4	r36	0.00	0.95	0.14	This study
**MTD T 12971**	Slovakia: Svätý Jur	4	r3	r34	0.00	0.88	-	This study
**MTD T 12972**	Slovakia: Svätý Jur	4	r3	r3	0.00	0.98	0.10	This study
**MTD T 12973**	Slovakia: Svätý Jur	4	r4	r57	0.00	0.90	-	This study
**MTD T 12974**	Slovakia: Svätý Jur	4	r3	r3	0.00	0.97	0.14	This study
**MTD T 12975**	Slovakia: Svätý Jur	4	r4	r56	0.00	0.89	-	This study
**MTD T 12976**	Slovakia: Svätý Jur	4	r4	r34	0.00	0.74	-	This study
**MTD T 12977**	Slovakia: Svätý Jur	4	r15	r34	0.00	0.95	0.12	This study
**MTD T 12978**	Slovakia: Svätý Jur	4	r4	r66	0.00	0.87	-	This study
**MTD T 12979**	Slovakia: Svätý Jur	4	r4	r50	0.00	0.89	-	This study
**MTD T 12980**	Slovakia: Svätý Jur	4	r8	r56	0.00	0.85	-	This study
**MTD T 12981**	Slovakia: Svätý Jur	4	r3	r10	0.00	0.96	0.08	This study
**MTD T 12982**	Slovakia: Svätý Jur	4	r4	r34	0.01	0.96	0.51	This study
**MTD T 8638**	Slovakia: Svetlice	4	r3	-	0.00	0.92	-	Kindler *et al.* (2013)
**MTD T 9015**	Slovakia: Tatra Mountains: Žiar	3	y1	y14	0.00	0.76	-	Kindler *et al.* (2013)
**MTD T 9022**	Slovakia: Ulič	4	r3	r3	0.00	0.98	0.38	Kindler *et al.* (2013)
**MTD T 14768**	Slovakia: Veľké Leváre	4	r32	r3	0.00	0.71	-	This study
**MTD T 9010**	Slovakia: Weitov lom	4	r3	r10	0.00	0.95	0.08	Kindler *et al.* (2013)
**MTD T 10899**	Slovenia: Bela krajina: Zjot	4	r21	r34	-	-	-	This study
**MTD T 12729**	Slovenia: Bilje: Vipava river	4	r4	r42	0.02	0.03	-	This study
**MTD T 11526**	Slovenia: Bled	4	r4	r34	0.00	0.44	-	This study
**MTD T 13540**	Slovenia: Borjana	4	r4	r34	0.01	0.10	-	This study
**MTD T 11116**	Slovenia: Borovnica	4	r4	r47	0.00	0.38	-	This study
**MTD T 11209**	Slovenia: Borovnica	4	r4	r34	0.00	0.55	-	This study
**MTD T 12783**	Slovenia: Celje: Voglajna river	4	r4	r34	0.00	0.35	-	This study
**MTD T 11535**	Slovenia: Cerknica Lake	4	r4	r38	0.02	0.30	-	This study
**MTD T 11536**	Slovenia: Cerknica Lake	4	r4	r38	0.01	0.04	-	This study
**MTD T 11537**	Slovenia: Cerknica Lake	4	r4	r38	0.01	0.09	-	This study
**MTD T 11529**	Slovenia: Cerknica Lake	4	r4	-	-	-	-	This study
**MTD T 11538**	Slovenia: Cerknica Lake	4	r4	r34	0.00	0.24	-	This study
**MTD T 13541**	Slovenia: Dragonje	4	r4	r34	0.03	0.02	-	This study
**MTD T 10887**	Slovenia: Koper: Škocjanski zatok	4	r4	r34	-	-	-	This study
**MTD T 10888**	Slovenia: Koper: Škocjanski zatok	4	r17	r60	0.00	0.16	-	This study
**MTD T 10889**	Slovenia: Koper: Škocjanski zatok	4	r17	r60	0.01	0.03	-	This study
**MTD T 10890**	Slovenia: Koper: Škocjanski zatok	4	r17	r60	0.01	0.02	-	This study
**MTD T 10891**	Slovenia: Koper: Škocjanski zatok	4	r17	r60	0.00	0.14	-	This study
**MTD T 10892**	Slovenia: Koper: Škocjanski zatok	4	r4	r34	0.10	-	-	This study
**ZFMK 65382**	Slovenia: Ljubljana	4	r4	r34	0.00	0.83	-	Kindler *et al.* (2013)
**MTD T 14346**	Slovenia: Ljubljana moor: Curnovec	4	r4	r34	0.00	0.39	-	This study
**MTD T 14349**	Slovenia: Ljubljana moor: Deponija Vic	4	r4	r34	0.03	0.52	-	This study
**MTD T 10885**	Slovenia: Ljubljana moor: Draga pri Igu	4	r4	r34	0.04	0.48	-	This study
**MTD T 10886**	Slovenia: Ljubljana moor: Draga pri Igu	4	r4	r34	0.00	0.10	-	This study
**MTD T 14347**	Slovenia: Ljubljana moor: Draga pri Igu	4	r4	r34	0.00	0.13	-	This study
**MTD T 14348**	Slovenia: Ljubljana moor: Draga pri Igu	4	r4	r40	0.00	0.06	-	This study
**MTD T 11533**	Slovenia: Ljubljana: Večna pot	4	r16	r55	-	-	-	This study
**MTD T 11212**	Slovenia: Ljubljansko barje	4	r4	r34	0.00	0.04	-	This study
**MTD T 11213**	Slovenia: Ljubljansko barje: Bistra	4	r4	r34	0.00	0.02	-	This study
**MTD T 11534**	Slovenia: Ljubljansko barje: Log pri Brezovici	4	r4	r34	0.00	0.12	-	This study
**MTD T 10900**	Slovenia: Ljubljansko barje: Pako	4	r4	r34	0.01	0.24	-	This study
**MTD T 10901**	Slovenia: Ljubljansko barje: Pako	4	r4	r34	0.00	0.12	-	This study
**MTD T 10902**	Slovenia: Ljubljansko barje: Pako	4	r4	r34	0.00	0.39	-	This study
**MTD T 10893**	Slovenia: Lukavci near Ljutomer	4	r4	r56	0.00	0.89	-	This study
**MTD T 10894**	Slovenia: Lukavci near Ljutomer	4	r4	r41	0.00	0.88	-	This study
**MTD T 12784**	Slovenia: Lukavci near Ljutomer	4	r4	r63	0.00	0.54	-	This study

• Table S1 continued

		mtDNA data			Microsatellite data			
**Voucher**	**Locality**	**mtDNA clade**	**ND4 haplotype**	***cyt b* haplotype**	**First STRUCTURE run**	**Second STRUCTURE run**	**Third STRUCTURE run**	**Reference**
MTD T 10895	Slovenia: Lukavci near Ljutomer	4	r12	r34	0.00	0.29	-	This study
MTD T 11113	Slovenia: Mele	4	r4	r34	0.00	0.30	-	This study
MTD T 11114	Slovenia: Murski Petrovci	4	r25	r34	0.03	0.65	-	This study
MTD T 11527	Slovenia: near Maribor: Cigonca Forest	4	r8	r33	0.00	0.80	-	This study
MTD T 11530	Slovenia: near Maribor: Racki Ribnik	4	r4	r34	0.01	0.34	-	This study
MTD T 11208	Slovenia: Podlipa	4	r4	r34	0.05	0.04	-	This study
MTD T 11531	Slovenia: Pragersko	4	r4	r41	0.00	0.06	-	This study
MTD T 12178	Slovenia: Sentjur	4	r4	r46	0.00	0.71	-	This study
MTD T 11528	Slovenia: Slovenia: Divača	4	r4	r49	0.00	0.16	-	This study
MTD T 11115	Slovenia: Tišina	4	r4	r51	0.00	0.78	-	This study
MTD T 11210	Slovenia: Zadolje	4	r17	r64	0.06	-	-	This study
ZMH R09056	Sweden: Blekinge: Augerum	3	y1	y20	0.00	0.99	0.93	This study
ZFMK 36114	Sweden: Gotland	3	y1	y5	-	-	-	Kindler *et al*. (2013)
ZFMK 38356	Sweden: Gotland	4	r3	r10	-	-	-	Kindler *et al*. (2013)
BEV.6418	Sweden: Gotland: near Burgsvik	8	gn1	gn1	0.00	0.99	-	This study
MTD T 11582	Sweden: Närke: Klockhammar	3	y1	y5	0.01	0.99	0.88	Kindler *et al*. (2014)
ZMH R09209	Sweden: Öland: between Stenasa and Resmo: Mockelmossen	3	y1	y20	0.00	0.99	0.94	This study
MTD T 10920	Sweden: Öland: Halltorps Hage	3	y36	y17	0.05	0.99	0.95	Kindler *et al*. (2014)
MTD T 10921	Sweden: Öland: Halltorps Hage	3	y1	y5	0.01	0.99	0.93	Kindler *et al*. (2014)
MTD T 10922	Sweden: Öland: Halltorps Hage	3	y1	y5	0.01	0.99	0.92	Kindler *et al*. (2014)
ZMH R09024	Sweden: Skåne: Maglehem	3	y20	-	-	-	-	This study
ZMH R09026	Sweden: Skåne: Olseröd	3	y1	-	-	-	-	This study
MTD T 10919	Sweden: Småland: Kråksmåla	3	y25	y28	0.00	0.99	0.95	Kindler *et al*. (2014)
MTD T 11580	Sweden: Södermanland: near Boo	3	y1	y5	0.01	0.99	0.93	Kindler *et al*. (2014)
MTD T 11579	Sweden: Södermanland: near Nyköping	3	y1	y17	0.01	0.99	0.95	Kindler *et al*. (2014)
MTD T 11581	Sweden: Södermanland: near Nyköping	3	y1	y17	0.00	0.99	0.94	Kindler *et al*. (2014)
BEV.6417	Sweden: Uppland: Forsmark	3	y1	y25	-	-	-	This study
BEV.6419	Sweden: Uppland: near Uppsala: Östervåla	3	y4	y25	0.00	0.99	0.85	This study
BEV.6420	Sweden: Uppland: near Uppsala: Östervåla	3	y6	y28	-	-	-	This study
MTD T 14859	Sweden: Västergötland: Rånnaväg	3	y1	y17	0.02	0.99	0.96	This study
MTD T 14860	Sweden: Västergötland: Vegby	3	y1	y1	0.01	0.99	0.96	This study
ROH01	Switzerland: Aargau: Aarau: Rohr	E	h1	h19	1.00	-	-	This study
ROH02	Switzerland: Aargau: Aarau: Rohr	E	h9	h23	1.00	-	-	This study
ROH04	Switzerland: Aargau: Aarau: Rohr	E	h1	h19	1.00	-	-	This study
BAD01	Switzerland: Aargau: Bad Zurzach	3	y1	y1	0.16	-	-	This study
BAD02	Switzerland: Aargau: Bad Zurzach	3	y1	y1	0.05	0.98	0.09	This study
BAD03	Switzerland: Aargau: Bad Zurzach	3	y1	y1	0.02	0.98	0.21	This study
BAD04	Switzerland: Aargau: Bad Zurzach	3	y1	y1	0.30	-	-	This study
BAD05	Switzerland: Aargau: Bad Zurzach	3	y1	y1	0.01	0.99	0.11	This study
BAD06	Switzerland: Aargau: Bad Zurzach	3	y1	y1	0.22	-	-	This study
BAD07	Switzerland: Aargau: Bad Zurzach	3	y1	y1	0.18	-	-	This study
BAD08	Switzerland: Aargau: Bad Zurzach	3	y1	y1	0.35	-	-	This study
BAD09	Switzerland: Aargau: Bad Zurzach	3	y1	y1	0.19	-	-	This study
BAD10	Switzerland: Aargau: Bad Zurzach	3	y1	y1	0.29	-	-	This study
KGN01	Switzerland: Aargau: Böttstein	E	h1	h1	1.00	-	-	This study
KGN02	Switzerland: Aargau: Böttstein	E	h1	h1	1.00	-	-	This study
BRU01	Switzerland: Aargau: Brugg	E	h1	h20	1.00	-	-	This study
BRU02	Switzerland: Aargau: Brugg	E	h9	h23	1.00	-	-	This study
BRU03	Switzerland: Aargau: Brugg	E	h1	h1	1.00	-	-	This study
AGO01	Switzerland: Aargau: Eiken	E	h9	h23	0.79	-	-	This study
AGO02	Switzerland: Aargau: Eiken	E	h9	h23	0.67	-	-	This study
AGO03	Switzerland: Aargau: Eiken	E	h9	h23	0.90	-	-	This study
GIP01	Switzerland: Aargau: Gippingen	E	h1	h1	0.98	-	-	This study
GIP02	Switzerland: Aargau: Gippingen	E	h9	h23	0.86	-	-	This study
GIP03	Switzerland: Aargau: Gippingen	E	h9	h23	1.00	-	-	This study
GIP04	Switzerland: Aargau: Gippingen	E	h9	h23	1.00	-	-	This study
GIP05	Switzerland: Aargau: Gippingen	E	h1	h1	1.00	-	-	This study
KLI01	Switzerland: Aargau: Klingnau	E	h1	h19	1.00	-	-	This study
MUE06	Switzerland: Aargau: Merenschwand	unknown	-	-	1.00	-	-	This study
RIM01	Switzerland: Aargau: Rietheim	3	y37	y1	0.15	-	-	This study
ROT01	Switzerland: Aargau: Rottenschwil	E	h1	h10	1.00	-	-	This study
REG02	Switzerland: Basel-Landschaft: Binningen	E	h1	h11	0.98	-	-	This study
MBL14	Switzerland: Basel-Landschaft: Liestal	E	h1	h11	1.00	-	-	This study
REG01	Switzerland: Basel-Landschaft: Pratteln	E	h1	h1	1.00	-	-	This study
BBE01	Switzerland: Bern: Airport Bern	E	h1	h1	1.00	-	-	This study
BBE02	Switzerland: Bern: Airport Bern	E	h1	h18	1.00	-	-	This study
BBE03	Switzerland: Bern: Airport Bern	E	h1	h1	1.00	-	-	This study
BBE04	Switzerland: Bern: Airport Bern	E	h1	h23	0.99	-	-	This study
BBE05	Switzerland: Bern: Airport Bern	E	h1	h23	1.00	-	-	This study
BBE06	Switzerland: Bern: Airport Bern	E	h1	h1	1.00	-	-	This study
MBE10	Switzerland: Bern: Airport Bern	E	h1	h1	1.00	-	-	This study
MBE11	Switzerland: Bern: Mühleberg	E	h1	h2	1.00	-	-	This study
MTD T 10083	Switzerland: Bern: near Bern	E	h1	h1	0.99	-	-	Kindler *et al*. (2013)
MTD T 10085	Switzerland: Bern: near Meiringen: Gadmental	E	-	h1	1.00	-	-	Kindler *et al*. (2013)
MTD T 10086	Switzerland: Bern: near Meiringen: Gadmental	E	h3	-	1.00	-	-	Kindler *et al*. (2013)
MBE09	Switzerland: Bern: Sutz-Lattrigen	E	h1	h23	1.00	-	-	This study
MTD T 10084	Switzerland: Fribourg: between Neuchâtel, Bern and Biel: Grosses Moos	E	h1	-	0.99	-	-	Kindler *et al*. (2013)
MTD T 10095	Switzerland: Fribourg: between Neuchâtel, Bern and Biel: Grosses Moos	E	h1	-	1.00	-	-	Kindler *et al*. (2013)
CHU01	Switzerland: Graubünden: Chur	E	h1	h9	1.00	-	-	This study
BGR01	Switzerland: Graubünden: Filisur	E	h1	h9	1.00	-	-	This study
FLA01	Switzerland: Graubünden: Fläsch	E	h1	h9	1.00	-	-	This study
FLA02	Switzerland: Graubünden: Fläsch	E	h1	h9	0.99	-	-	This study
FLA03	Switzerland: Graubünden: Fläsch	E	h1	h9	1.00	-	-	This study
LAA01	Switzerland: Graubünden: Laax	E	h1	h9	1.00	-	-	This study
LAA02	Switzerland: Graubünden: Laax	E	h1	h9	0.99	-	-	This study
JUR01	Switzerland: Jura: Beurnevésin	unknown	-	-	1.00	-	-	This study
JUR02	Switzerland: Jura: Beurnevésin	unknown	-	-	1.00	-	-	This study
SHO01	Switzerland: Schaffhausen: Hallau	3	y1	y1	0.24	-	-	This study
SHO02	Switzerland: Schaffhausen: Hallau	3	y1	y1	0.22	-	-	This study
SHO03	Switzerland: Schaffhausen: Hallau	3	y1	y1	0.22	-	-	This study
SAR01	Switzerland: Schaffhausen: Hemishofen	3	y1	y1	0.04	0.99	0.52	This study
SAR02	Switzerland: Schaffhausen: Hemishofen	3	y37	y1	0.13	-	-	This study
SAR03	Switzerland: Schaffhausen: Hemishofen	3	y1	y1	0.03	0.98	0.11	This study
SHF01	Switzerland: Schaffhausen: Herblingen	3	y1	y1	0.07	-	-	This study
SHF02	Switzerland: Schaffhausen: Herblingen	3	y1	y12	0.12	-	-	This study
SHF03	Switzerland: Schaffhausen: Herblingen	3	y1	y1	0.08	-	-	This study
MTD D 30567	Switzerland: St. Gallen: Altenrhein	3	y1	HF679958	0.07	-	-	Kindler *et al*. (2013)
ALT01	Switzerland: St. Gallen: Altstätten	E	h1	h9	0.97	-	-	This study
ALT02	Switzerland: St. Gallen: Altstätten	E	h1	h9	0.97	-	-	This study
ALT03	Switzerland: St. Gallen: Altstätten	E	h1	h1	0.80	-	-	This study
ALT04	Switzerland: St. Gallen: Altstätten	E	h1	h9	0.99	-	-	This study
ALT05	Switzerland: St. Gallen: Altstätten	E	h1	h9	0.79	-	-	This study
BAL01	Switzerland: St. Gallen: Balgach	E	h1	h9	0.56	-	-	This study
BAL02	Switzerland: St. Gallen: Balgach	E	h1	h9	0.96	-	-	This study
BAL03	Switzerland: St. Gallen: Balgach	3	y40	y1	0.66	-	-	This study
BAL04	Switzerland: St. Gallen: Balgach	E	h1	h9	0.98	-	-	This study
BAL05	Switzerland: St. Gallen: Balgach	E	h1	h9	0.71	-	-	This study
BCZ01	Switzerland: St. Gallen: Berneck	unknown	-	-	0.37	-	-	This study
BCZ02	Switzerland: St. Gallen: Berneck	3	y1	-	0.25	-	-	This study
BCZ03	Switzerland: St. Gallen: Berneck	3	y1	y1	0.27	-	-	This study
BCZ04	Switzerland: St. Gallen: Berneck	E	h1	h16	1.00	-	-	This study
BCZ05	Switzerland: St. Gallen: Berneck	E	h1	h22	0.99	-	-	This study
BCZ06	Switzerland: St. Gallen: Berneck	E	h1	h22	1.00	-	-	This study
BCZ07	Switzerland: St. Gallen: Berneck	E	h1	h10	1.00	-	-	This study
BCZ08	Switzerland: St. Gallen: Berneck	E	h1	h1	0.94	-	-	This study
BCZ09	Switzerland: St. Gallen: Berneck	E	h1	h1	1.00	-	-	This study
BCZ10	Switzerland: St. Gallen: Berneck	E	h1	h1	0.99	-	-	This study
BCZ11	Switzerland: St. Gallen: Berneck	E	h1	h1	0.99	-	-	This study
DIE01	Switzerland: St. Gallen: Diepoldsau	3	y40	y1	0.64	-	-	This study
DIE02	Switzerland: St. Gallen: Diepoldsau	3	y40	y1	0.66	-	-	This study
DIE03	Switzerland: St. Gallen: Diepoldsau	3	y1	y1	0.93	-	-	This study
DIE04	Switzerland: St. Gallen: Diepoldsau	unknown	-	-	0.61	-	-	This study
MTD T 10088	Switzerland: St. Gallen: Lake Constance: near Thal	3	y1	-	0.25	-	-	Kindler *et al*. (2013)
OBE01	Switzerland: St. Gallen: Oberuzwil	3	y1	y1	0.05	0.98	0.62	This study

• Table S1 continued

Voucher	Locality	mtDNA clade (mtDNA data)	ND4 haplotype (mtDNA data)	*cyt b* haplotype (mtDNA data)	First STRUCTURE run (Microsatellite data)	Second STRUCTURE run (Microsatellite data)	Third STRUCTURE run (Microsatellite data)	Reference
OBE02	Switzerland: St. Gallen: Oberuzwil	3	y1	y1	0.07	-	-	This study
OBE03	Switzerland: St. Gallen: Oberuzwil	3	y42	y36	0.08	-	-	This study
OBE04	Switzerland: St. Gallen: Oberuzwil	3	y42	y36	0.05	0.98	0.04	This study
OBE05	Switzerland: St. Gallen: Oberuzwil	3	y1	y1	0.01	0.99	0.25	This study
OBE06	Switzerland: St. Gallen: Oberuzwil	3	y1	y1	0.01	0.99	0.24	This study
OBE07	Switzerland: St. Gallen: Oberuzwil	3	y42	y36	0.04	0.98	0.06	This study
QUI01	Switzerland: St. Gallen: Quinten	E	h1	h12	1.00	-	-	This study
SMN01	Switzerland: St. Gallen: Schmerikon	E	h1	h13	1.00	-	-	This study
SMN02	Switzerland: St. Gallen: Schmerikon	E	h1	h13	0.99	-	-	This study
SMN03	Switzerland: St. Gallen: Schmerikon	E	h1	h3	0.91	-	-	This study
SEW01	Switzerland: St. Gallen: Sennwald	E	h1	h9	1.00	-	-	This study
NEB01	Switzerland: St. Gallen: Thal	3	y40	y1	0.28	-	-	This study
NEB02	Switzerland: St. Gallen: Thal	E	h1	h9	0.24	-	-	This study
NEB03	Switzerland: St. Gallen: Thal	3	y1	y1	0.32	-	-	This study
NEB04	Switzerland: St. Gallen: Thal	3	y1	y1	0.27	-	-	This study
NEB05	Switzerland: St. Gallen: Thal	E	h1	h9	0.31	-	-	This study
THA01	Switzerland: St. Gallen: Thal	3	y1	y1	0.22	-	-	This study
THA02	Switzerland: St. Gallen: Thal	3	y40	y36	0.36	-	-	This study
THA03	Switzerland: St. Gallen: Thal	3	y1	y1	0.21	-	-	This study
WIL01	Switzerland: St. Gallen: Will	3	y1	y36	0.16	-	-	This study
AAD01	Switzerland: Thurgau: Aadorf	3	y37	y1	0.28	-	-	This study
AAD02	Switzerland: Thurgau: Aadorf	3	y37	y1	0.45	-	-	This study
AAD03	Switzerland: Thurgau: Aadorf	3	y37	y1	0.23	-	-	This study
JUV01	Switzerland: Thurgau: Alterswilen	3	y28	y1	0.01	0.99	0.12	This study
JUV02	Switzerland: Thurgau: Alterswilen	3	y29	y1	0.12	-	-	This study
JUV03	Switzerland: Thurgau: Alterswilen	3	y27	y1	0.11	-	-	This study
JUV04	Switzerland: Thurgau: Alterswilen	3	y1	y1	0.36	-	-	This study
JUV05	Switzerland: Thurgau: Alterswilen	3	y27	y1	0.11	-	-	This study
JUV06	Switzerland: Thurgau: Alterswilen	3	y27	y1	0.23	-	-	This study
NWN01	Switzerland: Thurgau: Alterswilen	3	y1	y1	0.19	-	-	This study
NWN02	Switzerland: Thurgau: Alterswilen	3	y1	y1	0.16	-	-	This study
BUR01	Switzerland: Thurgau: Bürglen	3	y1	y1	0.17	-	-	This study
BUR02	Switzerland: Thurgau: Bürglen	3	y1	y1	0.16	-	-	This study
FRA01	Switzerland: Thurgau: Frauenfeld	3	y1	y1	0.23	-	-	This study
FRA02	Switzerland: Thurgau: Frauenfeld	3	y37	y1	0.09	-	-	This study
KRE01	Switzerland: Thurgau: Kreuzlingen	3	y1	y1	0.09	-	-	This study
KRE02	Switzerland: Thurgau: Kreuzlingen	3	y1	y1	0.29	-	-	This study
KRE03	Switzerland: Thurgau: Kreuzlingen	3	y1	y1	0.19	-	-	This study
MUE01	Switzerland: Uri: Erstfeld	E	h1	h1	1.00	-	-	This study
GEN01	Switzerland: Vaud: Begnins	E	h1	h1	1.00	-	-	This study
MTD T 10079	Switzerland: Vaud: Vinzel	E	h1	-	1.00	-	-	Kindler *et al*. (2013)
DAT01	Switzerland: Zürich: Dätwil	3	y37	y1	0.16	-	-	This study
DAT02	Switzerland: Zürich: Dätwil	3	y37	y1	0.20	-	-	This study
DUB01	Switzerland: Zürich: Dübendorf	3	y1	y1	0.98	-	-	This study
DUB02	Switzerland: Zürich: Dübendorf	3	y1	y1	0.99	-	-	This study
DUB03	Switzerland: Zürich: Dübendorf	3	y1	y1	0.98	-	-	This study
DUB04	Switzerland: Zürich: Dübendorf	3	y1	y1	0.99	-	-	This study
FIS01	Switzerland: Zürich: Fischenthal	E	h1	h1	0.86	-	-	This study
FIS02	Switzerland: Zürich: Fischenthal	E	h1	h9	1.00	-	-	This study
FIS03	Switzerland: Zürich: Fischenthal	E	h1	h1	1.00	-	-	This study
FCH01	Switzerland: Zürich: Flaach	3	y37	y1	0.02	0.96	0.09	This study
FCH02	Switzerland: Zürich: Flaach	3	y1	y1	0.12	-	-	This study
FCH03	Switzerland: Zürich: Flaach	3	y37	y1	0.17	-	-	This study
FCH04	Switzerland: Zürich: Flaach	3	y1	y1	0.14	-	-	This study
FCH05	Switzerland: Zürich: Flaach	unknown	-	-	0.17	-	-	This study
FCH06	Switzerland: Zürich: Flaach	3	y1	y1	0.20	-	-	This study
GAT01	Switzerland: Zürich: Gattikon	E	h11	h23	0.98	-	-	This study
GAT02	Switzerland: Zürich: Gattikon	E	h1	h1	0.98	-	-	This study
PFA01	Switzerland: Zürich: Lake Pfäffikon	E	h1	h26	0.97	-	-	This study
MUE07	Switzerland: Zürich: Langnau am Albis	E	h1	h1	0.99	-	-	This study
MAR01	Switzerland: Zürich: Marthalen	3	y38	y42	0.08	-	-	This study
MAR02	Switzerland: Zürich: Marthalen	3	y1	y35	0.25	-	-	This study
MAR03	Switzerland: Zürich: Marthalen	3	y38	y42	0.04	0.97	0.09	This study
NEE01	Switzerland: Zürich: Neerach	3	y1	y1	0.31	-	-	This study
NEE02	Switzerland: Zürich: Neerach	3	y1	y1	0.16	-	-	This study
NEE03	Switzerland: Zürich: Neerach	3	y1	y1	0.23	-	-	This study
NEE04	Switzerland: Zürich: Neerach	3	y1	y32	0.05	0.99	0.13	This study
OBF01	Switzerland: Zürich: Obfelden	E	h1	h1	1.00	-	-	This study
OBF02	Switzerland: Zürich: Obfelden	E	h1	h10	1.00	-	-	This study
PFU01	Switzerland: Zürich: Pfungen	3	y33	y1	0.46	-	-	This study
PFU02	Switzerland: Zürich: Pfungen	3	y1	y1	0.31	-	-	This study
PFU03	Switzerland: Zürich: Pfungen	3	y1	y1	0.28	-	-	This study
STA01	Switzerland: Zürich: Stäfa	E	h1	h1	0.91	-	-	This study
STA02	Switzerland: Zürich: Stäfa	E	h1	h1	0.96	-	-	This study
STA03	Switzerland: Zürich: Stäfa	E	h1	h1	0.88	-	-	This study
STA04	Switzerland: Zürich: Stäfa	E	h1	h1	0.97	-	-	This study
MUE02	Switzerland: Zürich: Stallikon	unknown	-	-	0.96	-	-	This study
TAT01	Switzerland: Zürich: Thalheim an der Thur	3	y1	y1	0.12	-	-	This study
GRF01	Switzerland: Zürich: Uster	E	h1	h1	0.78	-	-	This study
WEI01	Switzerland: Zürich: Weiach	3	y1	y1	0.16	-	-	This study
WEI02	Switzerland: Zürich: Weiach	3	y1	y36	0.21	-	-	This study
WEI03	Switzerland: Zürich: Weiach	3	y33	y1	0.02	0.96	0.06	This study
MUE03	Switzerland: Zürich: Wettswil am Albis	unknown	-	-	0.99	-	-	This study
MUE04	Switzerland: Zürich: Wettswil am Albis	unknown	-	-	0.99	-	-	This study
WIN01	Switzerland: Zürich: Winterthur: Sennhof	E	h1	h1	0.44	-	-	This study
WIN02	Switzerland: Zürich: Winterthur: Sennhof	E	h1	h1	0.21	-	-	This study
WIN03	Switzerland: Zürich: Winterthur: Sennhof	E	h1	h1	0.41	-	-	This study
WIN04	Switzerland: Zürich: Winterthur: Sennhof	E	h1	h1	0.31	-	-	This study
ZEL01	Switzerland: Zürich: Zell	E	h1	h1	0.38	-	-	This study
ZEL02	Switzerland: Zürich: Zell	E	h1	h1	0.31	-	-	This study
ZEL03	Switzerland: Zürich: Zell	E	h1	h1	0.25	-	-	This study
ZEL04	Switzerland: Zürich: Zell	E	h1	h1	0.44	-	-	This study
ZEL05	Switzerland: Zürich: Zell	E	h1	h1	0.58	-	-	This study
ZEL06	Switzerland: Zürich: Zell	E	h1	h1	0.38	-	-	This study
ZEL07	Switzerland: Zürich: Zell	E	h1	h1	0.39	-	-	This study
ZHC01	Switzerland: Zürich: Zürich	E	h1	h1	0.96	-	-	This study
ZHC02	Switzerland: Zürich: Zürich	E	h1	h1	1.00	-	-	This study
ZHN01	Switzerland: Zürich: Zürich: Affoltern	E	h1	h12	1.00	-	-	This study
ZHN02	Switzerland: Zürich: Zürich: Affoltern	E	h1	h3	0.83	-	-	This study
KLO01	Switzerland: Zürich: Zürich: Airport	3	y1	y1	0.31	-	-	This study
KLO02	Switzerland: Zürich: Zürich: Airport	3	y1	y1	0.25	-	-	This study
KLO03	Switzerland: Zürich: Zürich: Airport	3	y1	y1	0.41	-	-	This study
KLO04	Switzerland: Zürich: Zürich: Airport	E	h1	h12	0.35	-	-	This study
KLO05	Switzerland: Zürich: Zürich: Airport	3	y1	y1	0.22	-	-	This study
KLO06	Switzerland: Zürich: Zürich: Airport	3	y1	y1	0.34	-	-	This study
KLO07	Switzerland: Zürich: Zürich: Airport	E	h1	h12	0.26	-	-	This study
KLO08	Switzerland: Zürich: Zürich: Airport	3	y1	y1	0.44	-	-	This study
KLO09	Switzerland: Zürich: Zürich: Airport	3	y1	y1	0.26	-	-	This study
KLO10	Switzerland: Zürich: Zürich: Airport	3	y1	y1	0.33	-	-	This study
ZFMK 47034	Turkey: Aegean Region: between Söke and Doğanbey	7	-	gy12	0.02	0.03	-	Kindler *et al*. (2013)
ZFMK 82946	Turkey: Aegean Region: Dalyan	7	HF679889	gy13	0.01	0.03	-	Kindler *et al*. (2013)
MTD D 25227	Turkey: Aegean Region: Selçuk	7	gy11	HF680188	0.02	0.01	-	Kindler *et al*. (2013)
MTD D 25229	Turkey: Aegean Region: Selçuk	7	gy8	-	0.15	-	-	Kindler *et al*. (2013)
MTD D 25230	Turkey: Aegean Region: Selçuk	7	gy8	HF680189	0.03	0.02	-	Kindler *et al*. (2013)
NHMW 38053	Turkey: Black Sea Region: 10 km E Gerede	8	gn14	gn16	0.02	0.02	-	This study
ZFMK 71143	Turkey: Black Sea Region: between Hopa and Arhavi	8	-	gn15	0.00	0.02	-	Kindler *et al*. (2013)
ZFMK 71144	Turkey: Black Sea Region: between Hopa and Arhavi	8	-	gn14	0.00	0.01	-	Kindler *et al*. (2013)
ZFMK 71145	Turkey: Black Sea Region: Borçka	8	gn13	gn18	0.00	0.02	-	Kindler *et al*. (2013)
BEV.T7392	Turkey: Marmara Region: Beşevler	7	gy7	gy8	0.00	0.02	-	This study
MTD D 42725	Ukraine: Crimea: Luchyste	8	gn2	gn8	0.00	0.03	-	Kindler *et al*. (2013)
MTD D 42724	Ukraine: Oblast Herson: Hola Prystan': Herois'ke	8	gn6	gn12	0.00	0.04	-	Kindler *et al*. (2013)

Museum acronyms of vouchers for Table S1:

BEV – Laboratoire de Biogéographie et Ecologie des Vertébrés, Centre d'Ecologie Fonctionnelle & Evolutive, Montpellier
LMNM – Landesmuseum Natur und Mensch, Oldenburg
MTD D – Museum of Zoology, Senckenberg Dresden (Herpetological Collection)
MTD T – Museum of Zoology, Senckenberg Dresden (Tissue Collection)
MWLK – Museum der Westlausitz, Kamenz
NHMW – Naturhistorisches Museum Wien
NME – Naturkundemuseum Erfurt
SMF – Forschungsinstitut und Naturmuseum Senckenberg, Frankfurt a.M.
SMNS – Staatliches Museum für Naturkunde Stuttgart
SMNK – Staatliches Museum für Naturkunde Karlsruhe
ZFMK – Zoologisches Forschungsmuseum Alexander Koenig, Bonn
ZMB – Museum für Naturkunde Berlin
ZMH – Zoologisches Museum Hamburg
ZMUO – Zoologisk museum, Universitetet i Oslo
ZSM – Zoologische Staatssammlung München
ZSUM – Zoologische Sammlung der Philipps-Universität Marburg

Table S2. Akaike Information Criterion (AIC) scores for fitted clines under different models using the R package HZAR (Derryberry *et al.* 2014). Bold values in blue indicate the best-fit model from the 15-model comparison.

	Contact zone I		Contact zone II	
	**Microsatellites**	**mtDNA**	**Microsatellites**	**mtDNA**
**Null Model**	152.846	184.261	68.427	254.247
**Model 1**	38.966	**17.566**	11.670	**74.262**
**Model 2**	22.132	n/a	**11.366**	n/a
**Model 3**	17.449	21.839	15.249	78.504
**Model 4**	18.120	25.038	18.909	n/a
**Model 5**	17.692	n/a	18.865	82.518
**Model 6**	23.491	30.390	22.930	86.930
**Model 7**	29.795	21.169	15.795	78.360
**Model 8**	**13.501**	n/a	15.365	n/a
**Model 9**	19.169	26.013	18.782	82.610
**Model 10**	36.967	21.694	15.573	78.360
**Model 11**	26.258	n/a	15.353	n/a
**Model 12**	21.600	26.034	19.168	82.724
**Model 13**	15.214	21.694	15.111	78.360
**Model 14**	15.295	n/a	15.119	n/a
**Model 15**	20.469	25.999	19.306	82.733

Table S3. European Nucleotide Archive (ENA) accession numbers of ND4 and cyt *b* haplotypes.

ND4 haplotype	Accession number	cyt *b* haplotype	Accession number
helvetica			
**h1**	LT839092	**h1**	LT839229
**h2**	LT839093	**h2**	LT839230
**h3**	LT839094	**h3**	LT839231
**h4**	LT839095	**h4**	LT839232
**h5**	LT839096	**h5**	LT839233
**h6**	LT839097	**h6**	LT839234
**h7**	LT839098	**h7**	LT839235
**h8**	LT839099	**h8**	LT839236
**h9**	LT839100	**h9**	LT839237
**h10**	LT839101	**h10**	LT839238
**h11**	LT839102	**h11**	LT839239
**h12**	LT839103	**h12**	LT839240
		**h13**	LT839241
		**h14**	LT839242
		**h15**	LT839243
		**h16**	LT839244
		**h17**	LT839245
		**h18**	LT839246
		**h19**	LT839247
		**h20**	LT839248
		**h21**	LT839249
		**h22**	LT839250
		**h23**	LT839251
		**h24**	LT839252
		**h25**	LT839253
		**h26**	LT839254
		**h27**	LT839255
		**h28**	LT839256
		**h29**	LT839257
**Yellow lineage (lineage 3 of Kindler *et al.*, 2013)**			
**y1**	LT839104	**y1**	LT839258
**y2**	LT839105	**y2**	LT839259
**y3**	LT839106	**y3**	LT839260
**y4**	LT839107	**y4**	LT839261
**y5**	LT839108	**y5**	LT839262
**y6**	LT839109	**y6**	LT839263
**y7**	LT839110	**y7**	LT839264
**y8**	LT839111	**y8**	LT839265
**y9**	LT839112	**y9**	LT839266
**y10**	LT839113	**y10**	LT839267
**y11**	LT839114	**y11**	LT839268
**y12**	LT839115	**y12**	LT839269
**y13**	LT839116	**y13**	LT839270
**y14**	LT839117	**y14**	LT839271
**y15**	LT839118	**y15**	LT839272
**y16**	LT839119	**y16**	LT839273
**y17**	LT839120	**y17**	LT839274
**y18**	LT839121	**y18**	LT839275
**y19**	LT839122	**y19**	LT839276
**y20**	LT839123	**y20**	LT839277
**y21**	LT839124	**y21**	LT839278

• Table S3 continued			
**Yellow lineage (lineage 3 of Kindler *et al*., 2013)**			
**y22**	LT839125	**y22**	LT839279
**y23**	LT839126	**y23**	LT839280
**y24**	LT839127	**y24**	LT839281
**y25**	LT839128	**y25**	LT839282
**y26**	LT839129	**y26**	LT839283
**y27**	LT839130	**y27**	LT839284
**y28**	LT839131	**y28**	LT839285
**y29**	LT839132	**y29**	LT839286
**y30**	LT839133	**y30**	LT839287
**y31**	LT839134	**y31**	LT839288
**y32**	LT839135	**y32**	LT839289
**y33**	LT839136	**y33**	LT839290
**y34**	LT839137	**y34**	LT839291
**y35**	LT839138	**y35**	LT839292
**y36**	LT839139	**y36**	LT839293
**y37**	LT839140	**y37**	LT839294
**y38**	LT839141	**y38**	LT839295
**y39**	LT839142	**y39**	LT839296
**y40**	LT839143	**y40**	LT839297
**y41**	LT839144	**y41**	LT839298
**y42**	LT839145	**y42**	LT839299
**y43**	LT839146		
**Red lineage (lineage 4 of Kindler *et al*., 2013)**			
**r1**	LT839147	**r1**	LT839300
**r2**	LT839148	**r2**	LT839301
**r3**	LT839149	**r3**	LT839302
**r4**	LT839150	**r4**	LT839303
**r5**	LT839151	**r5**	LT839304
**r6**	LT839152	**r6**	LT839305
**r7**	LT839153	**r7**	LT839306
**r8**	LT839154	**r8**	LT839307
**r9**	LT839155	**r9**	LT839308
**r10**	LT839156	**r10**	LT839309
**r11**	LT839157	**r11**	LT839310
**r12**	LT839158	**r12**	LT839311
**r13**	LT839159	**r13**	LT839312
**r14**	LT839160	**r14**	LT839313
**r15**	LT839161	**r15**	LT839314
**r16**	LT839162	**r16**	LT839315
**r17**	LT839163	**r17**	LT839316
**r18**	LT839164	**r18**	LT839317
**r19**	LT839165	**r19**	LT839318
**r20**	LT839166	**r20**	LT839319
**r21**	LT839167	**r21**	LT839320
**r22**	LT839168	**r22**	LT839321
**r23**	LT839169	**r23**	LT839322
**r24**	LT839170	**r24**	LT839323
**r25**	LT839171	**r25**	LT839324
**r26**	LT839172	**r26**	LT839325
**r27**	LT839173	**r27**	LT839326
**r28**	LT839174	**r28**	LT839327
**r29**	LT839175	**r29**	LT839328
**r30**	LT839176	**r30**	LT839329
**r31**	LT839177	**r31**	LT839330

• Table S3 continued

**Red lineage (lineage 4 of Kindler *et al*., 2013)**			
**r32**	LT839178	**r32**	LT839331
**r33**	LT839179	**r33**	LT839332
		**r34**	LT839333
		**r35**	LT839334
		**r36**	LT839335
		**r37**	LT839336
		**r38**	LT839337
		**r39**	LT839338
		**r40**	LT839339
		**r41**	LT839340
		**r42**	LT839341
		**r43**	LT839342
		**r44**	LT839343
		**r45**	LT839344
		**r46**	LT839345
		**r47**	LT839346
		**r48**	LT839347
		**r49**	LT839348
		**r50**	LT839349
		**r51**	LT839350
		**r52**	LT839351
		**r53**	LT839352
		**r54**	LT839353
		**r55**	LT839354
		**r56**	LT839355
		**r57**	LT839356
		**r58**	LT839357
		**r59**	LT839358
		**r60**	LT839359
		**r61**	LT839360
		**r62**	LT839361
		**r63**	LT839362
		**r64**	LT839363
		**r65**	LT839364
		**r66**	LT839365
**Lilac lineage (lineage 5 of Kindler *et al*., 2013)**			
**l1**	LT839180	**l1**	LT839366
**l2**	LT839181	**l2**	LT839367
**l3**	LT839182	**l3**	LT839368
**l4**	LT839183	**l4**	LT839369
**l5**	LT839184	**l5**	LT839370
**l6**	LT839185	**l6**	LT839371
**l7**	LT839186	**l7**	LT839372
**l8**	LT839187	**l8**	LT839373
**l9**	LT839188	**l9**	LT839374
**l10**	LT839189	**l10**	LT839375
**l11**	LT839190	**l11**	LT839376
**l12**	LT839191	**l12**	LT839377
**l13**	LT839192	**l13**	LT839378
**l14**	LT839193	**l14**	LT839379
**l15**	LT839194	**l15**	LT839380
**l16**	LT839195	**l16**	LT839381
**l17**	LT839196	**l17**	LT839382
**l18**	LT839197	**l18**	LT839383

• Table S3 continued			
**Lilac lineage (lineage 5 of Kindler *et al*., 2013)**			
**l19**	LT839198	**l19**	LT839384
**l20**	LT839199	**l20**	LT839385
		**l21**	LT839386
		**l22**	LT839387
**Grey lineage (lineage 7 of Kindler *et al*., 2013)**			
**gy1**	LT839200	**gy1**	LT839388
**gy2**	LT839201	**gy2**	LT839389
**gy3**	LT839202	**gy3**	LT839390
**gy4**	LT839203	**gy4**	LT839391
**gy5**	LT839204	**gy5**	LT839392
**gy6**	LT839205	**gy6**	LT839393
**gy7**	LT839206	**gy7**	LT839394
**gy8**	LT839207	**gy8**	LT839395
**gy9**	LT839208	**gy9**	LT839396
**gy10**	LT839209	**gy10**	LT839397
**gy11**	LT839210	**gy11**	LT839398
		**gy12**	LT839399
		**gy13**	LT839400
**Green lineage (lineage 8 of Kindler *et al*., 2013)**			
**gn1**	LT839211	**gn1**	LT839401
**gn2**	LT839212	**gn2**	LT839402
**gn3**	LT839213	**gn3**	LT839403
**gn4**	LT839214	**gn4**	LT839404
**gn5**	LT839215	**gn5**	LT839405
**gn6**	LT839216	**gn6**	LT839406
**gn7**	LT839217	**gn7**	LT839407
**gn8**	LT839218	**gn8**	LT839408
**gn9**	LT839219	**gn9**	LT839409
**gn10**	LT839220	**gn10**	LT839410
**gn11**	LT839221	**gn11**	LT839411
**gn12**	LT839222	**gn12**	LT839412
**gn13**	LT839223	**gn13**	LT839413
**gn14**	LT839224	**gn14**	LT839414
**gn15**	LT839225	**gn15**	LT839415
**gn16**	LT839226	**gn16**	LT839416
		**gn17**	LT839417
		**gn18**	LT839418
		**gn19**	LT839419
		**gn20**	LT839420
		**gn21**	LT839421
**Lineage C of Kindler *et al*. (2013)**			
**c1**	LT839227	**c1**	LT839422
		**c2**	LT839423
**Lineage F of Kindler *et al*. (2013)**			
**f1**	LT839228	**f1**	LT839424

Table S4. Used microsatellite loci. For primer sequences, annealing temperature and multiplex-sets, see Pokrant *et al.* (2016).

Locus	Repeat motif	Allele size range [bp]	Number of alleles	Original reference
**Natnat09**	$(AC)_{22}$	80 - 144	25	Meister *et al.* (2009)
**Natnat05**	$(GT)_{16}$	136 - 194	23	Meister *et al.* (2009)
**µNt8new**	$(AC)_{15}$	75 - 123	20	Meister *et al.* (2009)
**Nsµ3**	$(ATCT)_{14}$ $ATC(CA)_{20}$	139 - 457	39	Prosser *et al*. (1999)
**µNt3**	$(AC)_{16}$	111 - 163	24	Gautschi *et al*. (2000)
**µNt7**	$(AC)_{17}$	164 - 212	25	Gautschi *et al.* (2000)
**30**	$(CA)_{14}$	225 - 271	20	Burns & Houlden (1999)
**Natnat11**	$(GA)_{13}$	102 - 228	25	Meister *et al.* (2009)
**Natnat06**	$(GT)_{21}$	145 - 185	16	Meister *et al.* (2009)
**Tbu A09**	$(AC)_{7}$	110 - 146	17	Sloss *et al.* (2012)
**3TS**	$(GATA)_{19}$	186 - 270	20	Garner *et al.* (2002)
**Eobµ1**	$(TG)_{21}$	120 - 142	12	Blouin-Demers & Gibbs (2003)
**Eobµ13**	$(AC)_{20}$	118 - 162	19	Blouin-Demers & Gibbs (2003)

Table S5. Genetic diversity of STRUCTURE clusters based on 13 microsatellite loci. *n* number of individuals, n_A number of alleles, $n_{\bar{A}}$ average number of alleles per locus, n_P number of private alleles, AR allelic richness, H_O average observed heterozygosity, H_E average expected heterozygosity. F_{IS} inbreeding coefficient, F_{ST} fixation index. Individuals with mixed ancestries not considered. All F_{IS} and F_{ST} values were statistically significant.

Microsatellites									
**Cluster**	***n***	n_A	$n_{\bar{A}}$	n_P	***AR***	H_O	H_E	F_{IS}	F_{ST}
First STRUCTURE run									
helvetica	350	156	12.000	45	4.505	0.404	0.548	0.265	0.40
**Eastern lineages***	953	230	17.692	119	5.761	0.501	0.612	0.182	
Second STRUCTURE run									
**Yellow + red lineage**	502	121	9.308	19	7.185	0.453	0.510	0.112	0.18
**Adjacent lineages****	100	186	14.308	84	14.006	0.510	0.712	0.285	
Third STRUCTURE run									
**Yellow**	175	83	6.385	12	6.025	0.423	0.467	0.095	0.11
**Red**	122	102	7.846	31	7.722	0.501	0.555	0.098	

*Yellow, red, lilac, grey and green lineages
**Lilac, grey and green lineages

Table S6. Simulated data. Twenty samples of each parental group were chosen as pure parental genotypes. Using this data, 20 genotypes of each hybrid class (F_1, F_2 and the two backcrosses) were modelled in HYBRIDLAB and analyzed with STRUCTURE. Individuals with Q values ≥ 92% were reliably identified as pure *helvetica*, individuals with Q values ≥ 95% as eastern grass snakes. Regarding the yellow and red lineages, the differentiation from backcrosses was more difficult. Although there was a misassignment rate of 5% for the pure yellow lineage, we decided to treat grass snakes with at least 80% cluster membership as pure yellow because otherwise the misidentification rate with backcrosses would be too high. Individuals with at least 83% were treated as pure red.

	**Parental group 1**	**Parental group 2**	**F_1 (referred to parental 1 cluster)**	**F_1 (referred to parental 2 cluster)**	**F_2 (referred to parental 1 cluster)**	**F_2 (referred to parental 2 cluster)**	**Backcross parental 1**	**Backcross parental 2**
	helvetica	**Eastern lineages**						
**Average *Q* score**	0.965	0.971	0.493	0.507	0.467	0.533	0.772	0.765
**SD**	0.013	0.007	0.034	0.034	0.095	0.095	0.078	0.066
**Minimum *Q***	0.921	0.954	0.444	0.444	0.328	0.319	0.634	0.661
**Maximum *Q***	0.997	0.979	0.556	0.556	0.681	0.672	0.968	0.912
**Misassignment**	if Q < 92%: 0%	if Q < 95%: 0%	if Q < 92%: 0%	if Q < 95%: 0%	if Q < 92%: 0%	if Q < 95%: 0%	if Q < 92%: 5%	if Q < 95%: 0%
	**Yellow lineage**	**Red lineage**						
**Average *Q* score**	0.880	0.890	0.480	0.520	0.580	0.420	0.740	0.720
**SD**	0.049	0.033	0.149	0.149	0.192	0.192	0.140	0.148
**Minimum *Q***	0.770	0.825	0.191	0.310	0.206	0.150	0.442	0.353
**Maximum *Q***	0.935	0.940	0.690	0.809	0.850	0.794	0.925	0.917
**Misassignment**	if Q < 80%: 5%	if Q < 83%: 0%	if Q < 80%: 0%	if Q < 83%: 0%	if Q < 80%: 15%	if Q < 83%: 0%	if Q < 80%: 40%	if Q < 83%: 30%

References

Blouin-Demers, G. & Gibbs, H.L. Isolation and characterization of microsatellite loci in the black rat snake (*Elaphe obsoleta*). *Mol. Ecol. Notes* **3**, 98–99 (2003).

Burns, E.L. & Houlden, B.A. Isolation and characterization of microsatellite markers in the broad-headed snake *Hoplocephalus bungaroides. Mol. Ecol.* **8**, 520–521(1999).

Derryberry, E.P., Derryberry, G.E., Maley, J.M. & Brumfield, R.T. HZAR: hybrid zone analysis using an R software package. *Mol. Ecol. Resour.* **14**, 652–663 (2014).

Fritz, U., Corti, C. & Päckert, M. Mitochondrial DNA sequences suggest unexpected phylogenetic position of Corso-Sardinian grass snakes (*Natrix cetti*) and do not support their species status, with notes on phylogeography and subspecies delineation of grass snakes. *Org. Divers. Evol.* **12**, 71–80 (2012).

Garner, T.W.J. *et al.* Geographic variation of multiple paternity in the common garter snake (*Thamnophis sirtalis*). *Copeia* **2002**, 15–23 (2002).

Gautschi, B., Widmer, A. & Koella, J. Isolation and characterization of microsatellite loci in the dice snake (*Natrix tessellata*). *Mol. Ecol.* **9**, 2192–2193 (2000).

Kabisch, K. *Natrix natrix* (Linnaeus, 1758) – Ringelnatter in *Handbuch der Reptilien und Amphibien Europas. Band 3/IIA: Schlangen II* (ed Böhme, W.), 513–580 (Aula-Verlag, 1999).

Kindler, C. *et al.* Mitochondrial phylogeography, contact zones and taxonomy of grass snakes (*Natrix natrix*, *N. megalocephala*). *Zool. Scr.* **42**, 458–472 (2013).

Kindler, C., Bringsøe, H. & Fritz, U. Phylogeography of grass snakes (*Natrix natrix*) all around the Baltic Sea: implications for the Holocene colonization of Fennoscandia. *Amphibia-Reptilia* **35**, 413–424 (2014).

Kreiner, G. *Die Schlangen Europas* (Chimaira, 2007).

Meister, B., Armbruster, F.J., Frauenfelder, N. & Bauer, B. Novel microsatellite loci in the grass snake (*Natrix natrix*) and cross-amplification in the dice snake (*Natrix tessellata*). *Mol. Ecol. Resour.* **9**, 604–606 (2009).

Pokrant, F. *et al.* Integrative taxonomy provides evidence for the species status of the Ibero-Maghrebian grass snake *Natrix astreptophora*. *Biol. J. Linn. Soc.* **118**, 873–888 (2016).

Prosser, M.R., Gibbs, H.L. & Weatherhead, P.J. Microgeographic population genetic structure in the northern water snake, *Nerodia sipedon sipedon* detected using microsatellite DNA loci. *Mol. Ecol.* **8**, 329–333 (1999).

Sloss, B.L., Schuurman, G.W., Paloski, R.A., Boyle, O.D. & Kapfer, J.M. Novel microsatellite loci for studies of *Thamnophis* gartersnake genetic identity and hybridization. *Conserv. Genet. Resour.* **4**, 383–386 (2012).
